# Biological Activity of Stilbenoids Against Fungal, Parasitic, and Viral Pathogens

**DOI:** 10.3390/molecules31050830

**Published:** 2026-03-01

**Authors:** Aristodemos-Theodoros Periferakis, Argyrios Periferakis, Lamprini Troumpata, Konstantinos Periferakis, Andreea-Elena Scheau, Adrian Iftime, Ana Caruntu, Ioana Anca Badarau, Constantin Caruntu, Cristian Scheau

**Affiliations:** 1 Department of Physiology, The “Carol Davila” University of Medicine and Pharmacy, 050474 Bucharest, Romania; 2 Elkyda, Research & Education Centre of Charismatheia, 17675 Athens, Greece; 3 Akadimia of Ancient Greek and Traditional Chinese Medicine, 16675 Athens, Greece; 4 Pan-Hellenic Organization of Educational Programs (P.O.E.P.), 17236 Athens, Greece; 5 Department of Radiology and Medical Imaging, “Foisor” Clinical Hospital of Orthopaedics, Traumatology and Osteoarticular TB, 021382 Bucharest, Romania; 6 Department of Biophysics, The “Carol Davila” University of Medicine and Pharmacy, 050474 Bucharest, Romania; 7 Department of Oral and Maxillofacial Surgery, “Carol Davila” Central Military Emergency Hospital, 010825 Bucharest, Romania; 8 Faculty of Dental Medicine, “Titu Maiorescu” University, 031593 Bucharest, Romania; 9 Department of Dermatology, “Prof. N.C. Paulescu” National Institute of Diabetes, Nutrition and Metabolic Diseases, 011233 Bucharest, Romania

**Keywords:** astringin, resveratrol, pinosylvin, piceatannol, piceid, pterostilbene, antifungal effects, antiparasitic effects, antiviral effects, toxicity, infection, pathophysiology, mechanisms

## Abstract

Stilbenoids are plant-derived chemical compounds that are classified as phytoalexins; recent focus has been drawn, especially on astringin, piceid, piceatannol, pterostilbene, pinosylvin, and resveratrol. These substances have been extensively studied for a variety of beneficial properties, including their effects on pathogenic microorganisms, parasites, and viruses. In their antifungal capacity, they are effective against *Aspergillus* spp., *Botrytis* spp., *Candida* spp., *Trichophyton* spp., and other fungi; tested stilbenoids have exhibited fungicidal and fungistatic effects, and inhibition of biofilm formation. Against parasites, they are effective against *Echinococcus* spp., *Leishmania* spp., *Schistosoma* spp., *Trypanosoma* spp., *Toxoplasma* spp., among others. Relevant action mechanisms include a reduction in parasitic enzymatic activity and inhibition of proliferation. They are also effective against different DNA and RNA viruses; the relevant mechanisms comprise reduction in viral replication, inhibition of viral genome expression, and viral attachment to cells. The toxicity of stilbenoids has been reviewed in recent papers, and, in most cases, the effective concentrations applied are well below the toxicity limit.

## 1. Introduction

In the latter half of the 20th century and afterwards, the development and mass production of a number of different pharmacological agents and drugs have provided treatment and curative solutions for a variety of different pathologies of different etiologies [1,2]. However, even when such solutions are effective, they may be associated with side effects or adverse effects of varying severity, which limit their use [3,4,5,6,7,8,9]. Resistance to pharmacotherapy raises further issues in the treatment of infectious diseases [10,11,12]. It is mostly this resistance to drugs that has fueled the search for alternative therapeutic agents; plant-derived compounds are identified based on the meticulous analysis of the chemical constituents of their extracts alongside ethnomedical and ethnobotanical reports [13,14,15,16,17,18,19,20,21]. Numerous compounds have been identified in phytochemical studies, such as kaempferol, capsaicin, pinosylvin, quercetin, and piperine, among others [22,23,24,25,26,27,28,29,30,31,32].

One of the most studied categories of natural compounds is stilbene phytoalexins. Stilbenes are plant-synthesized polyphenolic substances whose purpose is to defend the plants from bacterial and fungal infections [33], hence their classification as phytoalexins [34]. They play a very important role in the plant kingdom, being a crucial line of defense against infections and harsh climate conditions [35,36,37,38].

Stilbenoids have a common core represented by an ethylene moiety in the middle of two benzene rings; the principal structural factor determining their functionality is the location and number of hydroxyl groups [39,40]. They are derived from the phenylpropanoid pathway; their biosynthesis is catalyzed by stilbene synthase, which has evolved from chalcone synthase, whose activity in plants is tissue-specific and development stage-specific [36,41,42].

The most important stilbenoid phytoalexins are astringin, resveratrol, pinosylvin, piceid, piceatannol, and pterostilbene, even though hundreds of stilbenoids occur naturally via different chemical reactions [43]. This review focuses on the most well-known and researched stilbenoids (Table 1).

**Table 1:** Basic data on the stilbenoids discussed in the text, organized alphabetically.

Stilbenoid	Chemical Name	Chemical Class	Water Solubility	First Isolation/Identification	References
Astringin	3-β-D-glucoside of piceatannol	Glycosides	730 μg/mL	1962	[44,45]
Piceid	resveratrol 3-β-mono-D-glucoside		1260 μg/mL	1940	[46,47]
Piceatannol	3′,4′,3,5-tetrahydroxy-trans-stilbene	Aglycones	161 μg/mL	1948/1963	[48,49,50]
Pinosylvin	3,5-dihydroxy-trans-stilbene		Practically insoluble ^1^	1939	[51,52,53]
Pterostilbene	trans-3,5-dimethoxy-4′-hydroxystilbene		12.7 μg/mL	1940	[54,55,56]
Resveratrol	3,5,4′-trihydroxy-trans-stilbene		52.2 μg/mL	1940	[48,57]

^1^ Small values comparable to other stilbenoids when complexed with bile salts.

Resveratrol (3,5,4′-trihydroxy-trans-stilbene) is the most well-known and studied stilbene [58,59]; usually, in the human food chain, it is contained in grapes and wine products, where the trans form prevails [60,61]. A number of researchers have concentrated on determining the content of resveratrol and other polyphenols in different types of wine [62]. Resveratrol has been under intense research for its different beneficial properties in human health [63,64].

Astringin can be derived from pinosylvin, resveratrol, or piceatannol via different metabolic pathways. Astringin is relatively unstable in the air, and this precludes its facile use in standard antimicrobial tests [65]. As of yet, there is not a significant corpus of scientific literature on the properties and potential uses of astringin [45].

Piceid, also known as polydatin [47], is a major derivative of resveratrol in grape juices [66]; pterostilbene (trans-3,5-dimethoxy-4′-hydroxystilbene) is a structural analog of resveratrol, with demonstrated anti-inflammatory, antioxidant and anticancer capacity [54,67,68], which has been isolated from a number of plants, many of which have been used in traditional medical systems [69,70]. The very close similarity of pterostilbene to resveratrol means that it can potentially have similar health benefits, and be even more potent in some regards [71,72]; for instance, its beneficial properties in glucose and lipid metabolisms have been demonstrated in a series of experiments, and their applications would have system-wide impact [73,74,75,76].

Pinosylvin (3,5-dihydroxy-trans-stilbene) is a stilbenoid polyphenol that is principally found in plants of the Pinaceae family. Numerous studies have demonstrated its various health-related benefits, such as its antioxidant, anti-inflammatory, and anticancer potential, while Pinaceae plants themselves have been used in many traditional medical systems [51].

Piceatannol (3,3′,4,5′-tetrahydroxy-trans-stilbene) has poor water solubility as it is relatively nonpolar, with the trans isomer being abundant [49]. It was originally isolated from *Vouacapoua americana* [77]. Recent research has revealed its anticarcinogenic, antioxidant, antidiabetic, and anti-inflammatory potential [78], among others.

**Figure 1:**
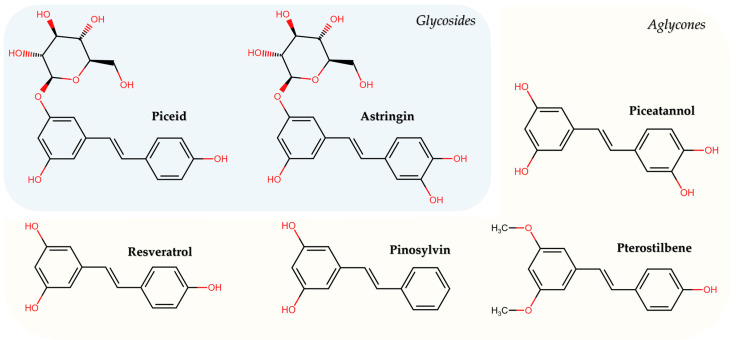
Chemical structures of the most relevant stilbenoids. Marvin JS was used for drawing, displaying, and characterizing chemical structures, Marvin JS 25.3.0, Chemaxon (http://chemaxon.com/ (accessed on 6 February 2026)), based on public chemical data [79].

The chemical structures of the above-mentioned substances are represented in Figure 1.

All the aforementioned stilbenoids can be found in numerous different plant species (Table 2).

Various methods have been proposed for the production of different stilbenoids, as they are promising targets for cosmetic applications and as food supplements [126]. For example, the market for resveratrol is expanding, in parallel with the scientific interest [126].

In this review, we aim to provide a comprehensive overview of the antifungal, antiviral, and antiparasitic activity of the aforementioned stilbenoids, along with the known associated mechanisms; antibacterial applications have been discussed by a number of authors (e.g., [127,128,129,130,131,132,133,134,135]). An overview of the medical relevance and summary of the presented actions are also provided, alongside potential issues of toxicity and bioavailability.

## 2. Antifungal Activity of Stilbenoids

Fungal infections still represent an appreciable threat to human health and well-being, even if the number of pathogenic fungi is lower compared to that of pathogenic bacteria [136], and most fungal infections are not life-threatening in the absence of risk factors [137,138,139]. At the same time, resistance to antifungal agents represents another therapeutic challenge [140,141,142]. Currently, there is a large corpus of data on the antifungal potential of the discussed stilbenoids, albeit mostly in vitro (Table 3).

### 2.1. Antifungal Activity Against Aspergillus spp.

#### 2.1.1. *Aspergillus flavus*

This fungus, alongside others of its species, is associated with the production of harmful metabolites called aflatoxins [154,155]. They can contaminate food sources such as nuts and grapes [154,155]. As such, this microorganism represents a notable threat to public health safety and causes significant economic losses due to the measures that have to be taken in order to prevent aflatoxicosis [156]. Pterostilbene was found to have good antifungal activity against *A. flavus*. Apart from an EC_50_ lower than that of natamycin, it could also inhibit the infection of peanuts, at concentrations of 250 and 500 μg/mL [143].

#### 2.1.2. *Aspergillus fumigatus*

This is a ubiquitous saprophytic fungus, which can cause aspergillosis, one of the most clinically relevant opportunistic fungal infections [157,158]. While immunocompetent hosts are typically resistant to infection, a variety of diseases from bronchopulmonary aspergillosis to invasive aspergillosis can affect immunocompromised patients [159,160]. Due to its high clinical impact and increasing resistance to antifungals, A. fumigatus is a major target for the development of novel agents [161].

The antifungal effects of knotwood extracts of different *Pinus* species in general must be attributable, at least in part, to their content of pinosylvin and its derivatives. These are more active when in their pure form, but the action mechanism remains unknown [144].

### 2.2. Antifungal Activity Against Candida albicans

This fungus is a common commensal yeast and is one of the leading causes of opportunistic fungal infections [162]. It may colonize the oral cavity, genitourinary mucosa, and gastrointestinal tract without causing disease; transitioning to a pathogenic state can occur in host immunity disruptions or microbial imbalances [163]. It may be responsible for a wide spectrum of infections, from superficial mucocutaneous candidiasis to life-threatening infections and disseminated disease [164].

Pinosylvin, both as a pure compound and as a component in extracts, proved effective against *C. albicans*, although the precise mechanism was not elucidated [144]. A number of other researchers have also demonstrated its effectiveness against this pathogen [145,165].

Pterostilbene was tried both in vivo and in vitro by Li et al. [148], and was found to be able to inhibit biofilm formation in both settings. Biofilm inhibition, both for pterostilbene and some analogues, was confirmed by the research of Hu et al. [121]; at lower concentrations, pterostilbene could inhibit biofilm formation, and at higher concentrations, it was also effective against mature biofilm. An experimental study, conducted in vitro by Kingsbury et al. [147], where astringin, pinosylvin, pterostilbene, and resveratrol were tested, revealed that only pterostilbene inhibited *C. albicans* growth in low concentrations. They also noted the potential of combining fungicidal phytochemicals with antifungal drugs.

The fungicidal potential of resveratrol, and some of its structural analogues, against *C. albicans* has long been recognized [145,149]. It also exhibits a good biofilm formation inhibitory potential and could disrupt already formed biofilm in the study of Kolouchová et al. [150]; the same results were obtained for pterostilbene in this study. Surprisingly, Weber et al. [166] reported no effectiveness of resveratrol against *C. albicans* and other *Candida* species. Another study by Wang et al. [151] found that resveratrol, in either its cis or trans confirmation, did not have an appreciable antifungal effect until very high concentrations in excess of 1000 μg/mL, at which point its solubility decreased. It did, however, exhibit a synergistic effect with azole antifungals.

### 2.3. Antifungal Activity Against Cladosporium herbarum

This microorganism contributes to the environmental fungal bioaerosols, and it is a well-recognized source of inhalant allergens associated with allergic rhinitis, asthma exacerbations, and hypersensitive reactions [167,168,169]. Human infections are rare, but may occur in immunocompromised individuals; otherwise, there is little available evidence of its pathogenicity. Pinosylvin was found to have a notable effectiveness, with an EC_50_ of just 10 mg/mL [118]; similar results were recorded by Pacher et al. [119].

### 2.4. Antifungal Activity Against Epidermophyton floccosum

This pathogen is among the ones most often associated with dermatophytia [170,171,172], although recently, a reduced prevalence has been noted [171,172]. The associated conditions are tinea at the level of the trunk (corporis), the groin (cruris), the feet (pedis), and the nails (ungium) [171,172], with the first three being the most common [171]. Against *E. floccosum*, resveratrol was found to be effective at relatively low concentrations, compared to its toxicity limit. The precise mechanism, although unknown, may have something to do with inhibition of the functionality of membrane proteins [152].

### 2.5. Antifungal Activity Against Trichophyton spp.

#### 2.5.1. *Trichophyton mentagrophytes*

This fungus has a global distribution, affecting humans and animals alike [173], causing diverse dermatophytosis [174]. There has recently been a high rise in the prevalence of genotype VII [173]; the microorganism thus constitutes an emerging zoonosis [173] which can pose a problem for public health, particularly in males of low socioeconomic background [175]. Resistance to commonly used antifungals, such as fluconazole and terbinafine, has been recorded [176,177,178].

Against *T. mentagrophytes*, as well as against other *Trichophyton* spp., resveratrol had a fungicidal effect, although the precise mechanism remains unknown [152]. The relatively low concentrations needed are far below the toxicity limit and allow for effective local application.

#### 2.5.2. *Trichophyton rubrum*

This microorganism is the one most commonly implicated in superficial infections of fungal etiology all over the world [177,178], being more active during the summer and usually affecting people over 50 years old, especially males [178]. The main associated pathology is tinea pedis, and to a lesser extent, tinea ungium and tinea cruris [178]. Although it is currently considered sensitive to the action of terbinafine [177], a drug-resistant strain was recently isolated in China [179].

Against *T. rubrum*, as well as against other *Trichophyton* spp., resveratrol had a fungicidal effect, although the precise mechanism remains unknown [152]. Given that the necessary concentration for a meaningful inhibition is relatively low, and far below the known toxicity limits, it is believed that local application will be effective. Amongst all the stilbene phytoalexins, only pinosylvin and pterostilbene were found to inhibit *T. rubrum* growth in reasonably low concentrations [147].

#### 2.5.3. *Trichophyton tonsurans*

This fungus is among the main causes of tinea capitis [180] and tinea corporis [180,181], although there also exist asymptomatic carriers [181]. An association with contact sports has been established [181]. Resistance to antifungal agents, such as fluconazole and terbinafine, has been noted [182,183].

Against *T. tonsurans*, as well as against other *Trichophyton* spp., resveratrol had a fungicidal effect, although the precise mechanism remains unknown. Bearing in mind that the concentration needed is relatively low, there is the possibility of local application without exceeding the toxicity limit [152].

### 2.6. Antifungal Activity Against Trichosporon spp.

#### 2.6.1. *Trichosporon beigelii*

The potential of this fungus to cause severe infections, resistant to treatment, in immunocompromised patients has been demonstrated since the 1990s [184]. While very rare, infections with this opportunistic pathogen may even cause severe infective endocarditis, where the difficulty in treatment is complicated by difficulties in early diagnosis [185]. Against this pathogen, resveratrol was found to be as potent as amphotericin B [146].

#### 2.6.2. *Trichosporon cutaneum*

This fungus is associated with white piedra and onychomycosis [186,187]. Its biofilm production plays a major role in its virulence [187], and it has been shown to act as an opportunistic pathogen in patients with suppressed immune responses [186]. Some level of resistance to fluconazole has been identified [187]. Against *T. cutaneum*, as well as against other *Trichosporon* spp., resveratrol caused inhibition of biofilm formation and disruption of existing biofilm [153].

## 3. Antiparasitic Activity of Stilbenoids

Parasites represent a widespread cause of morbidity and mortality, especially in areas where they are endemic [188,189,190]; their spread may be facilitated by a number of factors [191,192]. They therefore represent a considerable potential burden of disease [193,194,195], and some parasites have developed resistance to antiparasitic drugs [196,197]. The need to develop novel therapeutic approaches is thus apparent; stilbenoids may represent potent antiparasitic agents, based on recent research results (Table 4).

### 3.1. Antiparasitic Activity Against Echinococcus spp.

*Echinococcus* spp., and its most clinically relevant species, *E. granulosus*, are responsible for echinococcosis, characterized by hydatid cysts in the internal organs of humans or other animals, who serve as the intermediate hosts [218]. The disease presents a worldwide distribution [218] and generally has a favorable prognosis if detected early [219], but may carry increased morbidity and mortality in certain patient subsets [220,221,222]. Current therapeutic agents may potentially be associated with serious adverse effects [223].

The effects of resveratrol against Echinococcus species were recently studied by the team of Loos et al. [198] both in vivo and in vitro. In vitro results revealed that resveratrol is effective against *E. granulosus* and *E. multilocularis*, as it causes ultrastructural damage, modulates gene expression, and promotes autophagy; the in vivo arm of the research, however, did not reveal resveratrol to have appreciable antiparasitic activity [198].

### 3.2. Antiparasitic Activity Against Hymenolepis diminuta

*Hymenolepis diminuta*, the rat tapeworm, infects humans in tropical regions with poor sanitation [224]. Infections are rare and usually affect children [225,226]. Praziquantel is the drug of choice, but there are still gaps in the understanding of the epidemiology and pathophysiology of the disease [227]. The extract from *Carex baccans* was found to reduce the worm burden in infected rats, and resveratrol, when isolated further, was equally effective. Due to their good tolerance and effectiveness, they seem promising potential antiparasitic solutions [199].

### 3.3. Antiparasitic Activity Against Naegleria fowleri

*Naegleria fowleri* is a free-living parasite that can complete its life cycle outside of a host [228,229]. It causes primary amoebic meningoencephalitis, which is often fatal [229,230,231]. Infection occurs in freshwater via the nasal passages, and the treatment is difficult, owing to a lack of widely tried therapies and problems in timely diagnosis. The current treatment regimen comprises a host of drugs, namely amphotericin B, azithromycin, fluconazole, rifampin, dexamethasone, and miltefosine, but the scheme has unreliable efficiency and high toxicity [232,233].

Piceid was amongst the most effective phytochemicals isolated from *Gossypium hirsutum*, i.e., cotton plant, and tested in silico for effectiveness against *N. fowleri*. Based on molecular docking studies, piceid was found to obey all the rules for drug likeness against which it was tested and, importantly, showed good gastrointestinal absorption. Therefore, it is a promising candidate for an S-adenosyl-L-homocysteine hydrolase inhibitor, which is a basic metabolic enzyme of the parasite [200].

### 3.4. Antiparasitic Activity Against Leishmania spp.

Leishmaniasis is endemic in tropical and subtropical regions and is mostly transmitted through sand flies [234]. It presents with a variety of symptoms and can have several clinical forms [235]. Over 20 different species of Leishmania are infectious, and transmission vectors persist in different areas, despite efforts at eradication [236]. The current regimen of antileishmanial drugs is based on antimonials, which, while effective, have significant adverse effects and are associated with increasing resistance [237,238,239].

Piceatannol proved to be moderately effective against the extracellular forms of *L. donovani*, *L. infantum*, and *L. major*, but was more active than the reference compound in the extracellular form of *L. donovani* [84]. In the more recent experiments of Passos et al. [202], in mice, it proved to be effective against both the amastigote and promastigote stages of *L. amazonensis*, with an IC_50_ far lower than the toxicity limit for healthy cells. Pterostilbene was found to be effective against amastigote and promastigote stages of *L. amazonensis*, but could not alter its cell cycle, in contrast to piceatannol; it was found to decrease the production of pro-inflammatory mediators in infected macrophages and reduce NO levels [202].

Resveratrol was shown to be potent against *L. major* against promastigotes and intracellular amastigotes, although the mechanism of its action was not elucidated [205]; another research effort some years later confirmed its antileishmanial activity in vitro, noting that it was most probably a result of its cytotoxicity against host cells, rather than a direct antiparasitic action [206]. Against *L. amazonensis*, resveratrol exhibited a synergistic effect with amphotericin B, resulting in inhibition of proliferation and incidental parasite death [201]. Another team performed molecular and computational docking studies to explore the effects of resveratrol and some of its analogues in trypanothione reductase, the enzyme that is associated with resistance to miltefosine and pentavalent antimonials [240]; it was found that these compounds could effectively inhibit the enzyme, and the analogues displayed better GIT absorption potential [204]. Resveratrol analogues in vitro were found to exert their antileishmanial effects via a multitude of mechanisms, with potential comparable to current antileishmanial agents [203]. Finally, the antiparasitic potential of resveratrol against *L. major* was demonstrated both in vivo and in vitro by [207].

### 3.5. Antiparasitic Activity Against Plasmodium falciparum

*Plasmodium falciparum* is one of the five species of the genus Plasmodium, the causative agent of malaria [241]. Malaria is responsible for about half a million deaths annually, and causes considerable morbidity even in surviving patients [242]. There is an evident need for alternative treatment strategies due to the adverse effects and emerging resistance patterns of current agents [243]. Resveratrol and piceid, along with other derivatives, were found to be effective against Plasmodium falciparum in vitro, following a spectroscopic determination of their structures [208].

### 3.6. Antiparasitic Activity Against Schistosoma mansoni

Schistosomiasis is the helminthic human disease with the highest morbidity and mortality [244,245,246,247], and in spite of increased efforts of vector eradication or infection prevention [248], it remains a significant health problem in certain African regions [249,250] even re-emerging in Southern Europe [251].

The current core treatment for schistosomiasis comprises only praziquantel [249], which is increasingly associated with lower cure rates, potentially associated with emerging resistance to the drug [252,253]. Therefore, the need for novel treatment strategies is urgent. Based on this, Gouveia et al. [209] tested resveratrol and N-acetylcysteine against *S. mansoni*, alone or combined with antihelminthic drugs. Praziquantel and resveratrol and artesunate and resveratrol displayed moderate and good synergies, respectively, and further research on their anti-schistosomal potential seems promising [209].

### 3.7. Antiparasitic Activity Against Toxoplasma gondii

This pathogen causes human toxoplasmosis, a disease affecting a significant percentage of the population but that is usually clinically insignificant; infection usually happens by consuming undercooked meat [254]. Toxoplasmosis is particularly dangerous for pregnant women and for newborns in cases of vertical transmission [255]. In immunocompromised individuals, it may even progress to toxoplasmic encephalitis [256]. The principal treatment agents are dihydropteroate synthetase and dihydrofolate reductase inhibitors [257], but the treatment is ineffective regarding oocysts eradication [254].

Piceatannol was found to be effective against the *T. gondii* both in vitro and in vivo; it proved effective in both cases, in the latter against a tachyzoite stage infection in mice, even though its effectiveness was limited as the mice died by day 15 [211]. A later study found that it was effective in vitro with no cytotoxicity, but in vivo, it just prolonged the mice’s lifespan, and they eventually died [212].

Resveratrol, in combination with sulfamethoxazole-trimethoprim, was found to exert a protective effect on the host organism from the biochemical changes induced by the drugs, while their antiparasitic effectiveness was retained [210]. Resveratrol on its own was identified to exert both direct and indirect anti-tachyzoite effects in the experiments of [213].

### 3.8. Antiparasitic Activity Against Trichinella spiralis

*T. spiralis* is one of the most important foodborne parasites, causing trichinellosis [258]. The disease is contracted from improperly cooked meat, most commonly pork or wild game, and is potentially fatal [259]. The larvae mature in the intestines and disseminate via the bloodstream to encyst within striated muscle tissue, causing a variety of clinical manifestations [260]. Albendazole and mebendazole are current treatments that face limitations in certain population subgroups and may also prove ineffective in severe cases [260]. Resveratrol was found to inhibit different stages of *T. spiralis*, at different concentrations, with higher concentrations being more effective [214].

### 3.9. Antiparasitic Activity Against Trypanosoma cruzi

*Trypanosoma cruzi* is the etiological agent of Chagas disease, a neglected tropical disease, which has worldwide dispersion and global health impact [261]. Chagas disease has a clinically silent acute phase, but about 30% of patients progress to the potentially fatal chronic phase [262]. The current treatment, consisting of nifurtimox and benznidazole, requires chronic administration, carries toxic side effects, and suffers from reduced efficacy [263].

Campo [217] found that resveratrol strongly inhibited parasite replication after 48 h incubation. Resveratrol also selectively altered trypomastigote transcription levels and surface protein expression. One year earlier, the molecular docking of resveratrol in trypanosomal arginine kinase was investigated, and it was found that by inhibiting this enzyme, resveratrol could inhibit the activity of different trypanosomal stages [216]. In the same year, the administration of resveratrol for 30 days in rats, with chronic chagasic cardiomyopathy, was found to exert a cardioprotective and restorative effect, representing a potential starting point for novel treatments [215].

## 4. Antiviral Potential of Stilbenoids Against DNA Viruses

DNA viruses are generally large viruses, which may cause significant morbidity if left untreated [264,265]. While for many of these viruses there exist efficient treatment schemes or vaccines [266,267], the quest for novel therapeutic solutions is important in the context of emerging resistance and potential side effects of antiviral drugs [268,269]. In general, there are ample data documenting the antiviral effects of stilbenoids against DNA viruses (Table 5).

### 4.1. Antiviral Activity Against Hepatitis B Virus (HBV)

This pathogen is transmitted via blood and bodily fluids [289,290]. Infection with the virus is associated with significant mortality all over the world [289,290,291], as it causes liver dysfunction [292], which can even progress to chronic hepatitis [289,291,292], with pregnant women and children accounting for the main risk groups [289,293]. There is a vaccine, as well as several pharmaceutical substances, which are used to avoid or manage the condition, respectively [289,292,293], and have proven successful to a large degree.

Pterostilbene was found to be able to inhibit the activity of the virus’s ribonucleotide reductase in vitro—the IC_50_ being about 0.62 μM—by interacting with protein RRM2 [270]. In addition, it managed to inhibit cell proliferation at an IC_50_ of 20–40 μM in several HCC cell lines, inhibiting DNA synthesis, inducing cell cycle arrest at S phase, and eventually leading to apoptosis. Moreover, it inhibited viral DNA replication in HBV genome-integrated and newly transfected HCC cells [270]. Interestingly, the EC_50_ for inhibiting HBV replication was notably lower than the IC_50_ for inhibiting HCC proliferation [270]. It is worth mentioning that pterostilbene exerted comparable inhibitory effects even in sorafenib and lamivudine-resistant HCC cells [270]. Furthermore, pterostilbene effectively inhibited xenograft growth of HCC in nude mice without causing significant toxicity [270].

Based on the research of Pan et al. [271], 50 μM of resveratrol was sufficient to markedly inhibit viral replication and reduce cytotoxicity in HepG2 and HepG2.2.15 cell lines. This was attributed to inhibition of miR-155 expression and increased autophagy [271]. Resveratrol’s effectiveness against this pathogen extends in vivo, as 100 mg/kg/day was capable of significantly decreasing viral DNA levels in the serum of infected mice [271].

### 4.2. Antiviral Activity Against Cytomegalovirus (CMV)

This virus is an important cause of congenital infections, potentially leading to a multitude of disabilities [294,295], be they intellectual, neurological, developmental, acoustic, or ocular [295]. In the pediatric population, this pathogen is also commonly linked to Ménétrier’s disease [296]. There are cases where the virus displays resistance to pharmacological agents used against it, although the preventive action of letermovir and ganciclovir remains efficacious [297].

Piceatannol exerted inhibitory effects when tested against this pathogen in vitro by preventing lytic changes, reducing viral protein expression and viral DNA replication in a dose-dependent manner, and decreasing SA-β-Gal (senescence-associated β-galactosidase) activity as well as the amount of intracellular ROS; the associated quantity was 10 or 20 μM depending on the extent of the effect [273]. Overall, these findings suggest that piceatannol could be useful in dealing with patients suffering from chronic CMV infection [273].

Pterostilbene can also prevent lytic changes, inhibit viral protein expression, and suppress the replication of viral DNA, bringing about a notable reduction in viral titer in WI-38 cells at an IC_50_ of 1.315 μM [274]. In addition, HCMV infection-caused cell senescence was suppressed due to the presence of pterostilbene, as indicated by the decreased values of the relevant markers. Most worthy of note are the decrease in production of ROS and in the activity of senescence-associated β-galactosidase [274]. Overall, pterostilbene mostly acts after the virus makes its way into the cell via membrane fusion [274].

Resveratrol was also able to inhibit viral replication at an IC_50_ of 1–2 μM when tested on HEL 299 cells, while a minimum 50-fold higher concentration had to be applied before cytotoxic effects were exerted on healthy cells [272]. Notably, viral DNA replication and the late phases of virus-induced phosphatidylinositol-3-kinase signaling and transcription factor activation reached undetectable levels [272]. It was deduced that if applied before four hours post-infection, resveratrol can block virus-induced epidermal growth factor receptor activation, phosphatidylinositol-3-kinase signal transduction, and NF-kappaB and Sp1 transcription factor activation [272]. Thus, the compound probably acts during attachment and entry to host cells [272].

### 4.3. Antiviral Activity Against Epstein–Barr Virus (EBV)

This virus has been linked to various types of malignancy [298,299,300,301]—most notably Burkitt’s lymphoma, autoimmune conditions—namely SLE (systemic lupus erythematosus) [302,303], and very recently, it has even been implicated in the pathogenesis of thyroid conditions such as Graves’ disease and Hashimoto thyroiditis [299].

Resveratrol has proven its antitumoral potential against this pathogen both when tested in vitro and in vivo [276]. When tested in P3HR1 cells, it was able to inhibit the expression of the immediate-early proteins Rta, Zta, and EA-D at an EC_50_ of about 24 μM, without any cytotoxicity being recorded below 55 μM [277]. Inhibition was also noted at the level of the transcriptional activity of the promoters BRLF1 and BZLF1, accompanied by a reduced production of mature viral particles, the EC_50_ for this to occur being 52.2 μM [277]. Similarly, 25 μM of resveratrol in human B cells inhibited viral transformation by more than 70% [280]. At 50 μM, this amount was increased to more than 95% [280]. The inhibition and decreased survivability of the transformed cells were made possible by resveratrol downregulating the anti-apoptotic proteins Mcl-1 and survivin, as a result of suppressing NFκB and STAT-3 signaling pathways, and of reducing the expression of LMP1, an oncogenic viral product [280]. Adding to this, the compound led to reduced expression of miR-155 and miR-34a in infected cells and prevented the expression of BHRF1, an anti-apoptotic viral gene [280]. Comparably, [278] concluded that resveratrol can induce apoptosis in Burkitt’s lymphoma cells by inducing phosphorylation of p38 MAPK and suppressing the ERK1/2 signaling pathway.

Expanding on this, testing conducted on two Burkitt’s lymphoma cell lines led to the realization that resveratrol is a potent inhibitor of the initiation of the lytic cycle [279]. Importantly, resveratrol was able to inhibit the expression of lytic genes, as well as the production of viral particles, in cross-linked Akata cells, a system of Epstein–Barr induction that probably bears a strong resemblance to the in vivo model of the virus’s reactivation [279]. In Raji cells, this property was attributed to its ability to cause ectopic expression of the immediate-early gene BZLF1, while in the case of the Akata cells, to the activation of the B-cell receptor signaling pathway [279]. A decrease in ROS production and in the transcription of factors NF-κΒ and AP1 was also noted [279]. Likewise, when applied to infected Raji cells, resveratrol caused a 57.2% inhibition of early antigen activation at a concentration of 100 times the molar ratio to TPA, while at the maximum tested concentration, early antigen activation was reduced to 8.2 ± 0.4% [276]. During the tests that were conducted on mice with skin papillomas, gross tumor incidence was reduced to 20% after topical application, while 20 weeks post-treatment, tumor incidence was inhibited by more than 30% [276]. These effects can be attributed to a variety of mechanisms, such as resveratrol’s antioxidant and cytotoxic properties [276].

### 4.4. Antiviral Activity Against Herpes simplex Virus (HSV)

The serotypes HHV-1 and HHV-2 have a great range of geographical distribution [304,305], involving facial and/or genital lesions [304,305,306,307]. The spread of the infection to a neonate is possible and a cause for concern [308]. Fortunately, the use of acyclovir has been instrumental in handling these pathologies, while vaccines utilizing nanoparticles are in development [309].

During tests conducted on Vero cells, resveratrol was found to inhibit herpes simplex virus at 50 μg/mL by acting early during its replication cycle, as well as delay the cell cycle at S-G2-M interphase, inhibit reactivation from latently infected neurons, and decrease the amount of the immediate-early regulatory protein ICP-4 [281]. The research of Faith et al. [284] corroborates these findings; at 219 μM, resveratrol was able to inhibit viral replication in Vero cells during their tests by suppressing the activation of NF-κB, and reducing mRNA for ICP0, ICP4, ICP8, as well as the virus’s DNA polymerase. Correspondingly, α-Viniferin, a resveratrol trimer, exhibited antiherpetic activity in vitro at an IC_50_ of 27.4 ± 0.5 μΜ for HSV-1 and 4.2 ± 0.4 μΜ for HSV-2 [285]. Resveratrol was also active on this pathogen when topically applied several times over a few days in infected SKH1 mice as 12.5% and 25% cream [282]. However, for lesion suppression to occur, these creams had to be applied earlier than 12 hours post-infection [283]. Importantly, resveratrol’s effectiveness was the same as that of acyclovir, but it was also active when tested against an HSV-1 acyclovir-resistant strain [283]. Likewise, resveratrol creams applied several times per day in murine lesions, both vaginal and extravaginal, showed variable degrees of effectiveness depending on the formulation, with comparable efficacy limitations being imposed by similar delays in post-infection application [283]. Overall, the 19% resveratrol cream was the most potent [283]. Even though the decrease in morbidity and mortality is indisputable, the details of the mechanism behind it have yet to be elucidated [283].

### 4.5. Antiviral Activity Against Polyomaviruses

SV40, the archetypal virus of this category, has seen extensive use as a model system to broaden our understanding of fundamental eukaryotic cellular processes [310]. There is a link between polyomaviruses and malignancy [310], such as in the case of Merkel cell carcinoma [311]. Other notable pathologies include transplant-related kidney disease, usually caused by BKPyV and JCPyV, and progressive multifocal leukoencephalopathy, a serious condition that is occasionally encountered in immunosuppressed patients and is associated with JC infection [312]. When applied to HL60, a human promyelocytic leukemia cell line, resveratrol managed to notably inhibit cell growth at 25 μM [288]. In addition, at a concentration of 20 μM, viral replication was brought to a halt [288].

### 4.6. Antiviral Activity Against Varicella–Zoster Virus (VZV)

Varicella–Zoster virus is the causative agent for varicella (chickenpox) and herpes zoster (shingles) [313]. Primary infection is followed by lifelong latency with subsequent reactivations, particularly in immunocompromised individuals, and can lead to neuralgia or other neurological sequelae [314,315]. Pathogenesis is characterized by cell-to-cell spread and neurotropism, and the virus possesses the ability to evade the host immune responses [315]. Vaccination, both for the pediatric population and the elderly, has the potential tto limit the pathogen’s impact, but the disease burden remains substantial [316,317]. Resveratrol can inhibit this pathogen’s replication in a dose-dependent manner; at 219 μM, where no toxicity for healthy cells was noted, the synthesis of the immediate-early viral protein IE62 was diminished 72-fold [287]. However, for this effect to be exerted, it had to be administered within 30 hours post-infection [287].

## 5. Antiviral Potential Against RNA Viruses

In general, RNA viruses are associated with a significant burden of disease and represent a significant strain on health systems worldwide. For a number of reasons, certain RNA viruses are associated with persistence [318]. While there have been significant breakthroughs in the elucidation and understanding of the processes involved in related infectious disease pathogenesis and transmission [319], resistance to antiviral agents complicates drug discovery [320]. The stilbenoids discussed herein have been tested against RNA viruses (Table 6) and may prove invaluable in addressing some of the existing or emerging therapeutic challenges.

### 5.1. Antiviral Activity Against SARS-CoV-2

This highly contagious virus belongs to the *Coronaviridae* family and causes coronavirus disease 2019 (COVID-19) [341]. Its spike glycoprotein interacts with the angiotensin-converting enzyme 2 receptor, facilitating viral entry and replication [342]. The clinical manifestations can include severe pneumonia, acute respiratory distress syndrome, and multisystem involvement, more often in immunocompromised and elderly patients [343,344,345].

Pterostilbene inhibits infection of Vero cells by this pathogen, reducing viral particle production by 50% at 19 µM, although it exerted no notable effects on infected Calu-3 cells [321]. Importantly, pterostilbene can inhibit SARS-CoV-2 infection in primary human bronchial epithelial cells cultured under ALI (Air–Liquid Interface) conditions to a great extent [321]. Pterostilbene seems to interfere with viral replication after the pathogen enters the cell, but before the assembly and release of virions take place [321].

Resveratrol also inhibits infection of Vero cells by this pathogen, the reduction in viral particle production reaching 50% at 66 µM, although it also exerted no notable effects on infected Calu-3 cells [321]. Importantly, resveratrol can inhibit SARS-CoV-2 infection in primary human bronchial epithelial cells cultured under ALI (Air–Liquid Interface) conditions to a great extent [321]. Similarly, [322] noticed that resveratrol decreased the viral titer by 80% in MRC5 cells, recording an EC_50_ of 4.6 µM, with cytotoxicity being kept at a minimum. Resveratrol seems to interfere with viral replication after the pathogen enters the cell, but before the assembly and release of virions take place [321]. However, it has poor affinity for Mpro [323]. Piceid can also bind to Mpro, albeit weakly, making it not really effective as an anti-coronavirus agent [323].

### 5.2. Antiviral Activity Against Hepatitis C Virus

Infection with this pathogen can be lethal as it can cause cirrhosis [346] and hepatocellular carcinoma, especially in patients with persistent viraemia [347,348]. While direct-acting antivirals improved cure rates, there are still challenges related to viral resistance, reinfection, and access to treatment [346]. Interestingly, resveratrol was noted to markedly increase the replication of the virus’s RNA and even reverse the effects of ribavirin and interferon against it. Therefore, it cannot be considered a suitable antioxidant therapy in the case of HCV infection. [324].

### 5.3. Antiviral Activity Against Influenza Virus

This highly contagious pathogen mainly affects the respiratory system [72] and is often implicated in pandemics [349]. Due to the resistance it has developed to some of the agents used against it [349,350], as well as its antigenic shift and antigenic drift traits [351], the latter of which necessitates yearly vaccine updates [352], it poses a challenge for the health system.

Piceatannol has potent anti-influenza effects in vitro, but also in vivo [327]. It can inhibit the multiplication of H1N1 and H3N2 viruses, while also preventing infection with H5N1 pseudovirus, with low toxicity. Piceatannol blocks membrane fusion while also possibly acting on the virus’s envelope. Moreover, mice that received piceatannol orally displayed reduced viral titers and notably improved survival rates [327].

Pterostilbene was shown to interact with influenza NS1 protein, inhibiting ubiquitination-mediated degradation of RIG-I, and activating the downstream antiviral pathway, thereby promoting an antiviral state at 200 μM in A549 cells [328]. Pterostilbene’s efficacy was also exhibited in vivo, as it managed to reduce histopathological changes at the level of the lungs in mice [329]. This was made possible due to the activation of the phosphorylated AMP-activated protein kinase alpha/sirtui 1/PPARγ coactivator-1α axis, which counteracted the nuclear factor kappa-B and p38 mitogen-activated protein kinase signaling, which is caused by the virus [329]. In a similar vein, pterostilbene can counteract the virus-mediated M1 macrophage polarization [329]. Finally, it seems to suppress pro-inflammatory mediators and proapoptotic factors [329].

Resveratrol can also be useful against influenza, both in vitro and in vivo [325]. It can cause a 90 ± 2.5% inhibition of viral replication in MDCK cells at 20 mg/mL, which becomes total at 40 mg/mL, by blocking the nuclear-cytoplasmic translocation of viral ribonucleoproteins and decreasing expression of late viral proteins, probably through inhibition of the activity of protein kinase C and its dependent pathways [325]. When it comes to infected mice, there was a notable increase in survival and a notable decrease in pulmonary viral titers [325]. Importantly, there were no toxic effects in either case [325]. The fact that resveratrol’s action is exerted on a cellular function, and not a viral one, presents positive implications regarding its effectiveness as a drug [325]. Finally, piceid was found to be potent against the virus’s NA by docking into the active site of N1 during testing in silico [326].

### 5.4. Antiviral Activity Against Human Enteroviruses (HEVs)

The enterovirus genus comprises several viral pathogens, most notably enterovirus 71, enterovirus D68, and Coxsackie virus [353]. HEVs are responsible for a variety of manifestations, ranging from self-limited illnesses such as hand, foot, and mouth disease, or upper respiratory infections, up to encephalitis, myocarditis, meningitis, or acute flaccid paralysis [354,355,356,357].

Pterostilbene is potent against enteroviruses both in vitro and in vivo [331]. On one hand, it is capable of inhibiting viral attachment to a great degree, inactivating viral particles, blocking viral binding to the receptors, and increasing virion stability [331]. By occupying a hydrophobic pocket in viral protein 1, owing to its strong binding affinity, it acts as a potent inhibitor [331]. On the other hand, oral intake of pterostilbene ameliorated infection-related symptoms and reduced mortality in hSCARB2 transgenic mice [331].

Resveratrol is highly effective against enterovirus 71 due to its ability to inhibit the synthesis of VP1, a capsid protein, while also having an anti-inflammatory role by preventing the phosphorylation of IKKα, IKKβ, IKKγ, IKBα, NF-κB p50, and NF-κB p65 [330]. In addition, the marked upregulation of IL-6 and TNF-α in EV71-infected rhabdosarcoma cells was blocked by the compound. These effects were exerted at 30 μg/mL of resveratrol [330].

### 5.5. Antiviral Activity Against Human Rhinovirus

This virus is commonly implicated in cases of acute viral respiratory infections [358,359,360], but it can also negatively affect the progression of chronic pulmonary conditions such as asthma and COPD [358]. The resulting morbidity can be significant, with children occasionally requiring admission to the ICU and older adults requiring oxygen support [359].

Application of 300 μM of resveratrol on H1HeLa cell monolayers and ex vivo nasal epithelia resulted in potent RSV-16 replication inhibition, as well as diminished secretion of IL-6, IL-8, and RANTES, leading to a less pronounced inflammatory response, without any display of cytotoxicity [332]. Furthermore, the upregulated expression of ICAM-1 by the virus was counteracted by resveratrol [332].

### 5.6. Antiviral Activity Against Theiler’s Murine Encephalomyelitis Virus (TMEV)

This virus determines encephalomyelitis and chronic demyelinating disease in rodents [361,362]. Since the demyelination it causes resembles that of multiple sclerosis, it is used as a viral model in the study of that pathology [362]. Interestingly, resveratrol was found to exacerbate the demyelination caused by the pathogen when tested in vivo, with the inflammation having a notably increased intensity [333].

### 5.7. Antiviral Activity Against Human Metapneumovirus

This pathogen causes symptoms such as cough, sore throat, and fever [363,364], but it can also cause acute respiratory distress syndrome, especially in children, the elderly, and the immunocompromised [364,365]. Regrettably, its role is still largely underestimated [363].

When resveratrol was applied to airway epithelial cells, cellular oxidative damage was reduced [334]. Moreover, the production of inflammatory mediators, as well as their viral replication, was decreased. The anti-inflammatory effects were exerted by inhibition of NF-κB and IRF-3 [334]. Since viral gene transcription and protein synthesis remained intact, the inhibition of viral replication is considered to have taken place during viral assembly, release, or both [334].

### 5.8. Antiviral Activity Against Respiratory Syncytial Virus (RSV)

RSV is a leading cause of acute lower respiratory tract infections worldwide, affecting primarily infants and young children [366]. Fusion and attachment glycoproteins mediate viral entry and spread [367]. With limited therapeutic options, RSV remains a major target for novel antiviral compounds despite recent advances in vaccines and monoclonal antibodies [368].

At 50 μM, resveratrol decreased the level of IL-6 in infected 9HTEo cells, although not that of IL-8, resulting in a milder inflammatory response [336]. This was associated with suppression of the activation of NF-κB by TRIF and TBK1, and of the expression of RIP-1, both of which the pathogen normally upregulates [336]. At an optimal concentration of 100 μM, the compound was also able to reduce viral titers in both 9HTEo cells and Hep-2 cells [336]. When administered to BALB/c mice at 30 mg/kg/day, resveratrol was shown to notably reduce interferon-γ production, although not any of the other associated cytokines, thereby decreasing the levels of airway inflammation; the amount of lymphocyte-rich infiltrates and tissue damage was markedly diminished [335]. These findings are in accordance with those of Liu et al. [369], who also noticed diminished inflammation intensity in BALB/c mice due to reduced interferon-γ levels, as a result of SARM upregulation and TRIF downregulation. The same is true of the tests that were carried out by Long et al. [338]; both BALB/c mice and nude mice benefited from anti-inflammatory effects following resveratrol administration due to MMP-12 downregulation, with the former also experiencing a decrease in interferon-γ production. In a similar vein, resveratrol seems to reduce the levels of NGF, thereby suppressing persistent airway inflammation in mice [339]. Interestingly, when administered to SD mice at 30 mg/kg/day via retention enema, resveratrol was shown to boost the inflammatory response against the pathogen by upregulating TNF-α, IL-2, IFN-γ, and SIgA [337].

### 5.9. Antiviral Activity Against Human Immunodeficiency Virus (HIV)

HIV is the causative agent of acquired immune deficiency syndrome (AIDS), and primarily targets CD4^+^ T cells, macrophages, and dendritic cells, leading to progressive immune dysfunction and higher susceptibility to infections or malignancies [370,371]. Antiretroviral therapy has seen major advances, but drug resistance, viral latency, and lack of a curative strategy still motivate the search for novel antiviral compounds [372,373].

Resveratrol was found to inhibit HIV-1 strains that carry the M184V mutation in reverse transcriptase by interfering with DNA synthesis when tested in peripheral blood lymphocytes, the associated EC_50_ being 5.8 μM, although it was not active on the wild type of the virus [340].

## 6. Discussion

**Figure 2:**
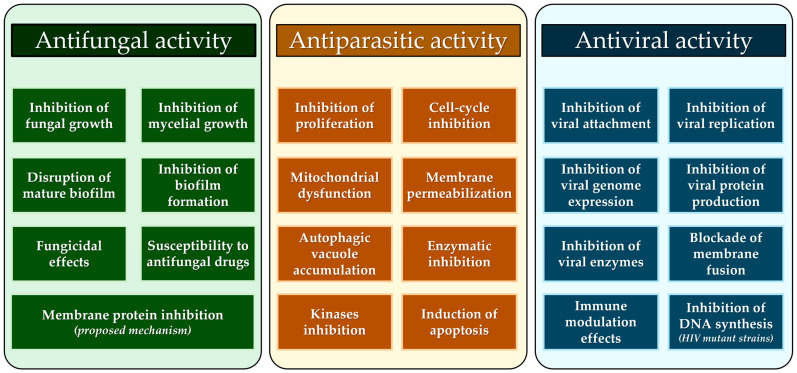
Synthetic overview of the action mechanisms of stilbenoids against fungi, parasites, and viruses.

Stilbenes have a host of beneficial health properties, including antioxidant, anti-inflammatory, and anticancer activity [374,375,376], while analgesic and anti-atherosclerotic properties were also demonstrated [374,375]. We have shown above the extent of their antifungal, antiviral, and antiparasitic roles in humans, and a summary of the mechanisms of action is provided in Figure 2 and reviewed in the following sections.

### 6.1. Overview of the Antifungal Properties of Stilbenoids

Based on the results of the research presented in the text, stilbene phytoalexins in most cases efficiently affect fungal growth, or have an otherwise notable antifungal action (Table 7).

In the majority of antifungal applications, resveratrol has been the most applied stilbenoid, especially in vitro. Older studies identified the antifungal potential of resveratrol [378,379,380,381] and recognized that it, along with other phytoalexins, forms part of the natural defense mechanism of grapevines against fungal infection [382], and against Botrytis cinerea, a notable necrophytic fungus of economic importance in viticulture and horticulture in general [383,384]. There is a corpus of research that directly contradicts the potential of resveratrol against *C. albicans*, namely that of Docherty et al. [385] and Weber et al. [166].

A particular case concerns the integration of a resveratrol-synthase gene from *Polygonum cuspidatum* to *Arabidopsis thaliana*, using parts of a viral vector. The new transgenic plants accumulated trans-piceid, which inhibited *Colletotrichum higginsianum* colonization [386]. This hemibiotrophic fungus causes anthracnose disease on many cruciferous plants [387]; due to its hemibiotrophic nature, it has developed a range of specialized invasion structures and, as it is a main pathogen of the plant *Arabidopsis thaliana*, it is one of the best studied fungi of its genus [388].

*Candida auris* is a novel fungal pathogen that invasively colonizes the skin and thrives on inert surfaces (steel, plastics, etc.), resulting in rapid nosocomial transmission; *C. auris* has resistance mechanisms to all antifungal classes (azoles, echinocandin, polyene, nucleoside analogues) [389,390]. The biofilm-forming *C. auris* also has a worrisome ability to survive on surfaces after common hospital disinfectant procedures (sodium hypochlorite, quaternary ammonium, or peracetic acid-based disinfectants) [389,391].

Since biofilm-forming *C. auris* shares some genetic mechanisms of resistance to antimicrobials with *C. albicans* [389,391], we propose that an interesting future research avenue would be the quantification of stilbenes’ effects on the *C. auris* pathogenicity. Currently, there is a lack of experimental data on this issue [392].

### 6.2. Overview of the Antiparasitic Activity of Stilbenoids

In general, as presented herein, there have been a host of experiments regarding the antiparasitic potential of stilbenoids (Table 8). Resveratrol analogues and derivatives have been tried as antiparasitic agents with different degrees of success [203]. Resveratrol is effective against *Echinococcus* spp. in vitro, via a variety of mechanisms [198], and it can reduce worm burden in vivo in *H. diminuta* infections [199]. Against different *Leishmania* species, it can inhibit proliferation and cause incidental parasite death [201], inhibit trypanothione reductase [204], and promote the death of host cells [206]. Its analogues can disrupt mitochondrial function and lead to membrane permeabilization, and promote autophagic vacuole accumulation [203].

Along with piceid, resveratrol is effective against *P. falciparum*, although the precise mechanism has not been elucidated [208]; in the same manner, the mechanism of action of resveratrol against *T. spiralis* is unknown [214]. In the case of *T. gondii*, resveratrol induces mitochondrial dysfunction, inhibits proliferation, and promotes apoptosis both in infected and host cells [210,211,212,213]. It also exhibits synergistic action with antiparasitic drugs used against *S. mansoni* [209], and finally, against *T. cruzi*, resveratrol [215,216,217].

Apart from its direct antiparasitic effects, resveratrol was found to have a protective effect in *S. japonicum*-induced liver cirrhosis in mice, where it inhibited VEGF expression and reduced oxidative stress [393]. Other research has revealed the effectiveness of resveratrol in parasites that are non-pathogenic to humans, such as *Philasterides dicentrarchi* [394], but which nonetheless pose a threat to the flora of marine environments [395]. Similarly, resveratrol was found to be effective in inhibiting the energy metabolism of *Raillietina echinobothrida*, a fowl tapeworm [396]. Moreover, resveratrol was found to be potentially able to inhibit helminth infection-associated carcinogenesis, when combined with other drugs, in individuals infected with *Schistosoma haematobium* and *Opisthorchis viverrine*; some metabolites of those parasites are believed to be carcinogenic [397].

Generally, piceatannol is a naturally occurring protein-tyrosine kinase inhibitor [398] which has the potential, either alone or following chemical manipulation, to be of use as an antiparasitic agent against *L. amazonensis* and *L. braziliensis* [399]. Its activity against this enzyme was observed in both chloroquine-sensitive and chloroquine-insensitive strains [400]. As such, it may be possible to use it as a substitute for chloroquine, which has the same mode of action, in strains showing resistance, therefore hindering the maturation of the malaria parasite. Early research results had investigated the potential of using piceatannol to inhibit the asexual maturation of *P. falciparum* [401].

The antileishmanial activity of piceatannol is more or less based on similar mechanisms as that of resveratrol, while pterostilbene exerts cytotoxicity against host cells [202]; other researchers have noted that the antileishmanial potential of piceatannol is associated with its ability to inhibit the parasite’s protein kinase. Piceatannol is also effective against *T. gondii*, in a manner similar to resveratrol [211,212]. Finally, piceid was recently found to be able to inhibit the S-adenosyl-L-homocysteine hydrolase of *N. fowleri* [200].

**Table 8:** Synopsis of the antiparasitic mechanisms of the stilbenoids discussed in the text, organized by parasite.

Parasite	Disease	Vectors	Endemic Areas	Mechanisms of Action	References
*Echinococcus* spp.	Echinococcosis	Dogs and other canids	Worldwide distribution	Reduction in viability, ultrastructural damage, and increased autophagy gene transcription	[198,218,402]
*H. diminuta*	Hymenolepiasis	Rats (and other mammals)	Rare (isolated cases)	Reduction in worm burden	[199,403]
*N. fowleri*	Primary amoebic meningoencephalitis	None	Bodies of warm freshwater	Enzymatic inhibition	[200,404]
*Leishmania* spp.	Leishmaniasis	Phlebotomous and Lutzomyia sandflies	Africa, Central and South Asia, Central and South America	Inhibition of proliferation and cell cycle progression, incidental parasite death, mitochondrial function disruption, membrane permeabilization, autophagic vacuole accumulation, inhibition of trypanothione reductase, promotion of host cell death, protein kinase inhibition	[84,201,202,203,204,206,405,406,407]
*P. falciparum*	Malaria	Anopheles mosquitoes	South America, Africa, India, and South Pacific Islands	Unknown mechanism	[208,408,409]
*S. mansoni*	Schistosomiasis (bilharziasis)	Biomphalaria freshwater snails	Sub-Saharan Africa, South America, Eastern Mediterranean	Synergistic action with antiparasitic drugs	[209,410]
*T. gondii*	Toxoplasmosis	Cats	Worldwide	Inhibition of parasite proliferation, induction of mitochondrial dysfunction, protective effect for host organism, induction of apoptosis	[210,211,212,213,411]
*T. spiralis*	Trichinellosis	None specific (undercooked meat)	Worldwide	Unknown mechanism	[214,412]
*T. cruzi*	Chagas disease	Triatominae insects	Rural areas of South America	Reduction in parasitaemia, cardioprotective effect, arginine kinase inhibition, cell cycle inhibition	[215,216,217,413]

### 6.3. Overview of the Antiviral Activity of Stilbenoids

Regarding the antiviral effects of stilbenoids discussed in the text, for most of the viruses against which they were tested, they inhibited their replication (Table 9). This is true for all Baltimore Group I viruses, i.e., ASFV, CMV, DEV, EBV, HSV, polyomaviruses, PRV, and VZV. Other molecular mechanisms were also inhibited in certain cases, such as the expression of viral proteins and virus-associated cell signaling. In regard to viral replication, its inhibition has been documented in all tested cases for the other groups, with the exception of HCV and TMEV. For a number of viruses, namely HSV, PRV, human enterovirus, human rhinovirus, HBV, influenza, and RSV, antiviral effects have been recorded in vivo.

We would also like to note that several of the stilbenoids discussed in the text have demonstrated a potent inhibitory activity against HIV-1 integrase and eukaryotic MOS1 transposase [414].

**Table 9:** Synopsis of the antiviral mechanisms of the stilbenoids discussed in the text, organized by virus; viruses are grouped based on the Baltimore classification [415].

Baltimore Group		Virus	Effect	References
Group I	Double-stranded DNA viruses (dsDNA)	CMV	Inhibition of viral replication; inhibition of virus-associated cellular signaling; suppression of mechanisms of cellular senescence; inhibition of cell lysis; inhibition of viral protein expression	[272,273,274]
		EBV	Inhibition of early antigen activation; free radical scavenging; reduction in virion production; cell cycle arrest and apoptosis in infected cells; inhibition of viral protein expression; anti-apoptotic effect in infected cells	[276,277,278,279,280]
		HSV	Inhibition of viral replication and reactivation; suppression of lesion formation (in vivo); reduction in viral replication (in vivo); inhibition of viral genome expression	[281,282,283,284]
		Polyomaviruses	Inhibition of viral DNA synthesis	[288]
		VZV	Inhibition of viral replication	[287]
Group IV	Positive-sense single-stranded RNA viruses (+ssRNA)	HCV	No significant antiviral effects	[324]
		Human enterovirus	Inhibition of viral replication; promotion of cytokine secretion; inhibition of viral replication; inhibition of viral attachment; inhibition of viral protein production; increase in survival rate (in vivo)	[330,331]
		Human rhinovirus	Inhibition of viral replication; suppression of inflammation	[332]
		SARS-CoV2	Inhibition of viral replication inside infected cells; weal Mpro inhibition	[321,322,323]
		TMEV	No antiviral effect	[333]
Group V	Negative-sense single-stranded RNA viruses (–ssRNA)	Human metapneumovirus	Inhibition of viral replication; reduction in inflammation; reduction in oxidative damage	[334]
		Influenza virus	Inhibition of viral replication inside infected cells; inhibition of viral neuraminidase; inactivation of viral particles; blockade of membrane fusion; reduction in viral titers (& promotion of survival in vivo); immune modulation	[325,326,327,328,329]
		RSV	Reduction in viral titers (in vivo); increase in pro-inflammatory cytokine secretion (in vivo); decrease in interferon and MMP production (in vivo); decrease in interleukin production; inhibition of viral replication; modulation of inflammation (in vivo)	[335,336,337,338,339]
Group VI	Single-stranded RNA viruses with a DNA intermediate in their life cycle (ssRNA-RT)	HIV	Inhibition of viral replication	[340]
Group VII	Double-stranded DNA viruses with an RNA intermediate in their life cycle (dsDNA-RT)	HBV	Inhibition of viral replication; inhibition of infected cell proliferation; reduction in cytotoxicity in infected cells; decrease in serum DNA in mice (in vivo)	[270,271]

### 6.4. Toxicity of Stilbenoids

The toxicity of stilbenoids is of interest both regarding their pharmacological applications and also concerning their use as food supplements. In most studies, oral administration of resveratrol has not been associated with toxicity-related side effects or adverse effects [416]; in some cases, mild side effects have been reported [417], and the intensity of (mostly gastrointestinal) effects increases at repeated doses of over 2.5 g [416]. Single doses, even this high, do not seem to be causing any problems during testing [418,419], even though the interaction of stilbenoids with other drugs has not been researched [420].

Specifically on the stilbenoids mentioned in this paper, in their antibacterial, antifungal, antiviral, or antiparasitic capacity, from the vastness of research results mentioned, few efforts have focused on the toxicity of the compounds used.

For pterostilbene, little to no toxicity was reported [148,150].

In the case of piceatannol, toxicity remains relatively high, and this also explains its anticancer potential. For example, in the study of Duarte et al. [84], cytotoxicity against RAW macrophages was achieved at 5.7 μg/mL, while for the reference drug it was >25 μg/mL. In another study, however, against peritoneal macrophages, the toxicity limit of piceatannol and pterostilbene was found to be much higher than the active concentrations [202]. Yet other results report that there is no in vitro toxicity at effective concentrations, and very limited toxicity in test animals [211,212]. On the other hand, cytotoxicity against infected cells was high in the research of Wang et al. [273], but this was the intended effect against infected cells.

For resveratrol, even though it may have antileishmanial potential, its use in clinical practise may be hindered by its cytotoxicity against both infected and non-infected cells, in particular cell lines [206]. In another study [204], resveratrol was again found to be effective against *L. braziliensis*, but at a concentration also potentially toxic to healthy cells. Other experiments, where resveratrol was combined with amphotericin B, did not find any notable cytotoxicity at the concentrations against parasites [201,207]. Experiments on its antifungal potential again noted no significant toxicity against certain cell types, in the concentrations used [145,152].

In studies on its antiviral potential, resveratrol was found to be less cytotoxic than lopinavir, ritonavir, and chloroquine [322] and also to be mildly cytotoxic at the maximum concentration needed to effect maximum inhibition of viral replication in infected cells [325]. Against EV71, the CC_50_ of resveratrol was far less than that of ribavirin and more than the corresponding IC_50_ [330]; the same is the case for the antiviral action of resveratrol against CMV [272] and against DEV [275]. Against human rhinovirus and VZV, the maximum concentration used was not toxic [287,332].

### 6.5. Bioavailability of Stilbenoids

In general, compared to other more well-studied compounds, there is a relative lack of data on the pharmacokinetic properties of stilbenoids [421]; because the class is made up of different compounds, each with its own particularities, it is difficult to reach a generalized conclusion [421,422]. Like most compounds, an important regulator of bioavailability is their structure. When ingested, a part of their metabolization takes place in the liver and intestines [421,422]. At the level of the liver, either glucuronidation or sulfation takes place—the latter is believed to be associated with the cardioprotective and anticancer effect [423]. As presented in the introduction, stilbenoids have different water solubilities. Amongst the aglycones, piceatannol is the most soluble one, with pinosylvin being practically insoluble; the presence of hydroxyl groups increases the tendency of the compounds to dissolve in the aqueous phase. For drug compounds, it is generally accepted that higher water solubility values are desired [424]; more lipophilic compounds will not generally be absorbed in the GI tract and may even lead to compound precipitation and toxicity [425]. Compound stability inside the GI tract is also influenced by structure; in general, a balance between hydrophilicity and hydrophobicity is required [426]. Glycosides are generally more water-soluble, and glycosylation is generally associated with better pharmacokinetic parameters [427].

While it is a potent antioxidant and antimicrobial agent, resveratrol, being highly water-soluble compared to other stilbenoids, is rapidly destroyed in the stomach due to stomach acids [428]. Its oral bioavailability has been estimated to be less than 1% in a rat model, although in humans, it has been reported to reach as high as 75% [429]. Piceid, on the other hand, has been found to have a good GIT absorption [200], and pterostilbene appears to have better oral bioavailability than resveratrol, in rat models [430].

Analogues of resveratrol, which may even have a higher efficiency in inhibiting certain metabolic pathways, are also apparently better absorbed via the gastrointestinal tract [204]. Piceatannol is considered more metabolically stable than its precursor molecule, resveratrol, even if it may require structural modifications for its bioactivity to be enhanced in certain pathways [78]. Collectively, the systematic use of stilbenoids in medicine is hampered by their poor oral bioavailability and water solubility, their high rate of metabolic breakdown, and relatively non-specific nature in targeting cellular and tissue targets [420]. Research is underway to find alternatives that circumvent these shortcomings [431,432].

Therefore, it is apparent that some solutions on matters of bioavailability may be required; these can be provided by novel systems, which are currently under development, such as liposomes, micelles, micro-emulsions and nano-emulsions, colloidal capsules, and solid nanoparticles. All these may be viable carriers for the stilbenoids presented herein [433,434,435]. Another avenue, specifically addressing those cases where there is a risk of surgical site infections [436,437,438,439,440,441], would be the incorporation of stilbenoids in 3D-printed biomaterials [442,443,444,445,446].

### 6.6. Recommendations for Further Research

Given the anticancer effects of several different stilbenoids, it should be explored how they can be used, most probably as adjuvant therapies, along with more traditional radiotherapy or chemotherapy; one example would be the combination with somatostatin analogues in cases of hepatocellular carcinoma [447,448,449,450,451].

As the stilbene core is a very versatile and useful chemical structure [452]—indeed, raloxifene and tamoxifen, two stilbene-based drugs, are already commercially available [453]—further development along this axis could result in other viable pharmacotherapeutic solutions. The development of synthetic analogues, with longer half-lives and better bioavailability, will also potentially address the current limitations of biological stilbenes [454]. Regarding stilbene extraction, apart from the need for developing more environmentally friendly and efficient methods for extraction, the use of industrial and agricultural byproducts is gaining traction as another source of stilbenes [455,456,457,458]. Further research on the ethnomedical and ethnopharmacological traditions of different cultures will also potentially reveal more plant-derived stilbenoid sources and a wider spectrum of applications.

Lastly, we would like to note that there is a potential for the use of stilbenoids or their analogues in the agricultural sector to promote plant resistance to infectious agents [459]. This will help reduce the use of pesticides and fertilizers, which have a negative environmental impact [460,461,462,463]. Some stilbenoid-producing plants are also under research for their larvicide potential [464,465,466], which are also dangerous for the environment [467].

Some stilbenoid-producing plants have also been under research in attempts at phytoremediation of mining sites and water purification [468,469,470] and they can be a novel and effective solution to combat heavy metal pollution in mining and industrial sites [471,472,473,474,475,476,477,478]—in all these applications, stilbenoids probably form an integral part of the effectiveness of these plants and it should be explored how much of this activity is attributable to stilbenoids themselves.

## 7. Conclusions

Stilbenoid phytoalexins, comprising astringin, piceid, piceatannol, pterostilbene, pinosylvin, and resveratrol, have a host of beneficial health properties. These include antifungal, antiparasitic, and antiviral properties that are exerted via inhibition of replication of the pathogenic organisms, interference in their metabolic mechanisms, or cytoprotective effects. Of note, a few studies have been conducted in vivo in test animals, and protection against infection has been documented, especially in the cases of viruses. Further studies should focus on questions of bioavailability, which may be resolved using novel delivery techniques. Finally, future research should focus on further documenting the mechanisms of action and examining their potential against other pathogens.

## Figures and Tables

**Table 2:** Availability in nature of the most frequent stilbenoids; the plants are organized alphabetically by family, genus, and species—regarding resveratrol, only the most important plant species are mentioned.

Compound	Family	Genus	Species	References
Astringin	Myrtales	*Myrtaceae*	*Ε. astringens*	[80]
	Pinaceae	*Picea*	*P. abies* L.	[81]
			*P. jezoensis*	[82]
			*P. sitchensis*	[83]
Piceatannol	Euphorbiaceae	*Euphorbia*	*E. lagascae*	[84]
	Fabaceae	*Arachis*	*A. hypogaea*	[85]
		*Cassia*	*C. abbreviata*	[86]
		*Vouacapoua*	*V. americana*	[77]
	Musaceae	*Ensete*	* E. glaucum *	[87]
		*Musa*	* M. acuminata *	
			* M. itinerans *	
	Myrtaceae	*Rhodomyrtus*	* R. tomentosa *	[88]
	Passifloraceae	*Passiflora*	*P. caerulea*	[77,89]
			*P. edulis*	
			*P. longifilamentosa*	
	Pinaceae	*Picea*	*P. jezoensis*	[82]
	Polygonaceae	*Rheum*	*R. rhaponticum* L.	[90]
	Vitaceae	*Vitis*	*V. rotundifolia* Michx.	[91]
			*V. vinifera*	[92,93]
Piceid	Pinaceae	*Picea*	*P. jezoensis*	[82]
	Polygonaceae	*Polygonum*	*P. cuspidatum*	[94]
	Vitaceae	*Vitis*	*V. vinifera*	[95,96]
Pinosylvin	Fabaceae	*Arachis*	*A. hypogaea*	[97]
		*Cajanus*	*C. cajan*	[98]
	Rhamnaceae	*Hovenia*	*H. dulcis* Thunb.	[99]
	Lauraceae	*Lindera*	*L. reflexa* Hemsl	[100]
	Myrtaceae	*Agonis*	*A. flexuosa*	[101]
	Pinaceae	*Picea*	*P. abies*	[102]
			*P. glauca*	[103]
		*Pinus*	*P. banksiana*	[104,105]
			*P. brutia* Hen.	[106]
			*P. caribaea*	[107]
			*P. cembra*	[108]
			*P. contorta*	[108]
			*P. densiflora*	[109]
			*P. halepensis* Mill.	[110]
			*P. merkusii*	[111]
			*P. nigra Arn.*	[112]
			*P. palustris*	[113]
			*P. pinaster*	[114]
			*P. resinosa*	[105]
			*P. roxburghii* Sargent	[115]
			*P. strobus*	[113]
			*P. sibirica*	[108]
			*P. sylvestris*	[116]
			*P. taeda*	[117]
	Stemonaceae	*Stemona*	*S.* cf. *peirrei*	[118]
			*S. collinsae*	[119]
			*S. tuberosa*	[120]
Pterostilbene	Ericaceae	Vaccinium	*V. ashei*	[121]
			*V. virgatum*	[122]
	Fabaceae	*Pterocarpus*	*P. marsupium*	[122]
			*P. santalinus*	[122]
	Vitaceae	*Vitis*	*V. amurensis*	[123]
			*V. vinifera*	[122,124]
Resveratrol	Moraceae	*Artocarpus* J. R. et G. Forst.	*A. lacucha (Roxb.)* Buch.	[93]
		*Cudrania* Trec.	*C. cochinchinensis* Lour.	[93]
		*Morus* L.	*M. alba* L.	[93]
			*M. macroura* Miq.	
			*M. nigra* L.	
	Polygonaceae	*Polygonum*	*P. cuspidatum*	[94,125]
			*P. grandiflorum*	[57]
		*Rheum*	*R. rhaponticum* L.	[90]
	Myrtaceae	*Eucalyptus* L. Herit	*E. tereticornis* Smith	[93]
	Ranunculaceae	*Paeonia* L.	*P. suffruticosa* Andr.	[93]
			*P. suffruticosa* Andr. var. *papaveracea* *(Andr.) Kerner*	
	Vitaceae	*Ampelopsis* Michaux	*A. cantoniensis* (Hook. & Arn.) K. Koch	[93]
			*A. japonica* (Thunb.) Makino	
		*Cissus* L.	*C. quadrangularis* L.	[93]
			*C. sicyoides* L.	
		*Parthenocissus* Planch.	*P. tricuspidata* (S. et Z.)	[93]
			*P. quinquefolia* (L.) Planch.	
		*Tetrastigma* Planch.	*T. hypoglaucum* Planch ex Franch.	[93]
			*T. serrulatum* (Roxb.) Planch	
		*Vitis*	*V. amurensis*	[63]
			*V. vinifera*	[93]

**Table 3:** Available data on the antifungal potential of the stilbenoids discussed in the text, organized alphabetically by genus and species, and then chronologically (potency reported as per the original research).

Genus	Species	Study Type	Compound	Stilbenoid Origin	Concentration	Effect (Mechanism)	Reference
*Aspergillus*	*A. flavus*	In vitro	Pterostilbene	Pure compound	15.94 μg/mL (EC_50_)	Inhibition of mycelial growth and of aflatoxin production	[143]
	*A. fumigatus*	In vitro	Pinosylvin	*Pinus* spp.	106.12 μg/mL and 10,612 μg/mL (concentrations used)	Inhibition of germination and growth	[144]
*Candida*	*C. albicans*	In vitro	Pinosylvin	Pure compound	62.5 μg/mL (MIC)	Inhibition of growth	[145]
		In vitro	Resveratrol	Pure compound	20 μg/mL (MIC)	Fungicidal effect	[146]
		In vitro	Pinosylvin	*Pinus* spp.	106.12 μg/mL and 10.612 μg/mL (concentrations used)	Inhibition of growth	[144]
		In vitro	Astringin	Pure compound	>81.20 μg/mL (MIC_80_)	No appreciable growth inhibition at tested concentrations	[147]
			Pinosylvin	Pure compound	≥42.45 μg/mL (MIC_80_)	No appreciable growth inhibition at tested concentrations	
			Pterostilbene	Pure compound	25.60 μg/mL (MIC_80_)	Inhibition of growth	
			Resveratrol	Pure compound	>45.65 μg/mL (MIC_80_)	No appreciable growth inhibition at tested concentrations	
		In vitro	Pterostilbene	Pure compound	32 μg/mL (MIC_80_)	Inhibition of biofilm formation	[148]
		In vivo			32 & 64 μg/mL (concentrations preventing biofilm formation in vivo)		
		In vitro	Resveratrol (only analogues investigated)	Pure compound	n/a (only analogues investigated)	Fungicidal effect	[149]
		In vitro	Pterostilbene	Pure compound	16 & 128 μg/mL (action against biofilm formation & mature biofilm, respectively)	Inhibition of biofilm formation and action against mature biofilm	[121]
		In vitro	Pterostilbene	Pure compound	5 & 10 μg/L (MIC_50_ & MIC_80_ respectively)	Inhibition of biofilm formation and disruption of existing biofilm	[150]
			Resveratrol	Pure compound	160 & 175 μg/L (MIC_50_ & MIC_80_ respectively)		
		In vitro	Resveratrol	Pure compound	1200 & 3200 μg/mL (cis and trans form, respectively)	Promotion of susceptibility to azole antifungal agents	[151]
*Cladosporium*	*C. herbarum*	In vitro	Pinosylvin	*S. collinsae*	n/a (only structurally similar stilbenoids were tested)	Inhibition of growth	[119]
		In vitro	Pinosylvin	*S.* cf. *pierrei*	10 μg/mL (EC_50_)	Inhibition of growth	[118]
*Epidermophyton*	*E. floccosum*	In vitro	Resveratrol	Pure compound	25–50 μg/mL (IC_75_)	Fungicidal effect (precise mechanism unknown; probably some type of inhibition of membrane protein functionality)	[152]
*Microsporum*	*M. gypseum*	In vitro	Resveratrol	Pure compound	25–50 μg/mL (IC_75_)	Fungicidal effect (precise mechanism unknown; probably some type of inhibition of membrane protein functionality)	[152]
*Trichophyton*	*T. mentagro-phytes*	In vitro	Resveratrol	Pure compound	25–50 μg/mL (IC_75_)	Fungicidal effect (precise mechanism unknown; probably some type of inhibition of membrane protein functionality)	[152]
	*T. tonsurans*	In vitro	Resveratrol	Pure compound	25–50 μg/mL (IC_75_)	Fungicidal effect (precise mechanism unknown; probably some type of inhibition of membrane protein functionality)	[152]
	*T. rubrum*	In vitro	Resveratrol	Pure compound	25–50 μg/mL (IC_75_)	Fungicidal effect (precise mechanism unknown; probably some type of inhibition of membrane protein functionality)	[152]
		In vitro	Astringin	Pure compound	>81.20 μg/mL (MIC_80_)	No appreciable growth inhibition at tested concentrations	[147]
			Pinosylvin	Pure compound	21.22 μg/mL (MIC_80_)	Inhibition of growth	
			Pterostilbene	Pure compound	6.40 μg/mL (MIC_80_)	Inhibition of growth	
			Resveratrol	Pure compound	>45.65 μg/mL (MIC_80_)	No appreciable growth inhibition at tested concentrations	
*Trichosporon*	*T. beigelii*	In vitro	Resveratrol	Pure compound	10 μg/mL (MIC)	Fungicidal effect	[146]
	*T. cutaneum*	In vitro	Resveratrol	Pure compound	80 μg/mL (MIC_80_)	Inhibition of biofilm formation and disruption of existing biofilm	[153]

**Table 4:** Available data on the antiparasitic potential of the stilbenoids discussed in the text, organized alphabetically by genus and species, and then chronologically (potency reported as per the original research).

Genus	Species	Study Type	Compound	Extract from	Concentration	Mechanism	Year	Reference
*Echinococcus*	*E. granulosus*	In vitro	Resveratrol	Pure compound	2.28, 11.41, and 22.82 μg/mL (concentrations used)	Reduction in viability, ultrastructural damage, and increased autophagy gene transcription	2023	[198]
		In vivo			100 mg/kg per day (for 14 d)	n/a (ineffective)		
	*E. multilocularis*	In vitro	Resveratrol	Pure compound	2.28, 11.41, and 22.82 μg/mL (concentrations used)	Reduction in viability, ultrastructural damage, and increased autophagy gene transcription	2023	[198]
		In vivo			100 mg/kg per day ^1^ (for 14 d)	n/a (ineffective)		
*Hymenolepis*	*H. diminuta*	In vivo	Resveratrol (alone and in extract form)	*Carex baccans* Nees roots	Various doses for the whole extract and for resveratrol separately	Reduction in worm burden	2015	[199]
*Naegleria*	*N. fowleri*	In silico	Piceid	*Gossypium hirsutum*	n/a (molecular docking study)	S-adenosyl-L-homocysteine hydrolase inhibition	2025	[200]
*Leishmania*	*L. amazonensis*	In vitro	Resveratrol (with amphotericin B)	Pure compound	9.59 μg/mL (IC_50_ − amastigotes) and 6.16 μg/mL (IC_50_ − promastigotes)	Inhibition of proliferation and incidental parasite death	2014	[201]
		In vivo	Piceatannol	Pure compound	11 μg/mL (amastigotes) and 15.88 μg/mL (IC_50_ − promastigotes)	Alteration of mitochondrial membrane potential	2015	[202]
			Pterostilbene		8.51 μg/mL (IC_50_ − amastigotes) and 4.54 μg/mL (IC_50_ − promastigotes)	Reduction in pro-inflammatory mediators in infected macrophages and NO levels		
		In vitro	Resveratrol analogues	Pure compound	Various (depending on parasite type and stage)	Inhibition of mitochondrial function, membrane permeabilization, cell cycle inhibition, accumulation of autophagic vacuoles	2019	[203]
	*L. braziliensis*	In vitro, in silico	Resveratrol & analogues	Pure compound	28.41 μg/mL (IC_50_ − amastigotes) and 17.15 μg/mL (IC_50_ − promastigotes)	Inhibition of trypanothione reductase	2019	[204]
		In vitro	Resveratrol analogues	Pure compound	Various (depending on parasite type and stage)	Inhibition of mitochondrial function, membrane permeabilization, cell cycle inhibition, accumulation of autophagic vacuoles	2019	[203]
	*L. donovani*	In vitro	Piceatannol	*Euphorbia lagascae* seeds	4.2 and 7.4 μg/mL (LD_50_)	Toxic action—probably protein kinase inhibition	2008	[84]
	*L. infantum*	In vitro	Piceatannol	*Euphorbia lagascae* seeds	3.9 μg/mL (LD_50_)	Toxic action—probably protein kinase inhibition	2008	[84]
		In vitro	Resveratrol	Pure compound	Various (depending on parasite type and stage)	Inhibition of mitochondrial function, membrane permeabilization, cell cycle inhibition, accumulation of autophagic vacuoles	2019	[203]
	*L. major*	In vitro	Resveratrol	Pure compound	45 μg/mL	n/a	2007	[205]
		In vitro	Piceatannol	*Euphorbia lagascae* seeds	5.7 μg/mL (LD_50_)	Toxic action—probably protein kinase inhibition	2008	[84]
		In vitro	Resveratrol analogues	Pure compound	<35 μg/mL (concentration of moderate antipromastigote activity)	Inhibition of NO production and cytotoxicity towards host cells	2013	[206]
		In vitro	Resveratrol	Pure compound	16.23 μg/mL (IC_50_)	n/a	2022	[207]
		In vivo			10 mg/Kg	n/a		
*Plasmodium*	*P. falciparum*	In vitro	Piceid	*Parthenocissus tricuspidate* leaves	5.15 ± 0.47 μg/mL (IC_50_)	n/a	2007	[208]
			Resveratrol		13.24 ± 1.73 μg/mL (IC_50_)			
*Schistosoma*	*S. mansoni*	In vitro	Resveratrol ± praziquantel/artesunate	Pure compound	2.28, 11.41, and 22.82 μg/mL	Synergistic action with antiparasitic drugs	2019	[209]
*Toxoplasma*	*T. gondii*	In vivo	Resveratrol (±sulfamethoxazole-trimethoprim)	Pure compound	Different doses depending on test groups	Reduction in oxidative species and increase in antioxidant species (protective effective for host organism)	2016	[210]
		In vitro	Piceatannol	Pure compound	15 mg/kg	Inhibition of proliferation	2022	[211]
		In vivo			6.86 μg/mL (EC_50_)	Unclear—possibly similar to that of resveratrol		
		In vitro	Piceatannol	Pure compound	24.42 μg/mL (cells treated for 12 h)	Induction of mitochondrial dysfunction and reduction in ATP levels	2025	[212]
		In vivo			50 mg/kg ^2^	n/a		
		In vitro	Resveratrol	Pure compound	12.46 μg/mL (IC_50_)	Reduction in growth, apoptosis; promotion of apoptosis of infected cells	2019	[213]
*Trichinella*	*T. spirallis*	In vitro	Resveratrol	Pure compound	Different concentrations depending on time of exposure and parasite stage	n/a	2009	[214]
*Trypanosoma*		In vivo	Resveratrol	Pure compound	15 mg/kg (starting at 60 days post-infection, for 30 days)	Cardioprotective effect via reduction in parasitaemia, activation of the AMPK pathway, and reduction in oxidative stress	2016	[215]
	*T. cruzi*	In vitro	Resveratrol	Pure compound	17.57 μg/mL (IC_50_ − inhibition of trypomastigote activity); 22.37 μg/mL (IC_50_ − inhibition of epimastigote growth)	Inhibition of trypanosomal activity due to inhibition of arginine kinase	2016	[216]
		In vitro	Resveratrol	Pure compound	57.06 μg/mL (IC_50_ − inhibition of replication); 11.48 μg/mL (IC_50_—trypanosidal activity)	Diminishing epimastigote growth; interfering with life cycle	2017	[217]

^1^ Intracellular and extracellular stage, respectively. ^2^ Only concentration used; mice survived for more days but eventually died.

**Table 5:** Available data on the antiviral potential of the main stilbenoids against DNA viruses, organized alphabetically by family and species, and then chronologically (potency reported as per the original research).

Family	Species	Study Type	Compound	Extract from	Concentration	Mechanism	Year	Reference
Hepadnaviridae	Hepatitis B virus (HBV)	In vitro	Pterostilbene	Pure compound	1.45 μg/mL (EC_50_)	Inhibition of viral replication; inhibition of infected cell proliferation	2021	[270]
		In vitro	Resveratrol	Pure compound	11.41 μg/mL	Inhibition of viral replication; reduction in cytotoxicity in infected cells	2022	[271]
		In vivo			100 mg/kg/day	Decrease in serum DNA in mice		
Orthoherpesviridae	Cytomegalovirus (CMV)	In vitro	Resveratrol	Pure compound	0.23–0.46 μg/mL (IC_50_)	Inhibition of viral replication; inhibition of virus-induced cellular signaling	2004	[272]
		In vitro	Piceatannol	Pure compound	0.24–12.21 μg/mL	Inhibition of viral replication; suppression of mechanisms of cellular senescence	2020	[273]
		In vitro	Pterostilbene	Pure compound	0.34 μg/mL (IC_50_)	Inhibition of cell lysis; inhibition of viral protein expression; suppression of viral replication	2022	[274]
	Duck enteritis virus (DEV)	In vitro	Resveratrol	Pure compound	3.85 μg/mL (IC_50_)	Inhibition of viral replication proteins	2013	[275]
	Epstein–Barr virus (EBV)	In vivo	Resveratrol	Pure compound	16.38 ± 0.45 μg/mL (IC_50_)	Inhibition of early antigen activation; free radical scavenging	2002	[276]
		In vitro	Resveratrol	Pure compound	11.91 μg/mL (EC_50_)	Reduction in virion production	2010	[277]
		In vitro	Resveratrol	Pure compound	Different EC_50_ for different cell lines	Cell cycle arrest and apoptosis in infected cells	2011	[278]
		In vitro	Resveratrol	Pure compound	Different concentrations depending on cell line and protocol	Inhibition of viral protein expression; suppression of redox-sensitive transcription factors	2012	[279]
		In vitro	Resveratrol	Pure compound	5.71 and 11.41 μg/mL	Anti-apoptotic effect of infected cells	2012	[280]
	Herpes simplex virus (HSV)	In vitro	Resveratrol	Pure compound	50 μg/mL	Inhibition of viral replication and reactivation	1999	[281]
		In vivo	Resveratrol	Pure compound	12.5 or 25% resveratrol cream	Suppression of lesion formation	2004	[282]
		In vivo	Resveratrol	Pure compound	19% resveratrol cream	Reduction in viral replication and limitation of disease spread	2005	[283]
		In vitro	Resveratrol	Pure compound	50 μg/mL	Suppression of NF-κB activation; inhibition of viral genome expression and replication	2006	[284]
		In vitro	Resveratrol	Pure compound	6.85 μg/mL	n/a (only resveratrol derivatives were effective)	2012	[285]
	Pseudorabies virus (PRV)	In vitro	Piceatannol	Pure compound	30.7 μg/mL (IC_50_)	Inhibition of viral replication; inhibition of infected cell apoptosis	2023	[286]
		In vivo			50 mg/kg per day	Protective effect (reduction in viral load)		
	Varicella–Zoster virus (VZV)	In vitro	Resveratrol	Pure compound	50 μg/mL	Inhibition of viral replication	2006	[287]
Polyomaviridae	Polyomaviruses	In vitro	Resveratrol	Pure compound	4.56 and 5.71 μg/mL	Inhibition of viral DNA synthesis	2009	[288]

**Table 6:** Available data on the interaction of the stilbenoids discussed in the text with RNA viruses, organized alphabetically by family and species, and then chronologically (potency reported as per the original research).

Family	Species	Study Type	Compound	Extract from	Concentration	Mechanism	Year	Reference
Coronaviridae	SARS-CoV2	In vitro	Pterostilbene	Pure compound	4.87 μg/mL (EC_50_) and 12.05 μg/mL (EC_90_); different concentrations in different experiment types	Inhibition of viral replication inside infected cells	2021	[321]
		In vitro	Resveratrol	Pure compound	15.06 μg/mL (EC_50_) and 27.16 μg/mL (EC_90_); different concentrations in different experiment types	Inhibition of viral replication inside infected cells		
		In vitro	Resveratrol	Pure compound	1.05 μg/mL (EC_50_)	Inhibition of viral replication inside infected cells	2021	[322]
		In vitro	Piceid	*Reynoutria japonica*, *Reynoutria sachalinensis*	n/a (IC_50_ available for all fractions)	Weak Mpro inhibition	2021	[323]
Flaviviridae	Hepatitis C virus (HCV)	In vitro	Resveratrol	Pure compound	n/a (no antiviral effect)	Promotion of viral DNA replication *	2010	[324]
Orthomyxoviridae	Influenza virus	In vitro	Resveratrol	Pure compound	40 μg/mL (for total inhibition of replication)	Inhibition of viral replication inside infected cells	2005	[325]
		In silico	Piceid	Pure compound	n/a (molecular docking study)	Inhibition of viral neuraminidase	2013	[326]
		In vitro	Piceatannol	Pure compound	n/a	Rupture of the viral envelope and inactivation of viral particles; blockade of membrane fusion and of conformational change in hemagglutinin	2023	[327]
		In vivo				Reduction in viral titers and promotion of survival		
		In vitro	Pterostilbene	Pure compound	5.13 μg/mL	Inhibition of viral replication	2023	[328]
		In vivo	Pterostilbene	Pure compound	30 or 60 mg/kg per day	Suppression of inflammation and apoptosis; preservation of epithelial cell integrity; modulation of macrophage polarization	2024	[329]
Picornaviridae	Human enterovirus	In vitro	Resveratrol	Pure compound	30 μg/mL	Inhibition of viral replication and pro-inflammatory cytokine secretion	2015	[330]
		In vitro	Pterostilbene	Pure compound	3.25 μg/mL (EC_50_) (different results for different viral strains)	Inhibition of progeny virus; inhibition of viral replication; inhibition of viral protein production; inhibition of enteroviral attachment	2025	[331]
		In vivo			50 mg/kg	Increase in survival rate; improved disease outcomes (potential stimulation of innate immunity)		
	Human rhinovirus	In vitro	Resveratrol	Pure compound	68.47 μg/mL	Inhibition of viral replication; inhibition of pro-inflammatory factors secretion	2015	[332]
	Theiler’s murine encephalomyelitis virus (TMEV)	In vivo	Resveratrol	Pure compound	20 mg/kg/day	Significant increase in demyelination and inflammation without neuroprotection of the central nervous system *	2013	[333]
Pneumoviridae	Human metapneumovirus	In vitro	Resveratrol	Pure compound	2.28–11.41 μg/mL	Inhibition of viral replication; reduction in cellular oxidative damage; reduction in inflammation	2015	[334]
	Respiratory syncytial virus (RSV)	In vivo	Resveratrol	Pure compound	30 mg/kg per day	Reduction in viral titers, lymphocyte infiltration, inflammation and interferon production	2011	[335]
		In vitro	Resveratrol	Pure compound	Different concentrations depending on experimental protocol	Decrease in IL-6 production and partial inhibition of viral replication	2012	[336]
		In vivo	Resveratrol	Pure compound	30 mg/kg/day	Increase in pro-inflammatory cytokine secretion (TNF-*α*, IFN-*γ*, and IL-2)	2014	[337]
		In vivo	Resveratrol	Pure compound	30 mg/kg/day	Downregulation of MMP-12 and interferon production	2015	[338]
		In vivo	Resveratrol	Pure compound	30 μg/mL	Suppression of persistent airway inflammation	2015	[339]
Retroviridae	Human immunodeficiency virus (HIV)	In vitro	Resveratrol	Pure compound	1.32 μg/mL (EC_50_)	Inhibition of HIV-1 strains replication	2014	[340]

* Not an antiviral mechanism.

**Table 7:** Synopsis of the antifungal mechanisms of the stilbenoids discussed in the text.

Effect (Mechanism)	Fungi	References
Inhibition of aflatoxin production	*A. flavus*	[143]
Inhibition of biofilm formation	*C. albicans*, *T. cutaneum*	[121,148,150,153]
Disruption of mature biofilm	*C. albicans*, *T. cutaneum*	[121,150,153]
Inhibition of fungal growth	*A. fumigatus*, *C. albicans*, *C. herbarum*, *T. rubrum*	[144,145,147]
Inhibition of mycelial growth	*A. flavus*	[143]
Fungicidal effect on cells	*C. albicans*, *E. floccosum*, *M. gypseum*, *Trichophyton* spp., *T. beigelii*	[146,149,152,377]
Promotion of susceptibility to antifungal agents	*C. albicans*	[151]

## Data Availability

Not applicable.

## Abbreviations

The following abbreviations are used in this manuscript:

AIDS	Acquired immune deficiency syndrome
ALI	Air–Liquid Interface
AMPK	5′ adenosine monophosphate-activated protein kinase
AP1	Activator protein 1
ART	Antiretroviral therapy
ATP	Adenosine triphosphate
BKPyV	BK polyomavirus virus
BZLF1	BamHI Z fragment leftward open reading frame 1
CMV	Cytomegalovirus
CNS	Central nervous system
COPD	Chronic obstructive pulmonary disease
DEV	Duck enteritis virus
DNA	Deoxyribonucleic acid
EA-D	Early Antigen D
EBV	Epstein–Barr virus
EC50	Half-maximal effective concentration
EC_90_	Effective concentration 90%
ED50	Half-maximal effective dose
ERK1/2	Extracellular signal-regulated kinase 1/2
EV-71	Enterovirus 71
GIT	Gastrointestinal
HBV	Hepatitis B virus
HCC	Hepatocellular carcinoma cells
HCMV	Human cytomegalovirus
HCV	Hepatitis C virus
HHV-1	Herpes simplex virus 1
HHV-2	Herpes simplex virus 2
HIV-1	Human immunodeficiency virus 1
HSV-1	Herpes simplex virus 1
HSV-2	Herpes simplex virus 2
IC50	Half-maximal inhibitory concentration
IC_75_	Inhibitory concentration 75%
ICAM-1	Intercellular Adhesion Molecule 1
ICP0	Infected cell protein 0
ICP-4	Infected cell protein 4
ICP8	Infected cell protein 8
ICU	Intensive care unit
IE62	Immediate-early protein 62
IFN-γ	Interferon gamma
IKKα	IκB Kinase α
IKKβ	IκB Kinase β
IKKγ	IκB Kinase gamma
IL-2	Interleukin 2
IL-4	Interleukin 4
IL-6	Interleukin 6
IRF-3	Interferon regulatory factor 3
JC	John Cunningham VIRUS
JCPyV	John Cunningham polyomavirus virus
LD50	Lethal dose 50%
LMP1	Latent membrane protein 1
Mcl-1	Myeloid cell leukemia-1
MIC	Minimum inhibitory concentration
MIC50	Minimum inhibitory concentration 50%
MIC_80_	Minimum inhibitory concentration 80%
MiR-155	MicroRNA-155
MiR-34a	MicroRNA-34a
MMP-12	Matrix metalloproteinase-12
Mpro	Main proteases
mRNA	Messenger ribonucleic acid
MS	Multiple sclerosis
NA	Neuraminidase
NF-kappaB	Nuclear factor kappa-light-chain-enhancer of activated B cells
NFκB	Nuclear Factor kappa-light-chain-enhancer of activated B cells
NF-κB p50	Nuclear Factor kappa-light-chain-enhancer of activated B cells p50
NF-κB p65	Nuclear Factor kappa-light-chain-enhancer of activated B cells p65
NGF	Nerve growth factor
NO	Nitric oxide
NS1	Nonstructural protein 1
PPARγ	Peroxisome proliferator-activated receptor gamma
PRV	Pseudorabies virus
RANTES	Regulated upon activation normal T expressed and secreted
RIG-I	Retinoic acid-inducible gene I
ROS	Reactive oxygen species
RRM2	Ribonucleoside-diphosphate reductase subunit M2
RSV	Respiratory syncytial virus
RSV-16	Respiratory syncytial virus-16
Rta	Replication and Transcription Activator
SARM	Selective androgen receptor modulator
SARS-CoV2	Severe acute respiratory syndrome coronavirus 2
SA-β-Gal	Senescence-associated β-galactosidase
S-G2-M	Synthesis-Gap 2-Mitosis
SIgA	Secretory IgA
SLE	Systemic lupus erythematosus
Sp1	Specificity protein 1
STAT-3	Signal transducer and activator of transcription 3
SV40	Simian vacuolating virus 40
TBK1	TANK-binding kinase 1
TMEV	Theiler’s murine encephalomyelitis virus
TNF-α	Tumor necrosis factor alpha
TRIF	TIR-domain-containing adapter-inducing interferon-β
VP1	Viral protein 1
VZV	Varicella–Zoster virus

## References

[1] Kaitin K., DiMasi J. (2011). Pharmaceutical Innovation in the 21st Century: New Drug Approvals in the First Decade, 2000–2009. Clin. Pharmacol. Ther..

[2] de la Torre B.G., Albericio F. (2024). The Pharmaceutical Industry in 2023: An Analysis of FDA Drug Approvals from the Perspective of Molecules. Molecules.

[3] Butt J.H., Bathel J.S., Hosokawa M.C., Moore R.A. (1988). NSAIDs: A clinical approach to the problems of gastrointestinal side-effects. Aliment. Pharmacol. Ther..

[4] Cunha B.A. (2001). Antibiotic Side Effects. Med. Clin. N. Am..

[5] Mackin P. (2008). Cardiac side effects of psychiatric drugs. Hum. Psychopharmacol. Clin. Exp..

[6] Sostres C., Gargallo C.J., Arroyo M.T., Lanas A. (2010). Adverse effects of non-steroidal anti-inflammatory drugs (NSAIDs, aspirin and coxibs) on upper gastrointestinal tract. Best Pract. Res. Clin. Gastroenterol..

[7] Richardson W.L., Hammert W.C. (2014). Adverse Effects of Common Oral Antibiotics. J. Hand Surg..

[8] Rousan T.A., Mathew S.T., Thadani U. (2016). The risk of cardiovascular side effects with anti-anginal drugs. Expert Opin. Drug Saf..

[9] Bețiu A.M., Noveanu L., Hâncu I.M., Lascu A., Petrescu L., Maack C., Elmér E., Muntean D.M. (2022). Mitochondrial Effects of Common Cardiovascular Medications: The Good, the Bad and the Mixed. Int. J. Mol. Sci..

[10] McManus M.C. (1997). Mechanisms of bacterial resistance to antimicrobial agents. Am. J. Health-Syst. Pharm..

[11] Tenover F.C. (2001). Development and Spread of Bacterial Resistance to Antimicrobial Agents: An Overview. Clin. Infect. Dis..

[12] Varela M.F., Stephen J., Lekshmi M., Ojha M., Wenzel N., Sanford L.M., Hernandez A.J., Parvathi A., Kumar S.H. (2021). Bacterial Resistance to Antimicrobial Agents. Antibiotics.

[13] Verma A.R., Vijayakumar M., Mathela C.S., Rao C.V. (2009). In vitro and in vivo antioxidant properties of different fractions of Moringa oleifera leaves. Food Chem. Toxicol..

[14] Tesso H., König W.A., Kubeczka K.-H., Bartnik M., Glowniak K. (2005). Secondary metabolites of *Peucedanum tauricum* fruits. Phytochemistry.

[15] Gilca M., Gaman L., Panait E., Stoian I., Atanasiu V. (2010). Chelidonium majus—An Integrative Review: Traditional Knowledge versus Modern Findings. Forsch. Komplementärmedizin Res. Complement. Med..

[16] Singh N., Bhalla M., de Jager P., Gilca M. (2011). An Overview on Ashwagandha: A Rasayana (Rejuvenator) of Ayurveda. Afr. J. Tradit. Complement. Altern. Med..

[17] Kukula-Koch W., Grabarska A., Łuszczki J., Czernicka L., Nowosadzka E., Gumbarewicz E., Jarząb A., Audo G., Upadhyay S., Głowniak K. (2018). Superior anticancer activity is demonstrated by total extract of *Curcuma longa* L. as opposed to individual curcuminoids separated by centrifugal partition chromatography. Phytother. Res..

[18] Koch W., Kukula-Koch W., Głowniak K. (2019). Catechin Composition and Antioxidant Activity of Black Teas in Relation to Brewing Time. J. AOAC Int..

[19] Petran M., Dragos D., Gilca M. (2020). Historical ethnobotanical review of medicinal plants used to treat children diseases in Romania (1860s–1970s). J. Ethnobiol. Ethnomedicine.

[20] Seidel V. (2020). Plant-Derived Chemicals: A Source of Inspiration for New Drugs. Plants.

[21] Salehi B., Quispe C., Sharifi-Rad J., Cruz-Martins N., Nigam M., Mishra A.P., Konovalov D.A., Orobinskaya V., Abu-Reidah I.M., Zam W. (2021). Phytosterols: From Preclinical Evidence to Potential Clinical Applications. Front. Pharmacol..

[22] Duvoix A., Blasius R., Delhalle S., Schnekenburger M., Morceau F., Henry E., Dicato M., Diederich M. (2005). Chemopreventive and therapeutic effects of curcumin. Cancer Lett..

[23] Boots A.W., Haenen G.R.M.M., Bast A. (2008). Health effects of quercetin: From antioxidant to nutraceutical. Eur. J. Pharmacol..

[24] Hewlings S.J., Kalman D.S. (2017). Curcumin: A Review of Its Effects on Human Health. Foods.

[25] Füchtbauer S., Mousavi S., Bereswill S., Heimesaat M.M. (2021). Antibacterial properties of capsaicin and its derivatives and their potential to fight antibiotic resistance—A literature survey. Eur. J. Microbiol. Immunol..

[26] Periferakis A.-T., Periferakis A., Periferakis K., Caruntu A., Badarau I.A., Savulescu-Fiedler I., Scheau C., Caruntu C. (2023). Antimicrobial Properties of Capsaicin: Available Data and Future Research Perspectives. Nutrients.

[27] Aghababaei F., Hadidi M. (2023). Recent Advances in Potential Health Benefits of Quercetin. Pharmaceuticals.

[28] Periferakis A., Periferakis K., Badarau I.A., Petran E.M., Popa D.C., Caruntu A., Costache R.S., Scheau C., Caruntu C., Costache D.O. (2022). Kaempferol: Antimicrobial Properties, Sources, Clinical, and Traditional Applications. Int. J. Mol. Sci..

[29] Periferakis A., Periferakis A.-T., Troumpata L., Periferakis K., Scheau A.-E., Savulescu-Fiedler I., Caruntu A., Badarau I.A., Caruntu C., Scheau C. (2023). Kaempferol: A Review of Current Evidence of Its Antiviral Potential. Int. J. Mol. Sci..

[30] Petran E.M., Periferakis A., Troumpata L., Periferakis A.-T., Scheau A.-E., Badarau I.A., Periferakis K., Caruntu A., Savulescu-Fiedler I., Sima R.-M. (2024). Capsaicin: Emerging Pharmacological and Therapeutic Insights. Curr. Issues Mol. Biol..

[31] Periferakis A.-T., Adalis G.-M., Periferakis A., Troumpata L., Periferakis K., Dragosloveanu C.D.M., Caruntu A., Savulescu-Fiedler I., Dragosloveanu S., Scheau A.-E. (2025). The Multifaceted Antimicrobial Profile of Piperine in Infectious Disease Management: Current Perspectives and Potential. Pharmaceuticals.

[32] Periferakis A., Troumpata L., Periferakis K., Adalis G., Periferakis A., Georgatos-Garcia S., Maier C., Costache A., Garofil D., Costache D. (2025). Traditional Ethnomedical and Ethnobotanical Applications and Uses of Piper Nigrum. RJ Mil. Med..

[33] Ansari N., Khodagholi F. (2013). Natural products as promising drug candidates for the treatment of Alzheimer’s disease: Molecular mechanism aspect. Curr. Neuropharmacol..

[34] Al-Khayri J.M., Mascarenhas R., Harish H.M., Gowda Y., Lakshmaiah V.V., Nagella P., Al-Mssallem M.Q., Alessa F.M., Almaghasla M.I., Rezk A.A. (2023). Stilbenes, a Versatile Class of Natural Metabolites for Inflammation—An Overview. Molecules.

[35] Guerrero R.F., Biais B., Richard T., Puertas B., Waffo-Teguo P., Merillon J.-M., Cantos-Villar E. (2016). Grapevine cane’s waste is a source of bioactive stilbenes. Ind. Crops Prod..

[36] Chong J., Poutaraud A., Hugueney P. (2009). Metabolism and roles of stilbenes in plants. Plant Sci..

[37] Bábíková P., Vrchotová N., Tříska J., Kyseláková M. (2009). Content of trans-resveratrol in leaves and berries of interspecific grapevine (*Vitis* sp.) varieties. Czech J. Food Sci..

[38] Rusjan D., Halbwirth H., Stich K., Mikulič-Petkovšek M., Veberič R. (2012). Biochemical response of grapevine variety ‘Chardonnay’(*Vitis vinifera* L.) to infection with grapevine yellows (*Bois noir*). Eur. J. Plant Pathol..

[39] Aja-Perez I., Krisa S., Hornedo-Ortega R., Ruiz-Larrea M.B., Ruiz-Sanz J.I., Richard T., Courtois A. (2021). Stilbenes at Low Micromolar Concentrations Mitigate the NO, TNF-α, IL-1β and ROS Production in LPS-Stimulated Murine Macrophages. J. Biol. Act. Prod. Nat..

[40] Lee I.-T., Lin H.-C., Huang T.-H., Tseng C.-N., Cheng H.-T., Huang W.-C., Cheng C.-Y. (2022). Anti-Inflammatory Effect of Resveratrol Derivatives via the Downregulation of Oxidative-Stress-Dependent and c-Src Transactivation EGFR Pathways on Rat Mesangial Cells. Antioxidants.

[41] Çetin E.S., Altinöz D., Tarcan E., Baydar N.G. (2011). Chemical composition of grape canes. Ind. Crops Prod..

[42] Yu O., Jez J.M. (2008). Nature’s assembly line: Biosynthesis of simple phenylpropanoids and polyketides. Plant J..

[43] Jiang S., Wang M., Jiang L., Xie Q., Yuan H., Yang Y., Zafar S., Liu Y., Jian Y., Li B. (2021). The medicinal uses of the genus Bletilla in traditional Chinese medicine: A phytochemical and pharmacological review. J. Ethnopharmacol..

[44] Hillis W., Hasegawa M. (1962). The polyphenols in leaves of Eucalyptus sideroxylon. Biochem. J..

[45] Dou J., Sui M., Malinen K., Pesonen T., Isohanni T., Vuorinen T. (2022). Spruce bark stilbenes as a nature-inspired sun blocker for sunscreens. Green Chem..

[46] Lepak A., Gutmann A., Kulmer S.T., Nidetzky B. (2015). Creating a Water-Soluble Resveratrol-Based Antioxidant by Site-Selective Enzymatic Glucosylation. ChemBioChem.

[47] Du Q.-H., Peng C., Zhang H. (2013). Polydatin: A review of pharmacology and pharmacokinetics. Pharm. Biol..

[48] Schulze K., Schreiber L., Szankowski I. (2005). Inhibiting Effects of Resveratrol and Its Glucoside Piceid against *Venturia inaequalis*, the Causal Agent of Apple Scab. J. Agric. Food Chem..

[49] Piotrowska H., Kucinska M., Murias M. (2012). Biological activity of piceatannol: Leaving the shadow of resveratrol. Mutat. Res./Rev. Mutat. Res..

[50] Cunningham J., Haslam E., Haworth R. (1963). 535. The constitution of piceatannol. J. Chem. Soc..

[51] Periferakis A., Periferakis A.-T., Troumpata L., Periferakis K., Georgatos-Garcia S., Touriki G., Dragosloveanu C.D.M., Caruntu A., Savulescu-Fiedler I., Dragosloveanu S. (2025). Pinosylvin: A Multifunctional Stilbenoid with Antimicrobial, Antioxidant, and Anti-Inflammatory Potential. Curr. Issues Mol. Biol..

[52] Erdtman V. (1939). Tallvedkärnans extraktivämnen och deras inverkan på uppslutningen enligt sulfitmetoden. Sven. Papperstidning.

[53] Silva F., Figueiras A., Gallardo E., Nerín C., Domingues F.C. (2014). Strategies to improve the solubility and stability of stilbene antioxidants: A comparative study between cyclodextrins and bile acids. Food Chem..

[54] Ferrer P., Asensi M., Segarra R., Ortega A., Benlloch M., Obrador E., Varea M.T., Asensio G., Jordá L., Estrela J.M. (2005). Association between pterostilbene and quercetin inhibits metastatic activity of B16 melanoma. Neoplasia.

[55] Waszczuk M., Bianchi S.E., Pittol V., Martiny S., Delagustin M.G., de Carvalho Meirelles G., Benes Raabe V., de Souza Barbosa F., dos Santos Lacerda D., Araújo A.S.R. (2023). The challenge of improving pterostilbene (PTS) solubility for solid and semi-solid dosage forms: The obtention of binary and ternary systems. Int. J. Pharm..

[56] Seshadri T.R. (1972). Polyphenols of Pterocarpus and Dalbergia woods. Phytochemistry.

[57] Tanaka H., Nishimaki-Mogami T., Tamehiro N., Shibata N., Mandai H., Ito S., Wakamatsu K. (2024). Pterostilbene, a Dimethyl Derivative of Resveratrol, Exerts Cytotoxic Effects on Melanin-Producing Cells through Metabolic Activation by Tyrosinase. Int. J. Mol. Sci..

[58] Catalgol B., Batirel S., Taga Y., Ozer N.K. (2012). Resveratrol: French paradox revisited. Front. Pharmacol..

[59] Akinwumi B.C., Bordun K.-A.M., Anderson H.D. (2018). Biological Activities of Stilbenoids. Int. J. Mol. Sci..

[60] Koronowski K.B., Dave K.R., Saul I., Camarena V., Thompson J.W., Neumann J.T., Young J.I., Perez-Pinzon M.A. (2015). Resveratrol Preconditioning Induces a Novel Extended Window of Ischemic Tolerance in the Mouse Brain. Stroke.

[61] Colombo F., Di Lorenzo C., Regazzoni L., Fumagalli M., Sangiovanni E., Peres de Sousa L., Bavaresco L., Tomasi D., Bosso A., Aldini G. (2019). Phenolic profiles and anti-inflammatory activities of sixteen table grape (*Vitis vinifera* L.) varieties. Food Funct..

[62] Ribeiro de Lima M.T., Waffo-Téguo P., Teissedre P.L., Pujolas A., Vercauteren J., Cabanis J.C., Mérillon J.M. (1999). Determination of stilbenes (trans-astringin, cis- and trans-piceid, and cis- and trans-resveratrol) in Portuguese wines. J. Agric. Food Chem..

[63] Tian B., Liu J. (2020). Resveratrol: A review of plant sources, synthesis, stability, modification and food application. J. Sci. Food Agric..

[64] Scheau C., Mihai L., Bădărău I., Caruntu C. (2020). Emerging applications of some important natural compounds in the field of oncology. Farmacia.

[65] Aritomi M., Donnelly D.M.X. (1976). Stilbene glucosides in the bark of Picea sitchensis. Phytochemistry.

[66] Romero-Pérez A.I., Ibern-Gómez M., Lamuela-Raventós R.M., de la Torre-Boronat M.C. (1999). Piceid, the Major Resveratrol Derivative in Grape Juices. J. Agric. Food Chem..

[67] Tolomeo M., Grimaudo S., Di Cristina A., Roberti M., Pizzirani D., Meli M., Dusonchet L., Gebbia N., Abbadessa V., Crosta L. (2005). Pterostilbene and 3′-hydroxypterostilbene are effective apoptosis-inducing agents in MDR and BCR-ABL-expressing leukemia cells. Int. J. Biochem. Cell Biol..

[68] Schmidlin L., Poutaraud A., Claudel P., Mestre P., Prado E., Santos-Rosa M., Wiedemann-Merdinoglu S., Karst F., Merdinoglu D., Hugueney P. (2008). A stress-inducible resveratrol O-methyltransferase involved in the biosynthesis of pterostilbene in grapevine. Plant Physiol..

[69] Paul B., Masih I., Deopujari J., Charpentier C. (1999). Occurrence of resveratrol and pterostilbene in age-old darakchasava, an ayurvedic medicine from India. J. Ethnopharmacol..

[70] Fuendjiep V., Wandji J., Tillequin F., Mulholland D.A., Budzikiewicz H., Fomum Z.T., Nyemba A.M., Koch M. (2002). Chalconoid and stilbenoid glycosides from *Guibourtia tessmanii*. Phytochemistry.

[71] Stivala L.A., Savio M., Carafoli F., Perucca P., Bianchi L., Maga G., Forti L., Pagnoni U.M., Albini A., Prosperi E. (2001). Specific structural determinants are responsible for the antioxidant activity and the cell cycle effects of resveratrol. J. Biol. Chem..

[72] Rimando A.M., Cuendet M., Desmarchelier C., Mehta R.G., Pezzuto J.M., Duke S.O. (2002). Cancer chemopreventive and antioxidant activities of pterostilbene, a naturally occurring analogue of resveratrol. J. Agric. Food Chem..

[73] Manickam M., Ramanathan M., Jahromi M.A., Chansouria J.P., Ray A.B. (1997). Antihyperglycemic activity of phenolics from Pterocarpus marsupium. J. Nat. Prod..

[74] Grover J.K., Vats V., Yadav S.S. (2005). Pterocarpus marsupium extract (Vijayasar) prevented the alteration in metabolic patterns induced in the normal rat by feeding an adequate diet containing fructose as sole carbohydrate. Diabetes Obes. Metab..

[75] Rimando A.M., Nagmani R., Feller D.R., Yokoyama W. (2005). Pterostilbene, a new agonist for the peroxisome proliferator-activated receptor alpha-isoform, lowers plasma lipoproteins and cholesterol in hypercholesterolemic hamsters. J. Agric. Food Chem..

[76] Savulescu-Fiedler I., Dorobantu-Lungu L.R., Dragosloveanu S., Benea S.N., Dragosloveanu C.D.M., Caruntu A., Scheau A.E., Caruntu C., Scheau C. (2025). The Cross-Talk Between the Peripheral and Brain Cholesterol Metabolisms. Curr. Issues Mol. Biol..

[77] Banik K., Ranaware A.M., Harsha C., Nitesh T., Girisa S., Deshpande V., Fan L., Nalawade S.P., Sethi G., Kunnumakkara A.B. (2020). Piceatannol: A natural stilbene for the prevention and treatment of cancer. Pharmacol. Res..

[78] Al-Jaber H.I., Shakya A.K., Al-Qudah M.A., Barhoumi L.M., Abu-Sal H.E., Hasan H.S., Al-Bataineh N., Abu-Orabi S., Mubarak M.S. (2024). Piceatannol, a comprehensive review of health perspectives and pharmacological aspects. Arab. J. Chem..

[79] Kim S., Chen J., Cheng T., Gindulyte A., He J., He S., Li Q., Shoemaker B.A., Thiessen P.A., Yu B. (2024). PubChem 2025 update. Nucleic Acids Res..

[80] Hillis W.E., Carle A. (1962). The origin of the wood and bark polyphenols of *Eucalyptus* species. Biochem. J..

[81] Sut S., Baldan V., Faggian M., Ferrarese I., Maccari E., Teobaldo E., De Zordi N., Bertoni P., Peron G., Dall’Acqua S. (2021). The Bark of *Picea abies* L., a Waste from Sawmill, as a Source of Valuable Compounds: Phytochemical Investigations and Isolation of a Novel Pimarane and a Stilbene Derivative. Plants.

[82] Suprun A.R., Dubrovina A.S., Aleynova O.A., Kiselev K.V. (2021). The Bark of the Spruce *Picea jezoensis* Is a Rich Source of Stilbenes. Metabolites.

[83] Toscano Underwood C.D., Pearce R.B. (1991). Astringin and isorhapontin distribution in Sitka spruce trees. Phytochemistry.

[84] Duarte N., Kayser O., Abreu P., Ferreira M.J. (2008). Antileishmanial activity of piceatannol isolated from *Euphorbia lagascae* seeds. Phytother. Res..

[85] Ku K.L., Chang P.S., Cheng Y.C., Lien C.Y. (2005). Production of stilbenoids from the callus of Arachis hypogaea: A novel source of the anticancer compound piceatannol. J. Agric. Food Chem..

[86] Yang X., He Z., Zheng Y., Wang N., Mulinge M., Schmit J.C., Steinmetz A., Seguin-Devaux C. (2021). Chemical Constituents of Cassia abbreviata and Their Anti-HIV-1 Activity. Molecules.

[87] Tran H.T., Bacher M., Barbini S., Rosenau T., Böhmdorfer S. (2025). Seedy Banana—A Source of Stilbenes and Flavan-3-ols. J. Agric. Food Chem..

[88] Lai T.N., André C., Rogez H., Mignolet E., Nguyen T.B., Larondelle Y. (2015). Nutritional composition and antioxidant properties of the sim fruit (*Rhodomyrtus tomentosa*). Food Chem..

[89] Zomer A.P.L., Rodrigues C.A., Rotta E.M., Vilela Junqueira N.T., Santos O.O., Visentainer J.V., Maldaner L. (2025). Investigation of the Potential of Commercial and Wild *Passiflora* Seed Species as Stilbenes Sources. J. Agric. Food Chem..

[90] Püssa T., Raudsepp P., Kuzina K., Raal A. (2009). Polyphenolic composition of roots and petioles of *Rheum rhaponticum* L.. Phytochem. Anal..

[91] Nopo-Olazabal C., Hubstenberger J., Nopo-Olazabal L., Medina-Bolivar F. (2013). Antioxidant activity of selected stilbenoids and their bioproduction in hairy root cultures of muscadine grape (*Vitis rotundifolia* Michx.). J. Agric. Food Chem..

[92] Gabaston J., Cantos-Villar E., Biais B., Waffo-Teguo P., Renouf E., Corio-Costet M.F., Richard T., Mérillon J.M. (2017). Stilbenes from *Vitis vinifera* L. Waste: A Sustainable Tool for Controlling Plasmopara Viticola. J. Agric. Food Chem..

[93] De Bona G.S., Bertazzon N., Angelini E., Vincenzi S. (2020). Influence of pruning time and viral infection on stilbenoid levels in Pinot noir grape canes. J. Sci. Food Agric..

[94] Kuo C.-H., Chen B.-Y., Liu Y.-C., Chang C.-M.J., Deng T.-S., Chen J.-H., Shieh C.-J. (2014). Optimized Ultrasound-Assisted Extraction of Phenolic Compounds from Polygonum cuspidatum. Molecules.

[95] Sato M., Suzuki Y., Okuda T., Yokotsuka K. (1997). Contents of resveratrol, piceid, and their isomers in commercially available wines made from grapes cultivated in Japan. Biosci. Biotechnol. Biochem..

[96] Lachman J., Kotíková Z., Hejtmánková A., Pivec V., Šulc O., Střalková R., Dědina M. (2016). Resveratrol and piceid isomers concentrations in grapevine shoots, leaves, and tendrils. Hortic. Sci..

[97] Schöppner A., Kindl H. (1984). Purification and properties of a stilbene synthase from induced cell suspension cultures of peanut. J. Biol. Chem..

[98] Schuster R., Holzer W., Doerfler H., Weckwerth W., Viernstein H., Okonogi S., Mueller M. (2016). *Cajanus cajan*—A source of PPARγ activators leading to anti-inflammatory and cytotoxic effects. Food Funct..

[99] Lim S.J., Kim M., Randy A., Nho C.W. (2015). Inhibitory effect of the branches of *Hovenia dulcis* Thunb. and its constituent pinosylvin on the activities of IgE-mediated mast cells and passive cutaneous anaphylaxis in mice. Food Funct..

[100] Fu Y., Sun X., Wang L., Chen S. (2018). Pharmacokinetics and Tissue Distribution Study of Pinosylvin in Rats by Ultra-High-Performance Liquid Chromatography Coupled with Linear Trap Quadrupole Orbitrap Mass Spectrometry. Evid.-Based Complement. Altern. Med..

[101] Labib R.M., Malak L.G., Youssef F.S., Ross S.A. (2020). A new stilbene from *Agonis flexuosa* leaves and verification of its histamine release inhibitory activity using in silico and in vitro studies. S. Afr. J. Bot..

[102] Aalto A.L., Saadabadi A., Lindholm F., Kietz C., Himmelroos E., Marimuthu P., Salo-Ahen O.M., Eklund P., Meinander A. (2023). Stilbenoid compounds inhibit NF-κB-mediated inflammatory responses in the Drosophila intestine. Front. Immunol..

[103] Celimene C.C., Micales J.A., Ferge L., Young R.A. (1999). Efficacy of pinosylvins against white-rot and brown-rot fungi. Holzforschung.

[104] Rowe J., Bower C.L., Wagner E. (1969). Extractives of jack pine bark: Occurrence of cis-and trans-pinosylvin dimethyl ether and ferulic acid esters. Phytochemistry.

[105] Lindberg L., Willför S., Holmbom B. (2004). Antibacterial effects of knotwood extractives on paper mill bacteria. J. Ind. Microbiol. Biotechnol..

[106] Yildirim H., Holmbom B. (1977). Investigations on the wood extractives of pine species from Turkey. Part III. Nonvolatile, nonpolar components in *Pinus brutia* (Henry). Acta Acad. Abo. B.

[107] Sinyeue C., Maerker L., Guentas L., Medevielle V., Bregier F., Chaleix V., Sol V., Lebouvier N. (2023). Polyphenol Content, Antioxidant, and Antibiotic Activities of Pinus Caribaea Morelet Forestry Coproducts. Nat. Prod. Commun..

[108] Willför S.M., Ahotupa M.O., Hemming J.E., Reunanen M.H., Eklund P.C., Sjöholm R.E., Eckerman C.S., Pohjamo S.P., Holmbom B.R. (2003). Antioxidant activity of knotwood extractives and phenolic compounds of selected tree species. J. Agric. Food Chem..

[109] Kodan A., Kuroda H., Sakai F. (2002). A stilbene synthase from Japanese red pine (*Pinus densiflora*): Implications for phytoalexin accumulation and down-regulation of flavonoid biosynthesis. Proc. Natl. Acad. Sci. USA.

[110] Benouadah N., Pranovich A., Aliouche D., Hemming J., Smeds A., Willför S. (2018). Analysis of extractives from *Pinus halepensis* and Eucalyptus camaldulensis as predominant trees in Algeria. Holzforschung.

[111] Wijayanto A., Dumarçay S., Gérardin-Charbonnier C., Sari R.K., Syafii W., Gérardin P. (2015). Phenolic and lipophilic extractives in *Pinus merkusii* Jungh. et de Vries knots and stemwood. Ind. Crops Prod..

[112] Ioannidis K., Melliou E., Alizoti P., Magiatis P. (2017). Identification of black pine (*Pinus nigra* Arn.) heartwood as a rich source of bioactive stilbenes by qNMR. J. Sci. Food Agric..

[113] Suga T., Ohta S., Munesada K., Ide N., Kurokawa M., Shimizu M., Ohta E. (1993). Endogenous pine wood nematicidal substances in pines, *Pinus massoniana*, *P. strobus* and *P. palustris*. Phytochemistry.

[114] Gabaston J., Leborgne C., Waffo-Téguo P., Pedrot E., Richard T., Mérillon J.-M., Valls Fonayet J. (2020). Separation and isolation of major polyphenols from maritime pine (*Pinus pinaster*) knots by two-step centrifugal partition chromatography monitored by LC-MS and NMR spectroscopy. J. Sep. Sci..

[115] Kaushik P., Kaushik D., Khokra S.L. (2013). Ethnobotany and phytopharmacology of *Pinus roxburghii* Sargent: A plant review. J. Integr. Med..

[116] Laavola M., Nieminen R., Leppänen T., Eckerman C., Holmbom B., Moilanen E. (2015). Pinosylvin and monomethylpinosylvin, constituents of an extract from the knot of *Pinus sylvestris*, reduce inflammatory gene expression and inflammatory responses in vivo. J. Agric. Food Chem..

[117] Hemingway R.W., McGraw G.W., Barras S.J. (1977). Polyphenols in Ceratocystis minor infected *Pinus taeda*: Fungal metabolites, phloem and xylem phenols. J. Agric. Food Chem..

[118] Kostecki K., Engelmeier D., Pacher T., Hofer O., Vajrodaya S., Greger H. (2004). Dihydrophenanthrenes and other antifungal stilbenoids from *Stemona* cf. pierrei. Phytochemistry.

[119] Pacher T., Seger C., Engelmeier D., Vajrodaya S., Hofer O., Greger H. (2002). Antifungal stilbenoids from *Stemona collinsae*. J. Nat. Prod..

[120] Adams M., Pacher T., Greger H., Bauer R. (2005). Inhibition of leukotriene biosynthesis by stilbenoids from *Stemona* species. J. Nat. Prod..

[121] Hu D.-D., Zhang R.-L., Zou Y., Zhong H., Zhang E.-S., Luo X., Wang Y., Jiang Y.-Y. (2017). The Structure-Activity Relationship of Pterostilbene Against *Candida albicans* Biofilms. Molecules.

[122] Remsberg C.M., Yáñez J.A., Ohgami Y., Vega-Villa K.R., Rimando A.M., Davies N.M. (2008). Pharmacometrics of pterostilbene: Preclinical pharmacokinetics and metabolism, anticancer, antiinflammatory, antioxidant and analgesic activity. Phytother. Res..

[123] Ren X., An P., Zhai X., Wang S., Kong Q. (2019). The antibacterial mechanism of pterostilbene derived from xinjiang wine grape: A novel apoptosis inducer in Staphyloccocus aureus and Escherichia coli. LWT.

[124] Adrian M., Jeandet P., Douillet-Breuil A.C., Tesson L., Bessis R. (2000). Stilbene content of mature *Vitis vinifera* berries in response to UV-C elicitation. J. Agric. Food Chem..

[125] Zhang J., Zhou L., Zhang P., Liu T., Yang G., Lin R., Zhou J. (2014). Extraction of polydatin and resveratrol from Polygonum cuspidatum root: Kinetics and modeling. Food Bioprod. Process..

[126] Multia E. (2018). Potential and Utilization of Water Extracts from Spruce Bark. Master’s Thesis.

[127] Plumed-Ferrer C., Väkeväinen K., Komulainen H., Rautiainen M., Smeds A., Raitanen J.E., Eklund P., Willför S., Alakomi H.L., Saarela M. (2013). The antimicrobial effects of wood-associated polyphenols on food pathogens and spoilage organisms. Int. J. Food Microbiol..

[128] Duarte A., Alves A.C., Ferreira S., Silva F., Domingues F.C. (2015). Resveratrol inclusion complexes: Antibacterial and anti-biofilm activity against *Campylobacter* spp. and *Arcobacter butzleri*. Food Res. Int..

[129] Liu Y., Zhou J., Qu Y., Yang X., Shi G., Wang X., Hong Y., Drlica K., Zhao X. (2016). Resveratrol Antagonizes Antimicrobial Lethality and Stimulates Recovery of Bacterial Mutants. PLoS ONE.

[130] Angelis A., Hubert J., Aligiannis N., Michalea R., Abedini A., Nuzillard J.-M., Gangloff S.C., Skaltsounis A.-L., Renault J.-H. (2016). Bio-Guided Isolation of Methanol-Soluble Metabolites of Common Spruce (*Picea abies*) Bark by-Products and Investigation of Their Dermo-Cosmetic Properties. Molecules.

[131] Nijampatnam B., Zhang H., Cai X., Michalek S.M., Wu H., Velu S.E. (2018). Inhibition of Streptococcus mutans Biofilms by the Natural Stilbene Piceatannol Through the Inhibition of Glucosyltransferases. ACS Omega.

[132] Nøhr-Meldgaard K., Ovsepian A., Ingmer H., Vestergaard M. (2018). Resveratrol enhances the efficacy of aminoglycosides against Staphylococcus aureus. Int. J. Antimicrob. Agents.

[133] Vaňková E., Paldrychová M., Kašparová P., Lokočová K., Kodeš Z., Maťátková O., Kolouchová I., Masák J. (2020). Natural antioxidant pterostilbene as an effective antibiofilm agent, particularly for gram-positive cocci. World J. Microbiol. Biotechnol..

[134] Vestergaard M., Roshanak S., Ingmer H. (2021). Targeting the ATP Synthase in Staphylococcus aureus Small Colony Variants, Streptococcus pyogenes and Pathogenic Fungi. Antibiotics.

[135] Shih Y.H., Tsai P.J., Chen Y.L., Pranata R., Chen R.J. (2021). Assessment of the Antibacterial Mechanism of Pterostilbene against Bacillus cereus through Apoptosis-like Cell Death and Evaluation of Its Beneficial Effects on the Gut Microbiota. J. Agric. Food Chem..

[136] Brown G.D., Denning D.W., Levitz S.M. (2012). Tackling Human Fungal Infections. Science.

[137] Rodriguez-Tudela J.L., Alastruey-Izquierdo A., Gago S., Cuenca-Estrella M., León C., Miro J.M., Nuñez Boluda A., Ruiz Camps I., Sole A., Denning D.W. (2015). Burden of serious fungal infections in Spain. Clin. Microbiol. Infect..

[138] Alvarez-Moreno C.A., Cortes J.A., Denning D.W. (2018). Burden of Fungal Infections in Colombia. J. Fungi.

[139] Tufa T.B., Denning D.W. (2019). The Burden of Fungal Infections in Ethiopia. J. Fungi.

[140] Fisher M.C., Hawkins N.J., Sanglard D., Gurr S.J. (2018). Worldwide emergence of resistance to antifungal drugs challenges human health and food security. Science.

[141] Revie N.M., Iyer K.R., Robbins N., Cowen L.E. (2018). Antifungal drug resistance: Evolution, mechanisms and impact. Curr. Opin. Microbiol..

[142] Ben-Ami R., Kontoyiannis D.P. (2021). Resistance to Antifungal Drugs. Infect. Dis. Clin. N. Am..

[143] Hu Y.-M., Wang Y.-R., Zhao W.-B., Ding Y.Y., Wu Z.-R., Wang G.-H., Deng P., Zhang S.-Y., An J.-X., Zhang Z.-J. (2023). Efficacy of pterostilbene suppression on Aspergillus flavus growth, aflatoxin B1 biosynthesis and potential mechanisms. Int. J. Food Microbiol..

[144] Välimaa A.L., Honkalampi-Hämäläinen U., Pietarinen S., Willför S., Holmbom B., von Wright A. (2007). Antimicrobial and cytotoxic knotwood extracts and related pure compounds and their effects on food-associated microorganisms. Int. J. Food Microbiol..

[145] Lee S.K., Lee H.J., Min H.Y., Park E.J., Lee K.M., Ahn Y.H., Cho Y.J., Pyee J.H. (2005). Antibacterial and antifungal activity of pinosylvin, a constituent of pine. Fitoterapia.

[146] Jung H.J., Hwang I.A., Sung W.S., Kang H., Kang B.S., Seu Y.B., Lee D.G. (2005). Fungicidal Effect of Resveratrol on Human Infectious Fungi. Arch. Pharmacal Res..

[147] Kingsbury J.M., Heitman J., Pinnell S.R. (2012). Calcofluor White Combination Antifungal Treatments for Trichophyton rubrum and Candida albicans. PLoS ONE.

[148] Li D.D., Zhao L.X., Mylonakis E., Hu G.H., Zou Y., Huang T.K., Yan L., Wang Y., Jiang Y.Y. (2014). In vitro and in vivo activities of pterostilbene against Candida albicans biofilms. Antimicrob. Agents Chemother..

[149] Houillé B., Papon N., Boudesocque L., Bourdeaud E., Besseau S., Courdavault V., Enguehard-Gueiffier C., Delanoue G., Guérin L., Bouchara J.-P. (2014). Antifungal activity of resveratrol derivatives against *Candida* species. J. Nat. Prod..

[150] Kolouchová I., Maťátková O., Paldrychová M., Kodeš Z., Kvasničková E., Sigler K., Čejková A., Šmidrkal J., Demnerová K., Masák J. (2018). Resveratrol, pterostilbene, and baicalein: Plant-derived anti-biofilm agents. Folia Microbiol..

[151] Wang J., Zhang X., Gao L., Wang L., Song F., Zhang L., Wan Y. (2021). The synergistic antifungal activity of resveratrol with azoles against Candida albicans. Lett. Appl. Microbiol..

[152] Chan M.M.-Y. (2002). Antimicrobial effect of resveratrol on dermatophytes and bacterial pathogens of the skin. Biochem. Pharmacol..

[153] Paldrychová M., Kolouchová I., Vaňková E., Maťátková O., Šmidrkal J., Krmela A., Schulzová V., Hajšlová J., Masák J. (2019). Effect of resveratrol and Regrapex-R-forte on Trichosporon cutaneum biofilm. Folia Microbiol..

[154] Mellikeche W., Ricelli A., Abukhmaish M., Caracciolo R., Gallo M., Casini C., Colelli G., D’Onghia A.M., Valentini F. (2025). Aspergillus in Italian Pistachios: Characterization and Detection of Major Aflatoxigenic Species With a Loop-Mediated Isothermal Amplification Assay. Food Sci. Nutr..

[155] Melguizo C., Tarazona A., Gil-Serna J., Mateo F., Patiño B., Mateo E.M. (2025). Toxigenic Aspergillus Diversity and Mycotoxins in Organic Spanish Grape Berries. Toxins.

[156] Awafong P.M., Okechukwu V.O., Fagbohun T.R., Adelusi O.A., Adebo O.A., Njobeh P.B., Mthombeni J.Q. (2025). Greener Solutions in Aflatoxin Management: Transitioning from Conventional Binders to Green Nanotechnology. Nanomaterials.

[157] Shallcross L.J., Davies D.S. (2014). Antibiotic overuse: A key driver of antimicrobial resistance. Br. J. Gen. Pr..

[158] Kwon-Chung K.J., Sugui J.A. (2013). Aspergillus fumigatus--what makes the species a ubiquitous human fungal pathogen?. PLoS Pathog..

[159] Periferakis A., Troumpata L., Periferakis A.-T., Periferakis K. Coal and Asbestos Mining-Induced Pulmonary Pathologies: Radiological Diagnosis and Associated Challenges. Proceedings of the 17th International Congress of the Geological Society of Greece.

[160] Hohl T.M., Feldmesser M. (2007). Aspergillus fumigatus: Principles of pathogenesis and host defense. Eukaryot. Cell.

[161] Bosetti D., Neofytos D. (2023). Invasive Aspergillosis and the Impact of Azole-resistance. Curr. Fungal Infect. Rep..

[162] Mayer F.L., Wilson D., Hube B. (2013). Candida albicans pathogenicity mechanisms. Virulence.

[163] Netea M.G., Gow N.A., Munro C.A., Bates S., Collins C., Ferwerda G., Hobson R.P., Bertram G., Hughes H.B., Jansen T. (2006). Immune sensing of Candida albicans requires cooperative recognition of mannans and glucans by lectin and Toll-like receptors. J. Clin. Investig..

[164] Pappas P.G., Kauffman C.A., Andes D.R., Clancy C.J., Marr K.A., Ostrosky-Zeichner L., Reboli A.C., Schuster M.G., Vazquez J.A., Walsh T.J. (2015). Clinical Practice Guideline for the Management of Candidiasis: 2016 Update by the Infectious Diseases Society of America. Clin. Infect. Dis..

[165] Bakrim S., Machate H., Benali T., Sahib N., Jaouadi I., Omari N.E., Aboulaghras S., Bangar S.P., Lorenzo J.M., Zengin G. (2022). Natural Sources and Pharmacological Properties of Pinosylvin. Plants.

[166] Weber K., Schulz B., Ruhnke M. (2011). Resveratrol and its antifungal activity against *Candida* species. Mycoses.

[167] Fung F., Hughson W.G. (2003). Health effects of indoor fungal bioaerosol exposure. Appl. Occup. Environ. Hyg..

[168] Pedro Abreu J., Esteves J., Boncoraglio M.T., Pereira F.M., Costa C., Oliveira C. (2020). *Cladosporium herbarum* Hot-Tub Lung Hypersensitivity Pneumonitis in a Greenhouse Worker. Eur. J. Case Rep. Intern. Med..

[169] Rid R., Onder K., Hawranek T., Laimer M., Bauer J.W., Holler C., Simon-Nobbe B., Breitenbach M. (2010). Isolation and immunological characterization of a novel *Cladosporium herbarum* allergen structurally homologous to the alpha/beta hydrolase fold superfamily. Mol. Immunol..

[170] Liu J., Ge L., Mei H., Zheng H., Peng J., Liang G., Liu W. (2021). Comparative Genomics and Molecular Analysis of Epidermophyton floccosum. Mycopathologia.

[171] Nokdhes Y.N., Leeyaphan C., Jirawattanadon P., Pongkittilar B., Sereeaphinan C., Bunyaratavej S. (2024). Prevalence and characteristics of Epidermophyton floccosum skin infections: A 12-year retrospective study. Mycoses.

[172] Besrour R., Mtibaa L., Rabhi F., Baccouchi N., Dhaoui A., Jemli B. (2025). Epidermophyton floccosum, an etiological agent of tinea pedis and tinea unguium: About two cases. Pan Afr. Med. J..

[173] Cofone L., Paglione L., Grassi F., Patania F., Pindinello I., Quarantiello A., Sabato M. (2025). Trichophyton mentagrophytes genotype VII, an emerging infection: A Systematic Review. Ann. Di Ig. Med. Prev. E Di Comunita.

[174] Xiao W., Wu Z., Zhang J., Wan J., Zhang R., Xiang X., Yu Y., Fu L., Yang K., Chen Y. (2025). Stage-specific RNA regulomes of Trichophyton mentagrophytes: mRNA-lncRNA-miRNA interplay in spore-hypha transition. IMA Fungus.

[175] Javed M.W., Raikar D., Nagure A.B., Guruprasad K., Siddiqui M.I., Takalkar A.A. (2025). Trichophyton Mentagrophytes: An Emerging Cause of Tinea Capitis in Rural Part of North Karnataka. Indian Dermatol. Online J..

[176] Lohariwala A., Gupta S., Kaur N., Mahendra A. (2025). Emerging Antifungal Resistance in Dermatophytosis: A Clinicomycological Study From North India. Cureus.

[177] Gupta A.K., Susmita Nguyen H.C., Liddy A., Economopoulos V., Wang T. (2025). Terbinafine Resistance in Trichophyton rubrum and Trichophyton indotineae: A Literature Review. Antibiotics.

[178] Ha N.G., Bang Y.J., Lee W.J. (2025). Epidemiological Trends and Clinical Features of Trichophyton rubrum Infections: A 10-Year Retrospective Review of 38,391 Cases (2014–2023). J. Korean Med. Sci..

[179] Song G., Xie W., Kong X., Zheng H., Tsui C.K.M., Xiaodong S., Liu W., Li X., Liang G. (2025). The First Isolation of Multiple Antifungal-Drug-Resistant Trichophyton Rubrum in China and the Novel Resistance Mechanism. Mycoses.

[180] Eshkaleti M.N., Hashemi S.J., Khodavaisy S., Daie Ghazvini R., Ahmadikia K., Foroshani A.R., Ahmadi A., Ghasemi N. (2025). Shifting etiological agents of dermatophytosis: A molecular epidemiological study from Iran. Iran. J. Microbiol..

[181] Futatsuya T., Anzawa K., Mochizuki T., Shimizu A. (2023). Trichophyton tonsurans Infection. Med. Mycol. J..

[182] Firooz A., Lotfali E., Fattahi M., Fattahi M., Miramin Mohammadi A., Shahrzad Kavkani M. (2021). A Case of Terbinafine-Resistant Tinea Cruris Caused by Trichophyton tonsurans. Case Rep. Dermatol. Med..

[183] Samaddar S., Redhwan M.A.M., Eraiah M.M., Koneri R. (2025). Clinical isolates of the anthropophilic dermatophyte Trichophyton tonsurans exhibit transcriptional regulation of multidrug efflux transporters that induce antifungal resistance. Mol. Biol. Rep..

[184] Walsh T.J., Melcher G.P., Rinaldi M.G., Lecciones J., McGough D.A., Kelly P., Lee J., Callender D., Rubin M., Pizzo P.A. (1990). Trichosporon beigelii, an emerging pathogen resistant to amphotericin B. J. Clin. Microbiol..

[185] Couto R., Couto G., Abrahão I., Compagnoni I., Carnio T., Tolentino J. (2021). Endocarditis due to Trichosporon beigelii 11 years after mitral valve replacement. Rev. Port. De Cardiol..

[186] Nakagawa T., Nakashima K., Takaiwa T., Negayama K. (2000). *Trichosporon cutaneum* (*Trichosporon asahii*) infection mimicking hand eczema in a patient with leukemia. J. Am. Acad. Dermatol..

[187] Piecuch A., Cal M., Ogórek R. (2024). Adhesion and biofilm formation by two clinical isolates of *Trichosporon cutaneum* in various environmental conditions. Braz. J. Microbiol..

[188] Milder J.E., Walzer P.D., Kilgore G., Rutherford I., Klein M. (1981). Clinical features of *Strongyloides stercoralis* infection in an endemic area of the United States. Gastroenterology.

[189] Marsh K., Snow R.W. (1997). Host—Parasite interaction and morbidity in malaria endemic areas. Philos. Trans. R. Soc. B Biol. Sci..

[190] Barry M.A., Weatherhead J.E., Hotez P.J., Woc-Colburn L. (2013). Childhood parasitic infections endemic to the United States. Pediatr. Clin. N. Am..

[191] Mata L. (1982). Sociocultural factors in the control and prevention of parasitic diseases. Rev. Infect. Dis..

[192] Fèvre E.M., Bronsvoort B.M.d.C., Hamilton K.A., Cleaveland S. (2006). Animal movements and the spread of infectious diseases. Trends Microbiol..

[193] Torgerson P.R. (2013). One world health: Socioeconomic burden and parasitic disease control priorities. Vet. Parasitol..

[194] Torgerson P.R., de Silva N.R., Fèvre E.M., Kasuga F., Rokni M.B., Zhou X.N., Sripa B., Gargouri N., Willingham A.L., Stein C. (2014). The global burden of foodborne parasitic diseases: An update. Trends Parasitol..

[195] Pisarski K. (2019). The Global Burden of Disease of Zoonotic Parasitic Diseases: Top 5 Contenders for Priority Consideration. Trop. Med. Infect. Dis..

[196] Sangster N., Batterham P., Chapman H.D., Duraisingh M., Le Jambre L., Shirley M., Upcroft J., Upcroft P. (2002). Resistance to antiparasitic drugs: The role of molecular diagnosis. Int. J. Parasitol..

[197] Geary T.G., Thompson D.P. (2003). Development of antiparasitic drugs in the 21st century. Vet. Parasitol..

[198] Loos J.A., Franco M., Chop M., Rodriguez Rodrigues C., Cumino A.C. (2023). Resveratrol against *Echinococcus* sp.: Discrepancies between In Vitro and In Vivo Responses. Trop. Med. Infect. Dis..

[199] Giri B.R., Bharti R.R., Roy B. (2015). In vivo anthelmintic activity of Carex baccans and its active principle resveratrol against *Hymenolepis diminuta*. Parasitol. Res..

[200] Zaman A., Noor S., Ahmad I., Shehroz M., Alhajri N., Ahmed S., Nishan U., Sheheryar S., Ullah R., Shahat A.A. (2025). Exploring cotton plant compounds for novel treatments against brain-eating *Naegleria fowleri*: An In-silico approach. PLoS ONE.

[201] Ferreira C., Soares D.C., Nascimento M.T., Pinto-da-Silva L.H., Sarzedas C.G., Tinoco L.W., Saraiva E.M. (2014). Resveratrol is active against *Leishmania amazonensis*: In vitro effect of its association with Amphotericin B. Antimicrob. Agents Chemother..

[202] Passos C.L., Ferreira C., Soares D.C., Saraiva E.M. (2015). Leishmanicidal Effect of Synthetic trans-Resveratrol Analogs. PLoS ONE.

[203] Antinarelli L.M.R., Meinel R.S., Coelho E.A.F., da Silva A.D., Coimbra E.S. (2019). Resveratrol analogues present effective antileishmanial activity against promastigotes and amastigotes from distinct *Leishmania* species by multitarget action in the parasites. J. Pharm. Pharmacol..

[204] da Silva A.D., Dos Santos J.A., Machado P.A., Alves L.A., Laque L.C., de Souza V.C., Coimbra E.S., Capriles P. (2019). Insights about resveratrol analogs against trypanothione reductase of Leishmania braziliensis: Molecular modeling, computational docking and in vitro antileishmanial studies. J. Biomol. Struct. Dyn..

[205] Kedzierski L., Curtis J.M., Kaminska M., Jodynis-Liebert J., Murias M. (2007). In vitro antileishmanial activity of resveratrol and its hydroxylated analogues against Leishmania major promastigotes and amastigotes. Parasitol. Res..

[206] Lucas I.K., Kolodziej H. (2013). In vitro antileishmanial activity of resveratrol originates from its cytotoxic potential against host cells. Planta Med..

[207] Mousavi P., Rahimi Esboei B., Pourhajibagher M., Fakhar M., Shahmoradi Z., Hejazi S.H., Hassannia H., Nasrollahi Omran A., Hasanpour H. (2022). Anti-leishmanial effects of resveratrol and resveratrol nanoemulsion on Leishmania major. BMC Microbiol..

[208] Son I.H., Chung I.M., Lee S.J., Moon H.I. (2007). Antiplasmodial activity of novel stilbene derivatives isolated from *Parthenocissus tricuspidata* from South Korea. Parasitol. Res..

[209] Gouveia M.J., Brindley P.J., Azevedo C., Gärtner F., da Costa J.M.C., Vale N. (2019). The antioxidants resveratrol and N-acetylcysteine enhance anthelmintic activity of praziquantel and artesunate against *Schistosoma mansoni*. Parasit. Vectors.

[210] Bottari N.B., Baldissera M.D., Tonin A.A., Rech V.C., Alves C.B., D’Avila F., Thomé G.R., Guarda N.S., Moresco R.N., Camillo G. (2016). Synergistic effects of resveratrol (free and inclusion complex) and sulfamethoxazole-trimetropim treatment on pathology, oxidant/antioxidant status and behavior of mice infected with Toxoplasma gondii. Microb. Pathog..

[211] Jiang Y., Shi Y., Hu D., Song X. (2022). The anti-Toxoplasma activity of the plant natural phenolic compound piceatannol. Front. Vet. Sci..

[212] Liu Z., Qiu H., Jiang Y., Mo Y., Lu L., Wang Y., Hu D., Song X. (2025). Piceatannol Induces Mitochondrial Dysfunction in Toxoplasma gondii. Microorganisms.

[213] Chen Q.W., Dong K., Qin H.X., Yang Y.K., He J.L., Li J., Zheng Z.W., Chen D.L., Chen J.P. (2019). Direct and Indirect Inhibition Effects of Resveratrol against Toxoplasma gondii Tachyzoites In Vitro. Antimicrob. Agents Chemother..

[214] Ozkoc S., Tuncay S., Delibas S.B., Akisu C. (2009). In vitro effects of resveratrol on Trichinella spiralis. Parasitol. Res..

[215] Vilar-Pereira G., Carneiro V.C., Mata-Santos H., Vicentino A.R., Ramos I.P., Giarola N.L., Feijó D.F., Meyer-Fernandes J.R., Paula-Neto H.A., Medei E. (2016). Resveratrol Reverses Functional Chagas Heart Disease in Mice. PLoS Pathog..

[216] Valera Vera E.A., Sayé M., Reigada C., Damasceno F.S., Silber A.M., Miranda M.R., Pereira C.A. (2016). Resveratrol inhibits Trypanosoma cruzi arginine kinase and exerts a trypanocidal activity. Int. J. Biol. Macromol..

[217] Campo V.A. (2017). Comparative effects of histone deacetylases inhibitors and resveratrol on Trypanosoma cruzi replication, differentiation, infectivity and gene expression. Int. J. Parasitol. Drugs Drug Resist..

[218] McManus D.P., Zhang W., Li J., Bartley P.B. (2003). Echinococcosis. Lancet.

[219] Zhang W., Wen H., Li J., Lin R., McManus D.P. (2012). Immunology and immunodiagnosis of cystic echinococcosis: An update. Clin. Dev. Immunol..

[220] Dhar P., Chaudhary A., Desai R., Agarwal A., Sachdev A. (1996). Current trends in the diagnosis and management of cystic hydatid disease of the liver. J. Commun. Dis..

[221] Sayek I., Tirnaksiz M.B., Dogan R. (2004). Cystic hydatid disease: Current trends in diagnosis and management. Surg. Today.

[222] Mansfield B.S., Pieton K., Pather S. (2019). Spinal Cystic Echinococcosis. Am. J. Trop. Med. Hyg..

[223] Kern P., Menezes da Silva A., Akhan O., Müllhaupt B., Vizcaychipi K.A., Budke C., Vuitton D.A. (2017). The Echinococcoses: Diagnosis, Clinical Management and Burden of Disease. Adv. Parasitol..

[224] McMillan B., Kelly A., Walker J. (1971). Prevalence of *Hymenolepis diminuta* infection in man in the New Guinea highlands. Trop. Geogr. Med..

[225] Stafford E.E., Sudomo M., Masri S., Brown R.J. (1980). Human parasitoses in Bali, Indonesia. Southeast Asian J. Trop. Med. Public Health.

[226] Tena D., Pérez Simón M., Gimeno C., Pérez Pomata M.T., Illescas S., Amondarain I., González A., Domínguez J., Bisquert J. (1998). Human infection with *Hymenolepis diminuta*: Case report from Spain. J. Clin. Microbiol..

[227] Tiwari S., Karuna T., Rautaraya B. (2014). *Hymenolepis diminuta* Infection in a Child from a Rural Area: A Rare Case Report. J. Lab. Physicians.

[228] Grace E., Asbill S., Virga K. (2015). *Naegleria fowleri*: Pathogenesis, diagnosis, and treatment options. Antimicrob. Agents Chemother..

[229] Zhang H., Cheng X. (2021). Various brain-eating amoebae: The protozoa, the pathogenesis, and the disease. Front. Med..

[230] Kofman A., Guarner J. (2022). Infections Caused by Free-Living Amoebae. J. Clin. Microbiol..

[231] Haston J.C., Cope J.R. (2023). Amebic encephalitis and meningoencephalitis: An update on epidemiology, diagnostic methods, and treatment. Curr. Opin. Infect. Dis..

[232] Cárdenas-Zúñiga R., Silva-Olivares A., Villalba-Magdaleno J.A., Sánchez-Monroy V., Serrano-Luna J., Shibayama M. (2017). Amphotericin B induces apoptosis-like programmed cell death in *Naegleria fowleri* and *Naegleria gruberi*. Microbiology.

[233] Güémez A., García E. (2021). Primary Amoebic Meningoencephalitis by *Naegleria fowleri*: Pathogenesis and Treatments. Biomolecules.

[234] Andrade-Narváez F.J., Vargas-González A., Canto-Lara S.B., Damián-Centeno A.G. (2001). Clinical picture of cutaneous leishmaniases due to *Leishmania* (*Leishmania*) mexicana in the Yucatan peninsula, Mexico. Mem. Inst. Oswaldo Cruz.

[235] Mann S., Frasca K., Scherrer S., Henao-Martínez A.F., Newman S., Ramanan P., Suarez J.A. (2021). A Review of Leishmaniasis: Current Knowledge and Future Directions. Curr. Trop. Med. Rep..

[236] Garlapati R., Iniguez E., Serafim T.D., Mishra P.K., Rooj B., Sinha B., Valenzuela J.G., Srikantiah S., Bern C., Kamhawi S. (2021). Towards a sustainable vector-control strategy in the post kala-azar elimination era. Front. Cell. Infect. Microbiol..

[237] Frézard F., Demicheli C., Ribeiro R.R. (2009). Pentavalent antimonials: New perspectives for old drugs. Molecules.

[238] Marques S.A., Merlotto M.R., Ramos P.M., Marques M.E.A. (2019). American tegumentary leishmaniasis: Severe side effects of pentavalent antimonial in a patient with chronic renal failure. An. Bras. Dermatol..

[239] Periferakis A., Caruntu A., Periferakis A.T., Scheau A.E., Badarau I.A., Caruntu C., Scheau C. (2022). Availability, Toxicology and Medical Significance of Antimony. Int. J. Env. Res. Public Health.

[240] Hefnawy A., Berg M., Dujardin J.C., De Muylder G. (2017). Exploiting Knowledge on Leishmania Drug Resistance to Support the Quest for New Drugs. Trends Parasitol..

[241] Ashley E.A., Pyae Phyo A., Woodrow C.J. (2018). Malaria. Lancet.

[242] Maier A.G., Matuschewski K., Zhang M., Rug M. (2019). Plasmodium falciparum. Trends Parasitol..

[243] Menard D., Dondorp A. (2017). Antimalarial Drug Resistance: A Threat to Malaria Elimination. Cold Spring Harb. Perspect. Med..

[244] Steinmann P., Keiser J., Bos R., Tanner M., Utzinger J. (2006). Schistosomiasis and water resources development: Systematic review, meta-analysis, and estimates of people at risk. Lancet Infect. Dis..

[245] King C.H., Dangerfield-Cha M. (2008). The unacknowledged impact of chronic schistosomiasis. Chronic Illn..

[246] Hotez P.J., Alvarado M., Basáñez M.G., Bolliger I., Bourne R., Boussinesq M., Brooker S.J., Brown A.S., Buckle G., Budke C.M. (2014). The global burden of disease study 2010: Interpretation and implications for the neglected tropical diseases. PLOS Neglected Trop. Dis..

[247] Andrade G., Bertsch D.J., Gazzinelli A., King C.H. (2017). Decline in infection-related morbidities following drug-mediated reductions in the intensity of Schistosoma infection: A systematic review and meta-analysis. PLoS Neglected Trop. Dis..

[248] Evan Secor W. (2014). Water-based interventions for schistosomiasis control. Pathog. Glob. Health.

[249] Lo N.C., Addiss D.G., Hotez P.J., King C.H., Stothard J.R., Evans D.S., Colley D.G., Lin W., Coulibaly J.T., Bustinduy A.L. (2017). A call to strengthen the global strategy against schistosomiasis and soil-transmitted helminthiasis: The time is now. Lancet Infect. Dis..

[250] Colley D.G., Andros T.S., Campbell C.H. (2017). Schistosomiasis is more prevalent than previously thought: What does it mean for public health goals, policies, strategies, guidelines and intervention programs?. Infect. Dis. Poverty.

[251] Boissier J., Grech-Angelini S., Webster B.L., Allienne J.F., Huyse T., Mas-Coma S., Toulza E., Barré-Cardi H., Rollinson D., Kincaid-Smith J. (2016). Outbreak of urogenital schistosomiasis in Corsica (France): An epidemiological case study. Lancet Infect. Dis..

[252] Alonso D., Muñoz J., Gascón J., Valls M.E., Corachan M. (2006). Failure of standard treatment with praziquantel in two returned travelers with Schistosoma haematobium infection. Am. J. Trop. Med. Hyg..

[253] Vale N., Gouveia M.J., Rinaldi G., Brindley P.J., Gärtner F., Correia da Costa J.M. (2017). Praziquantel for Schistosomiasis: Single-Drug Metabolism Revisited, Mode of Action, and Resistance. Antimicrob. Agents Chemother..

[254] Khairullah A.R., Kurniawan S.C., Widodo A., Effendi M.H., Hasib A., Silaen O.S.M., Ramandinianto S.C., Moses I.B., Riwu K.H.P., Yanestria S.M. (2024). A comprehensive review of toxoplasmosis: Serious threat to human health. Open Public Health J..

[255] Li J., Zhao J., Yang X., Wen Y., Huang L., Ma D., Shi J. (2021). One severe case of congenital toxoplasmosis in China with good response to azithromycin. BMC Infect. Dis..

[256] Soleymani E., Babamahmoodi F., Davoodi L., Marofi A., Nooshirvanpour P. (2018). Toxoplasmic Encephalitis in an AIDS Patient with Normal CD4 Count: A Case Report. Iran. J. Parasitol..

[257] Hopper A.T., Brockman A., Wise A., Gould J., Barks J., Radke J.B., Sibley L.D., Zou Y., Thomas S. (2019). Discovery of Selective Toxoplasma gondii Dihydrofolate Reductase Inhibitors for the Treatment of Toxoplasmosis. J. Med. Chem..

[258] Bilska-Zajac E., Tonanzi D., Pozio E., Rozycki M., Cencek T., Thompson P.C., Rosenthal B.M., La Rosa G. (2021). Genetic evidence substantiates transmission of Trichinella spiralis from one swine farm to another. Parasites Vectors.

[259] Pavel R., Ursoniu S., Lupu M.A., Olariu T.R. (2023). Trichinellosis in Hospitalized Children and Adults from Western Romania: A 11-Year Retrospective Study. Life.

[260] Gottstein B., Pozio E., Nöckler K. (2009). Epidemiology, diagnosis, treatment, and control of trichinellosis. Clin. Microbiol. Rev..

[261] Rossi I.V., de Souza D.A.S., Ramirez M.I. (2024). The End Justifies the Means: Chagas Disease from a Perspective of the Host–Trypanosoma cruzi Interaction. Life.

[262] Rassi A., Rassi A., Marin-Neto J.A. (2010). Chagas disease. Lancet.

[263] García-Huertas P., Cardona-Castro N. (2021). Advances in the treatment of Chagas disease: Promising new drugs, plants and targets. Biomed. Pharmacother..

[264] Keay S.K. (1991). Morbidity and mortality associated with herpes virus infections. Trans. Assoc. Life Insur. Med. Dir. Am..

[265] Trigg B.J., Ferguson B.J. (2015). Functions of DNA damage machinery in the innate immune response to DNA virus infection. Curr. Opin. Virol..

[266] Periferakis A., Bolocan A., Ion D. (2022). A review of innovation in medicine. Technol. Innov. Life Sci..

[267] Waheed Y., Sah R., Muhammad K. (2023). Recent developments in vaccines for viral diseases. Vaccines.

[268] Morfin F., Thouvenot D. (2003). Herpes simplex virus resistance to antiviral drugs. J. Clin. Virol..

[269] Zoulim F. (2011). Hepatitis B virus resistance to antiviral drugs: Where are we going?. Liver Int..

[270] Wang R., Xu Z., Tian J., Liu Q., Dong J., Guo L., Hai B., Liu X., Yao H., Chen Z. (2021). Pterostilbene inhibits hepatocellular carcinoma proliferation and HBV replication by targeting ribonucleotide reductase M2 protein. Am. J. Cancer Res..

[271] Pan P., Li J., Lin W., Long G. (2022). Effects of Resveratrol on Hepatitis B Virus Replication: In vitro and in vivo Experiments. Intervirology.

[272] Evers D.L., Wang X., Huong S.M., Huang D.Y., Huang E.S. (2004). 3,4′,5-Trihydroxy-trans-stilbene (resveratrol) inhibits human cytomegalovirus replication and virus-induced cellular signaling. Antivir. Res..

[273] Wang S.Y., Zhang J., Xu X.G., Su H.L., Xing W.M., Zhang Z.S., Jin W.H., Dai J.H., Wang Y.Z., He X.Y. (2020). Inhibitory effects of piceatannol on human cytomegalovirus (hCMV) in vitro. J. Microbiol..

[274] Wang S., Zhou X., He X., Ma S., Sun C., Zhang J., Xu X., Jin W., Yan J., Lin P. (2022). Suppressive effects of pterostilbene on human cytomegalovirus (HCMV) infection and HCMV-induced cellular senescence. Virol. J..

[275] Xu J., Yin Z., Li L., Cheng A., Jia R., Song X., Lu H., Dai S., Lv C., Liang X. (2013). Inhibitory effect of resveratrol against duck enteritis virus in vitro. PLoS ONE.

[276] Kapadia G.J., Azuine M.A., Tokuda H., Takasaki M., Mukainaka T., Konoshima T., Nishino H. (2002). Chemopreventive effect of resveratrol, sesamol, sesame oil and sunflower oil in the Epstein-Barr virus early antigen activation assay and the mouse skin two-stage carcinogenesis. Pharmacol. Res..

[277] Yiu C.Y., Chen S.Y., Chang L.K., Chiu Y.F., Lin T.P. (2010). Inhibitory effects of resveratrol on the Epstein-Barr virus lytic cycle. Molecules.

[278] De Leo A., Arena G., Stecca C., Raciti M., Mattia E. (2011). Resveratrol inhibits proliferation and survival of Epstein Barr virus-infected Burkitt’s lymphoma cells depending on viral latency program. Mol. Cancer Res..

[279] De Leo A., Arena G., Lacanna E., Oliviero G., Colavita F., Mattia E. (2012). Resveratrol inhibits Epstein Barr Virus lytic cycle in Burkitt’s lymphoma cells by affecting multiple molecular targets. Antivir. Res..

[280] Espinoza J.L., Takami A., Trung L.Q., Kato S., Nakao S. (2012). Resveratrol prevents EBV transformation and inhibits the outgrowth of EBV-immortalized human B cells. PLoS ONE.

[281] Docherty J.J., Fu M.M., Stiffler B.S., Limperos R.J., Pokabla C.M., DeLucia A.L. (1999). Resveratrol inhibition of herpes simplex virus replication. Antivir. Res..

[282] Docherty J.J., Smith J.S., Fu M.M., Stoner T., Booth T. (2004). Effect of topically applied resveratrol on cutaneous herpes simplex virus infections in hairless mice. Antivir. Res..

[283] Docherty J.J., Fu M.M., Hah J.M., Sweet T.J., Faith S.A., Booth T. (2005). Effect of resveratrol on herpes simplex virus vaginal infection in the mouse. Antivir. Res..

[284] Faith S.A., Sweet T.J., Bailey E., Booth T., Docherty J.J. (2006). Resveratrol suppresses nuclear factor-κB in herpes simplex virus infected cells. Antivir. Res..

[285] Chen X., Qiao H., Liu T., Yang Z., Xu L., Xu Y., Ge H.M., Tan R.-X., Li E. (2012). Inhibition of herpes simplex virus infection by oligomeric stilbenoids through ROS generation. Antivir. Res..

[286] Wang Z., Cai X., Ren Z., Shao Y., Xu Y., Fu L., Zhu Y. (2023). Piceatannol as an Antiviral Inhibitor of PRV Infection In Vitro and In Vivo. Animals.

[287] Docherty J.J., Sweet T.J., Bailey E., Faith S.A., Booth T. (2006). Resveratrol inhibition of varicella-zoster virus replication in vitro. Antivir. Res..

[288] Berardi V., Ricci F., Castelli M., Galati G., Risuleo G. (2009). Resveratrol exhibits a strong cytotoxic activity in cultured cells and has an antiviral action against polyomavirus: Potential clinical use. J. Exp. Clin. Cancer Res..

[289] Aspinall E.J., Hawkins G., Fraser A., Hutchinson S.J., Goldberg D. (2011). Hepatitis B prevention, diagnosis, treatment and care: A review. Occup. Med..

[290] Trépo C., Chan H.L., Lok A. (2014). Hepatitis B virus infection. Lancet.

[291] Safioleas M., Lygidakis N.J., Manti C. (2007). Hepatitis B today. Hepatogastroenterology.

[292] Wilkins T., Sams R., Carpenter M. (2019). Hepatitis B: Screening, Prevention, Diagnosis, and Treatment. Am. Fam. Physician.

[293] Duff P. (1998). Hepatitis in pregnancy. Semin. Perinatol..

[294] Strang B.L. (2025). Inhibition of human cytomegalovirus replication by valaciclovir. J. Gen. Virol..

[295] Schleiss M.R. (2025). The urgent search for predictive biomarkers in the emerging era of universal congenital cytomegalovirus screening. Philos. Trans. R. Soc. Lond. B Biol. Sci..

[296] Zhang T., Chen T., Tang X. (2025). Ménétrier’s Disease: A Narrative Review of Molecular Pathogenesis, Clinical Spectrum, and Evolving Therapeutic Strategies. Qjm Int. J. Med..

[297] Sato N., Shiraki A., Daikoku T., Takemoto M., Takemura Y., Sakai K., Imoto S., Yanagita M., Tanabe K., Shiraki K. (2025). Mechanistic insights into the inhibition of drug-resistant cytomegalovirus by letermovir and ganciclovir. Br. J. Pharmacol..

[298] Kiliç Güneş E., Afacan Öztürk H.B., Kiriktir E., Dikyar A., Uğur B., Güneş A.K., Ayli M. (2025). Clinical characteristics and outcomes in Epstein-Barr virus-positive diffuse large B-cell lymphoma: A multicenter retrospective study. Turk. J. Med. Sci..

[299] Wang Z., Chang X., Cheng X. (2025). Epstein-Barr virus in thyroid disease: An integrated immunovirological perspective. Front. Immunol..

[300] Li Z.Y., Wang R., Chen X.Z. (2025). Causal association between local infection of Epstein-Barr virus and gastric cancer risk: A rapid meta-analysis. Chin. Clin. Oncol..

[301] Xie T., Shi C. (2025). Recent advances in infections as risk factors for lymphoma: A review of the effects of viral, bacterial, and fungal infections in lymphoma. Arch. Microbiol..

[302] Younis S., Moutusy S.I., Rasouli S., Jahanbani S., Pandit M., Wu X., Acharya S., Sharpe O., Wijeratne T.U., Harris M.L. (2025). Epstein-Barr virus reprograms autoreactive B cells as antigen-presenting cells in systemic lupus erythematosus. Sci. Transl. Med..

[303] Gorochov G., Mathian A. (2025). EBV and the making of lupus. Sci. Transl. Med..

[304] Landy H.J., Grossman J.H. (1989). Herpes simplex virus. Obs. Gynecol. Clin. N. Am..

[305] Fatahzadeh M., Schwartz R.A. (2007). Human herpes simplex virus infections: Epidemiology, pathogenesis, symptomatology, diagnosis, and management. J. Am. Acad. Dermatol..

[306] Omarova S., Cannon A., Weiss W., Bruccoleri A., Puccio J. (2022). Genital Herpes Simplex Virus-An Updated Review. Adv. Pediatr..

[307] Sibley D., Larkin D.F.P. (2020). Update on Herpes simplex keratitis management. Eye.

[308] Samies N.L., James S.H., Kimberlin D.W. (2021). Neonatal Herpes Simplex Virus Disease: Updates and Continued Challenges. Clin. Perinatol..

[309] Agelidis A., Koujah L., Suryawanshi R., Yadavalli T., Mishra Y.K., Adelung R., Shukla D. (2019). An Intra-Vaginal Zinc Oxide Tetrapod Nanoparticles (ZOTEN) and Genital Herpesvirus Cocktail Can Provide a Novel Platform for Live Virus Vaccine. Front. Immunol..

[310] White M.K., Khalili K. (2004). Polyomaviruses and human cancer: Molecular mechanisms underlying patterns of tumorigenesis. Virology.

[311] Senay T.E., Li X., Shirhattikar S.G., Luo T.T., You J. (2025). A short intrinsically disordered domain of MCPyV ALTO regulates TBK1 signaling during MCPyV infection. J. Virol..

[312] Cook L. (2016). Polyomaviruses. Microbiol. Spectr..

[313] Arvin A.M. (1996). Varicella-zoster virus. Clin. Microbiol. Rev..

[314] Cohen J.I. (2013). Clinical practice: Herpes zoster. N. Engl. J. Med..

[315] Gershon A.A., Breuer J., Cohen J.I., Cohrs R.J., Gershon M.D., Gilden D., Grose C., Hambleton S., Kennedy P.G., Oxman M.N. (2015). Varicella zoster virus infection. Nat. Rev. Dis. Primers.

[316] Kouzeli A., Hasan T., Smith K., Metelmann S., Gibbons C., Ghebrehewet S. (2025). Impact of vaccination on shingles-related hospitalisations in 70–79-year-olds in Scotland between 2000 and 2023: A retrospective cohort study. Vaccine.

[317] Lee D.E., Cha H.R., Lee Y.L., Chung H.W., Park S., Oh Y.J., Jung E., Baek S.K., Ahn B.C., Yum J.S. (2025). Development and evaluation of recombinant Varicella-Zoster virus glycoprotein E protein microneedles for enhanced pediatric vaccine delivery. Drug Deliv. Transl. Res..

[318] McCarthy M.K., Morrison T.E. (2017). Persistent RNA virus infections: Do PAMPS drive chronic disease?. Curr. Opin. Virol..

[319] Debat H., Bejerman N. (2025). An Update on RNA Virus Discovery: Current Challenges and Future Perspectives. Viruses.

[320] Aw D.Z.H., Zhang D.X., Vignuzzi M. (2025). Strategies and efforts in circumventing the emergence of antiviral resistance against conventional antivirals. npj Antimicrob. Resist..

[321] Ter Ellen B.M., Dinesh Kumar N., Bouma E.M., Troost B., van de Pol D.P.I., van der Ende-Metselaar H.H., Apperloo L., van Gosliga D., van den Berge M., Nawijn M.C. (2021). Resveratrol and Pterostilbene Inhibit SARS-CoV-2 Replication in Air-Liquid Interface Cultured Human Primary Bronchial Epithelial Cells. Viruses.

[322] Pasquereau S., Nehme Z., Haidar Ahmad S., Daouad F., Van Assche J., Wallet C., Schwartz C., Rohr O., Morot-Bizot S., Herbein G. (2021). Resveratrol Inhibits HCoV-229E and SARS-CoV-2 Coronavirus Replication In Vitro. Viruses.

[323] Nawrot-Hadzik I., Zmudzinski M., Matkowski A., Preissner R., Kęsik-Brodacka M., Hadzik J., Drag M., Abel R. (2021). Reynoutria Rhizomes as a Natural Source of SARS-CoV-2 Mpro Inhibitors-Molecular Docking and In Vitro Study. Pharmaceuticals.

[324] Nakamura M., Saito H., Ikeda M., Hokari R., Kato N., Hibi T., Miura S. (2010). An antioxidant resveratrol significantly enhanced replication of hepatitis C virus. World J. Gastroenterol..

[325] Palamara A.T., Nencioni L., Aquilano K., De Chiara G., Hernandez L., Cozzolino F., Ciriolo M.R., Garaci E. (2005). Inhibition of influenza A virus replication by resveratrol. J. Infect. Dis..

[326] Gupta C.L., Akhtar S., Bajpaib P., Kandpal K.N., Desai G.S., Tiwari A.K. (2013). Computational modeling and validation studies of 3-D structure of neuraminidase protein of H_1_N_1_ influenza A virus and subsequent in silico elucidation of piceid analogues as its potent inhibitors. Excli J..

[327] Huang L., Wang J., Ma X., Sun L., Hao C., Wang W. (2023). Inhibition of influenza a virus infection by natural stilbene piceatannol targeting virus hemagglutinin. Phytomedicine.

[328] Wu W., Ye Y., Zhong Y., Yan X., Lin J., Qiu J., Liu S., Fang Z. (2023). Pterostilbene effectively inhibits influenza A virus infection by promoting the type I interferon production. Microbes Infect..

[329] Zhang Y., Li J., Qiu Z., Huang L., Yang S., Li J., Li K., Liang Y., Liu X., Chen Z. (2024). Insights into the mechanism of action of pterostilbene against influenza A virus-induced acute lung injury. Phytomedicine.

[330] Zhang L., Li Y., Gu Z., Wang Y., Shi M., Ji Y., Sun J., Xu X., Zhang L., Jiang J. (2015). Resveratrol inhibits enterovirus 71 replication and pro-inflammatory cytokine secretion in rhabdosarcoma cells through blocking IKKs/NF-κB signaling pathway. PLoS ONE.

[331] Chuang K.T., Pan S.C., Chiang B.L., Chen S.H., Pan M.H., Chen Y.L., Lin C.S., Pan C.K., Lin J.Y., Lin Y.L. (2025). Pterostilbene Exhibits Broad-Spectrum Antiviral Activity by Targeting the Enterovirus Capsid, Inactivating Viral Particles, Blocking Viral Binding, and Protecting Mice From Lethal EV-A71 Challenge. Phytother. Res..

[332] Mastromarino P., Capobianco D., Cannata F., Nardis C., Mattia E., De Leo A., Restignoli R., Francioso A., Mosca L. (2015). Resveratrol inhibits rhinovirus replication and expression of inflammatory mediators in nasal epithelia. Antivir. Res..

[333] Sato F., Martinez N.E., Shahid M., Rose J.W., Carlson N.G., Tsunoda I. (2013). Resveratrol exacerbates both autoimmune and viral models of multiple sclerosis. Am. J. Pathol..

[334] Komaravelli N., Kelley J.P., Garofalo M.P., Wu H., Casola A., Kolli D. (2015). Role of dietary antioxidants in human metapneumovirus infection. Virus Res..

[335] Zang N., Xie X., Deng Y., Wu S., Wang L., Peng C., Li S., Ni K., Luo Y., Liu E. (2011). Resveratrol-mediated gamma interferon reduction prevents airway inflammation and airway hyperresponsiveness in respiratory syncytial virus-infected immunocompromised mice. J. Virol..

[336] Xie X.-H., Zang N., Li S.-M., Wang L.-J., Deng Y., He Y., Yang X.-Q., Liu E.-M. (2012). Resveratrol Inhibits Respiratory Syncytial Virus-Induced IL-6 Production, Decreases Viral Replication, and Downregulates TRIF Expression in Airway Epithelial Cells. Inflammation.

[337] Cheng K., Wu Z., Gao B., Xu J. (2014). Analysis of influence of baicalin joint resveratrol retention enema on the TNF-α, SIgA, IL-2, IFN-γ of rats with respiratory syncytial virus infection. Cell Biochem. Biophys..

[338] Long X., Li S., Xie J., Li W., Zang N., Ren L., Deng Y., Xie X., Wang L., Fu Z. (2015). MMP-12-mediated by SARM-TRIF signaling pathway contributes to IFN-γ-independent airway inflammation and AHR post RSV infection in nude mice. Respir. Res..

[339] Zang N., Li S., Li W., Xie X., Ren L., Long X., Xie J., Deng Y., Fu Z., Xu F. (2015). Resveratrol suppresses persistent airway inflammation and hyperresponsivess might partially via nerve growth factor in respiratory syncytial virus-infected mice. Int. Immunopharmacol..

[340] Heredia A., Davis C., Amin M.N., Le N.M., Wainberg M.A., Oliveira M., Deeks S.G., Wang L.X., Redfield R.R. (2014). Targeting host nucleotide biosynthesis with resveratrol inhibits emtricitabine-resistant HIV-1. Aids.

[341] Zhu N., Zhang D., Wang W., Li X., Yang B., Song J., Zhao X., Huang B., Shi W., Lu R. (2020). A Novel Coronavirus from Patients with Pneumonia in China, 2019. N. Engl. J. Med..

[342] V’Kovski P., Kratzel A., Steiner S., Stalder H., Thiel V. (2021). Coronavirus biology and replication: Implications for SARS-CoV-2. Nat. Rev. Microbiol..

[343] Tay M.Z., Poh C.M., Rénia L., MacAry P.A., Ng L.F.P. (2020). The trinity of COVID-19: Immunity, inflammation and intervention. Nat. Rev. Immunol..

[344] Daia C., Scheau C., Neagu G., Andone I., Spanu A., Popescu C., Stoica S.I., Verenca M.C., Onose G. (2020). Nerve conduction study and electromyography findings in patients recovering from Covid-19—Case report. Int. J. Infect. Dis..

[345] Shih A.R., Misdraji J. (2023). COVID-19: Gastrointestinal and hepatobiliary manifestations. Hum. Pathol..

[346] Borges P.H.O., Gras E., Ferreira S.B., Paes Silva F. (2025). Hepatitis C virus (HCV) proteases: Structure, function and inhibition strategies. Enzymes.

[347] Hashem H.R., Yehia T., Azab M., Abdellah A., Amin I.A., Salah M., Ramadan M. (2025). Gut microbiome dysbiosis in hepatocellular carcinoma patients with persistent HCV viremia versus viral clearance: A cross-sectional study. Gut Pathog..

[348] Mathew M., Nguyen M., Jolly K., Rangaswamy B., Stroever S. (2025). Hepatitis C virus-related hepatocellular carcinoma mortality: A focus on the US-Mexico border region. Bayl. Univ. Med Cent. Proc..

[349] Gaitonde D.Y., Moore F.C., Morgan M.K. (2019). Influenza: Diagnosis and treatment. Am. Fam. Physician.

[350] Świerczyńska M., Mirowska-Guzel D.M., Pindelska E. (2022). Antiviral drugs in influenza. Int. J. Environ. Res. Public Health.

[351] Wu N.C., Wilson I.A. (2020). Influenza hemagglutinin structures and antibody recognition. Cold Spring Harb. Perspect. Med..

[352] Webster R.G., Govorkova E.A. (2014). Continuing challenges in influenza. Ann. N. Y. Acad. Sci..

[353] Tuthill T.J., Groppelli E., Hogle J.M., Rowlands D.J. (2010). Picornaviruses. Curr. Top. Microbiol. Immunol..

[354] Jubelt B., Lipton H.L. (2014). Enterovirus/picornavirus infections. Handb. Clin. Neurol..

[355] Rodriguez-Morales A.J., Rodriguez-Morales A.G. (2025). Enterovirus Meningitis. Methods Mol. Biol..

[356] Helfferich J., Knoester M., Van Leer-Buter C.C., Neuteboom R.F., Meiners L.C., Niesters H.G., Brouwer O.F. (2019). Acute flaccid myelitis and enterovirus D68: Lessons from the past and present. Eur. J. Pediatr..

[357] Hooi Y.T., Fu T.L., Tan S.H., Ong K.C., Tan C.Y., Wong K.T. (2025). Neuroinvasion via Peripheral Nerves in Epidemic Viral Encephalitis Caused by Enterovirus, Orthoflavivirus and SARS-Coronavirus. Neuropathol. Appl. Neurobiol..

[358] Helgers L.C., Vlaming K.E., Kaptein T.M., Eder J., Duitman J.W., Geijtenbeek T.B. (2025). Heparin Provides Antiviral Activity Against Rhinovirus-16 via an Heparan Sulfate Proteoglycan-Independent Mechanism. Int. J. Mol. Sci..

[359] Jurkowicz M., Solomovich M., Leibovitz E., Keller N., Yahav D., Barkai G., Atari N., Fratty I.S., Cohen H., Belkin A. (2025). Clinical characteristics, outcomes, and subtype diversity in hospitalized human rhinovirus (HRV) patients. PLoS ONE.

[360] Lee J., Lee H.L., Kim H., Gil Y., Lee S.-H., Jung Y.-S., Shin J.S., Jo I. (2026). Structural insights into the antiviral efficacy of AG7404 against human rhinovirus 3C proteases. IUCrJ.

[361] Kang T.W., Perez-Gomez A., Lawley K., Young C.R., Welsh C.J., Brinkmeyer-Langford C.L. (2025). Comparative Analysis of Genetic Risk for Viral-Induced Axonal Loss in Genetically Diverse Mice. Int. J. Mol. Sci..

[362] Ahmad I., Omura S., Khadka S., Sato F., Park A.-M., Rimal S., Tsunoda I. (2025). Gut Microbiota in a Viral Model of Multiple Sclerosis: Modulation and Pitfalls by Oral Antibiotic Treatment. Cells.

[363] Sharma R., Walia A., Lakhanpal D. (2025). Human metapneumovirus: An underdiagnosed public health threat. Infect. Dis. Now.

[364] Agarwal U., Paliwal S., Tonk R.K. (2025). A Comprehensive Review on Human Metapneumovirus. Curr. Gene Ther..

[365] Gao G., Lin R., Ma D. (2025). Human metapneumovirus: Pathogenesis, epidemiology, diagnostic technologies, and potential intervention strategies. Virol. J..

[366] Collins P.L., Melero J.A. (2011). Progress in understanding and controlling respiratory syncytial virus: Still crazy after all these years. Virus Res..

[367] Hall C.B., Weinberg G.A., Iwane M.K., Blumkin A.K., Edwards K.M., Staat M.A., Auinger P., Griffin M.R., Poehling K.A., Erdman D. (2009). The burden of respiratory syncytial virus infection in young children. N. Engl. J. Med..

[368] Graham B.S. (2016). Vaccines against respiratory syncytial virus: The time has finally come. Vaccine.

[369] Liu T., Zang N., Zhou N., Li W., Xie X., Deng Y., Ren L., Long X., Li S., Zhou L. (2014). Resveratrol inhibits the TRIF-dependent pathway by upregulating sterile alpha and armadillo motif protein, contributing to anti-inflammatory effects after respiratory syncytial virus infection. J. Virol..

[370] Barré-Sinoussi F., Chermann J.C., Rey F., Nugeyre M.T., Chamaret S., Gruest J., Dauguet C., Axler-Blin C., Vézinet-Brun F., Rouzioux C. (1983). Isolation of a T-lymphotropic retrovirus from a patient at risk for acquired immune deficiency syndrome (AIDS). Science.

[371] Deeks S.G., Overbaugh J., Phillips A., Buchbinder S. (2015). HIV infection. Nat. Rev. Dis. Primers.

[372] Arts E.J., Hazuda D.J. (2012). HIV-1 antiretroviral drug therapy. Cold Spring Harb. Perspect. Med..

[373] van Heuvel Y., Schatz S., Rosengarten J.F., Stitz J. (2022). Infectious RNA: Human Immunodeficiency Virus (HIV) Biology, Therapeutic Intervention, and the Quest for a Vaccine. Toxins.

[374] Jiang Y.-L., Liu Z.-P. (2011). Natural products as anti-invasive and anti-metastatic agents. Curr. Med. Chem..

[375] Park E.-J., Pezzuto J.M. (2015). The pharmacology of resveratrol in animals and humans. Biochim. Et Biophys. Acta (BBA)-Mol. Basis Dis..

[376] Seyed M.A., Jantan I., Bukhari S.N., Vijayaraghavan K. (2016). A Comprehensive Review on the Chemotherapeutic Potential of Piceatannol for Cancer Treatment, with Mechanistic Insights. J. Agric. Food Chem..

[377] Mizuhara N., Inoue M., Kurotaki H., Matsumoto K., Ogita A., Fujita K.-I. (2023). Pterostilbene, a Natural Methoxylated Analog of Resveratrol, Exhibits Antifungal Activity Induced by Reactive Oxygen Species Production and Plasma Membrane Injury. Appl. Microbiol..

[378] Langcake P., Pryce R. (1976). The production of resveratrol by Vitis vinifera and other members of the Vitaceae as a response to infection or injury. Physiol. Plant Pathol..

[379] Langcake P., McCarthy W.V. (1979). The relationship between resveratrol production to infection of grapevine leaves by Botrytis cinerea. Vitis.

[380] Langcake P. (1981). Disease resistance of Vitis spp. and the production of the stress metabolites resveratrol, ε-viniferin, α-viniferin and pterostilbene. Physiol. Plant Pathol..

[381] Pool R., Creasy L., Frackelton A.S. (1981). Resveratrol and the viniferins, their application to screening for disease resistance in grape breeding programs. Vitis.

[382] Langcake P., Cornford C., Pryce R. (1979). Identification of pterostilbene as a phytoalexin from Vitis vinifera leaves. Phytochemistry.

[383] Hill G.N., Jaksons P., Sharp J.M., Hunt A.G., Lewis K.S.J. (2019). Investigating time and economic costs of botrytis bunch rot sampling using interpolated data. N. Zealand Plant Prot..

[384] Dwivedi M., Singh P., Pandey A.K. (2024). Botrytis fruit rot management: What have we achieved so far?. Food Microbiol..

[385] Docherty J.J., Fu M.M., Tsai M. (2001). Resveratrol selectively inhibits Neisseria gonorrhoeae and Neisseria meningitidis. J. Antimicrob. Chemother..

[386] Liu Z., Zhuang C., Sheng S., Shao L., Zhao W., Zhao S. (2011). Overexpression of a resveratrol synthase gene (PcRS) from Polygonum cuspidatum in transgenic Arabidopsis causes the accumulation of trans-piceid with antifungal activity. Plant Cell Rep..

[387] Yan Y., Yuan Q., Tang J., Huang J., Hsiang T., Wei Y., Zheng L. (2018). *Colletotrichum higginsianum* as a Model for Understanding Host–Pathogen Interactions: A Review. Int. J. Mol. Sci..

[388] Damm U., O’Connell R.J., Groenewald J.Z., Crous P.W. (2014). The *Colletotrichum destructivum* species complex—Hemibiotrophic pathogens of forage and field crops. Stud. Mycol..

[389] Chaabane F., Graf A., Jequier L., Coste A.T. (2019). Review on Antifungal Resistance Mechanisms in the Emerging Pathogen *Candida auris*. Front. Microbiol..

[390] Carty J., Chowdhary A., Bernstein D., Thangamani S. (2023). Tools and techniques to identify, study, and control *Candida auris*. PLoS Pathog..

[391] Cadnum J.L., Shaikh A.A., Piedrahita C.T., Sankar T., Jencson A.L., Larkin E.L., Ghannoum M.A., Donskey C.J. (2017). Effectiveness of Disinfectants Against *Candida auris* and Other *Candida* Species. Infect. Control Hosp. Epidemiol..

[392] Calegari-Alves Y.P., Costa R.P., Innocente-Alves C., Soares G.d.N., Lima E.S., Saciloto-de-Oliveira L.R., Alves L.R., Vainstein M.H., Beys-da-Silva W.O., Santi L. (2025). A review of bioactive plant compounds against WHO priority fungal pathogens. Microb. Pathog..

[393] Chen Y., Xiao Z. (2013). Therapeutic effect of resveratrol as well as resveratrol combined with praziquantel on the liver fibrosis due to Schistosoma japonicum infection in mice. Zhongguo Ji Sheng Chong Xue Yu Ji Sheng Chong Bing Za Zhi.

[394] Morais P., Piazzon C., Lamas J., Mallo N., Leiro J.M. (2013). Effect of resveratrol on oxygen consumption by *Philasterides dicentrarchi*, a scuticociliate parasite of turbot. Protist.

[395] Valle A., Leiro J.M., Pereiro P., Figueras A., Novoa B., Dirks R.P.H., Lamas J. (2020). Interactions between the Parasite *Philasterides dicentrarchi* and the Immune System of the Turbot *Scophthalmus maximus*. A Transcriptomic Analysis. Biology.

[396] Roy B., Giri B.R. (2016). α-Viniferin and resveratrol induced alteration in the activities of some energy metabolism related enzymes in the cestode parasite *Raillietina echinobothrida*. Acta Trop..

[397] Gouveia M.J., Nogueira V., Araújo B., Gärtner F., Vale N. (2019). Inhibition of the Formation In Vitro of Putatively Carcinogenic Metabolites Derived from *S. haematobium* and *O. viverrini* by Combination of Drugs with Antioxidants. Molecules.

[398] Geahlen R.L., McLaughlin J.L. (1989). Piceatannol (3,4,3′,5′-tetrahydroxy-trans-stilbene) is a naturally occurring protein-tyrosine kinase inhibitor. Biochem. Biophys. Res. Commun..

[399] del Olmo E., Armas M.G.A., López-Pérez J.L., Muñoz V., Deharo E., San Feliciano A. (2001). Leishmanicidal activity of some stilbenoids and related heterocyclic compounds. Bioorganic Med. Chem. Lett..

[400] Sharma A., Mishra N.C. (1999). Inhibition of a protein tyrosine kinase activity in Plasmodium falciparum by chloroquine. Indian J. Biochem. Biophys..

[401] Mishra N.C., Sharma M., Sharma A. (1999). Inhibitory effect of piceatannol, a protein tyrosine kinase inhibitor, on asexual maturation of Plasmodium falciparum. Indian J. Exp. Biol..

[402] Moro P., Schantz P.M. (2009). Echinococcosis: A review. Int. J. Infect. Dis..

[403] Řežábková L., Brabec J., Jirků M., Dellerba M., Kuchta R., Modrý D., Parker W., Jirků Pomajbíková K. (2019). Genetic diversity of the potentially therapeutic tapeworm *Hymenolepis diminuta* (Cestoda: Cyclophyllidea). Parasitol. Int..

[404] Jahangeer M., Mahmood Z., Munir N., Waraich U.E., Tahir I.M., Akram M., Ali Shah S.M., Zulfqar A., Zainab R. (2020). *Naegleria fowleri*: Sources of infection, pathophysiology, diagnosis, and management; a review. Clin. Exp. Pharmacol. Physiol..

[405] Abdo M.G., Elamin W.M., Khalil E.A., Mukhtar M.M. (2003). Antimony-resistant Leishmania donovaniin eastern Sudan: Incidence and in vitro correlation. East. Mediterr. Health J..

[406] Majumdar D., Elsayed G.A., Buskas T., Boons G.-J. (2005). Synthesis of Proteophosphoglycans of *Leishmania major* and *Leishmania mexicana*. J. Org. Chem..

[407] Alvar J., Vélez I.D., Bern C., Herrero M., Desjeux P., Cano J., Jannin J., Boer M.D., Team W.L.C. (2012). Leishmaniasis worldwide and global estimates of its incidence. PLoS ONE.

[408] Greenwood B. (2004). The use of anti-malarial drugs to prevent malaria in the population of malaria-endemic areas. Am. J. Trop. Med. Hyg..

[409] Autino B., Noris A., Russo R., Castelli F. (2012). Epidemiology of malaria in endemic areas. Mediterr. J. Hematol. Infect. Dis..

[410] Tavakoli Pirzaman A., Sepidarkish M., Alizadeh F., Al-Obidy S., Ebrahimi P., Kianifard N., Sheikhi Nooshabadi M., Jafari Tadi M., Zolfaghari Dehkharghani M., Mousavi S. (2024). Prevalence of human *Schistosoma mansoni* infection in endemic regions (2010–2024): A systematic review and meta-analysis. EClinicalMedicine.

[411] Tong W.H., Pavey C., O’Handley R., Vyas A. (2021). Behavioral biology of Toxoplasma gondii infection. Parasites Vectors.

[412] Dupouy-Camet J. (2000). Trichinellosis: A worldwide zoonosis. Vet. Parasitol..

[413] Noireau F., Diosque P., Jansen A.M. (2009). Trypanosoma cruzi: Adaptation to its vectors and its hosts. Vet. Res..

[414] Pflieger A., Waffo Teguo P., Papastamoulis Y., Chaignepain S., Subra F., Munir S., Delelis O., Lesbats P., Calmels C., Andreola M.L. (2013). Natural stilbenoids isolated from grapevine exhibiting inhibitory effects against HIV-1 integrase and eukaryote MOS1 transposase in vitro activities. PLoS ONE.

[415] Koonin E.V., Krupovic M., Agol V.I. (2021). The Baltimore Classification of Viruses 50 Years Later: How Does It Stand in the Light of Virus Evolution?. Microbiol. Mol. Biol. Rev..

[416] Cottart C.H., Nivet-Antoine V., Laguillier-Morizot C., Beaudeux J.L. (2010). Resveratrol bioavailability and toxicity in humans. Mol. Nutr. Food Res..

[417] Almeida L., Vaz-da-Silva M., Falcão A., Soares E., Costa R., Loureiro A.I., Fernandes-Lopes C., Rocha J.F., Nunes T., Wright L. (2009). Pharmacokinetic and safety profile of trans-resveratrol in a rising multiple-dose study in healthy volunteers. Mol. Nutr. Food Res..

[418] Boocock D.J., Patel K.R., Faust G.E., Normolle D.P., Marczylo T.H., Crowell J.A., Brenner D.E., Booth T.D., Gescher A., Steward W.P. (2007). Quantitation of trans-resveratrol and detection of its metabolites in human plasma and urine by high performance liquid chromatography. J. Chromatogr. B Anal. Technol. Biomed. Life Sci..

[419] Brown V.A., Patel K.R., Viskaduraki M., Crowell J.A., Perloff M., Booth T.D., Vasilinin G., Sen A., Schinas A.M., Piccirilli G. (2010). Repeat dose study of the cancer chemopreventive agent resveratrol in healthy volunteers: Safety, pharmacokinetics, and effect on the insulin-like growth factor axis. Cancer Res..

[420] Reinisalo M., Kårlund A., Koskela A., Kaarniranta K., Karjalainen R.O. (2015). Polyphenol Stilbenes: Molecular Mechanisms of Defence against Oxidative Stress and Aging-Related Diseases. Oxid. Med. Cell. Longev..

[421] Wang P., Sang S. (2018). Metabolism and pharmacokinetics of resveratrol and pterostilbene. Biofactors.

[422] Setoguchi Y., Oritani Y., Ito R., Inagaki H., Maruki-Uchida H., Ichiyanagi T., Ito T. (2014). Absorption and metabolism of piceatannol in rats. J. Agric. Food Chem..

[423] Patel K.R., Brown V.A., Jones D.J., Britton R.G., Hemingway D., Miller A.S., West K.P., Booth T.D., Perloff M., Crowell J.A. (2010). Clinical pharmacology of resveratrol and its metabolites in colorectal cancer patients. Cancer Res..

[424] Stegemann S., Leveiller F., Franchi D., De Jong H., Lindén H. (2007). When poor solubility becomes an issue: From early stage to proof of concept. Eur. J. Pharm. Sci..

[425] Sharapova A., Ol’khovich M., Blokhina S., Perlovich G.L. (2022). Experimental Examination of Solubility and Lipophilicity as Pharmaceutically Relevant Points of Novel Bioactive Hybrid Compounds. Molecules.

[426] Liu K., Zhao P., Chen Y., Zhang Y., Jin J., Wan T. (2025). Engineering micelles with hydrophilic-lipophilic balance to overcome intestinal barrier for oral therapeutic application. Mater. Today Bio.

[427] Křen V., Fraser-Reid B.O., Tatsuta K., Thiem J. (2008). Glycoside vs. Aglycon: The Role of Glycosidic Residue in Biological Activity. Glycoscience: Chemistry and Chemical Biology.

[428] Watal G., Watal A., Rai P.K., Rai D.K., Sharma G., Sharma B. (2013). Biomedical applications of nano-antioxidant. Methods Mol. Biol..

[429] Walle T. (2011). Bioavailability of resveratrol. Ann N Y Acad Sci.

[430] Kapetanovic I.M., Muzzio M., Huang Z., Thompson T.N., McCormick D.L. (2011). Pharmacokinetics, oral bioavailability, and metabolic profile of resveratrol and its dimethylether analog, pterostilbene, in rats. Cancer Chemother. Pharmacol..

[431] Amri A., Chaumeil J.C., Sfar S., Charrueau C. (2012). Administration of resveratrol: What formulation solutions to bioavailability limitations?. J. Control. Release.

[432] Ogas T., Kondratyuk T.P., Pezzuto J.M. (2013). Resveratrol analogs: Promising chemopreventive agents. Ann. N. Y. Acad. Sci..

[433] Matei A.-M., Caruntu C., Tampa M., Georgescu S.R., Matei C., Constantin M.M., Constantin T.V., Calina D., Ciubotaru D.A., Badarau I.A. (2021). Applications of Nanosized-Lipid-Based Drug Delivery Systems in Wound Care. Appl. Sci..

[434] Zhen X., Xie C., Jiang Y., Ai X., Xing B., Pu K. (2018). Semiconducting Photothermal Nanoagonist for Remote-Controlled Specific Cancer Therapy. Nano Lett..

[435] Xu M., Zhang J., Mu Y., Foda M.F., Han H. (2022). Activation of TRPV1 by capsaicin-loaded CaCO_3_ nanoparticle for tumor-specific therapy. Biomaterials.

[436] Dragosloveanu S., Vulpe D.E., Andrei C.A., Nedelea D.-G., Garofil N.D., Anghel C., Dragosloveanu C.D.M., Cergan R., Scheau C. (2025). Predicting periprosthetic joint Infection: Evaluating supervised machine learning models for clinical application. J. Orthop. Transl..

[437] Viscopoleanu G., Valeanu M.-S., Capitanu B.-S., Dragosloveanu S., Scheau C. (2025). Periprosthetic Joint Infection by Streptococcus bovis Reveals Hidden Colorectal Cancer: A Case Report. Life.

[438] Noor S., Megaloikonomos P.D., Abedi A.A., Abbaszadeh A., Adi M.M., Al-Farii H., Aliyev H., Alizade C., Shoaib bin Shakeel M., Borjón E.D. (2025). 2025 ICM: Diagnostic Imaging for Periprosthetic Joint Infection. J. Arthroplast..

[439] Birlutiu R.-M., Salimi M., Dragosloveanu S., Scheau C., Vorovenci A.E., Larie A., Anea E.-C., Neamtu B., Birlutiu V. (2025). Diagnostic Performance of Serum Neutrophil–Lymphocyte and Serum Monocyte–Lymphocyte Ratios in Periprosthetic Joint Infection: A Comparative Meta-Analytic Review of 29 Studies. J. Clin. Med..

[440] Kortlever J., Rainey J., Alencar P., Anderson L., Busato T., Chirveches L., Dragosloveanu S., Goosen J., Perez J., Higuera C. (2025). 2025 ICM: Antibiotic Prophylaxis in Revision Arthroplasty—Does extended antibiotic prophylaxis reduce periprosthetic joint infection (PJI) rate in patients undergoing aseptic hip or knee revision arthroplasty?. J. Arthroplast..

[441] Cristea S., Predescu V., Dragosloveanu S., Cuculici S., Marandici N. (2016). Surgical Approaches for Total Knee Arthroplasty. Arthroplasty—A Comprehensive Review.

[442] Chae S., Cho D.-W. (2023). Biomaterial-based 3D bioprinting strategy for orthopedic tissue engineering. Acta Biomater..

[443] Periferakis A., Periferakis A.-T., Troumpata L., Dragosloveanu S., Timofticiuc I.-A., Georgatos-Garcia S., Scheau A.-E., Periferakis K., Caruntu A., Badarau I.A. (2024). Use of Biomaterials in 3D Printing as a Solution to Microbial Infections in Arthroplasty and Osseous Reconstruction. Biomimetics.

[444] Timofticiuc I.-A., Dragosloveanu S., Caruntu A., Scheau A.-E., Badarau I.A., Garofil N.D., Didilescu A.C., Caruntu C., Scheau C. (2024). 3D Bioprinting in Limb Salvage Surgery. J. Funct. Biomater..

[445] Shahbazi M., Jäger H., Ettelaie R. (2022). Dual-Grafting of Microcrystalline Cellulose by Tea Polyphenols and Cationic ε-Polylysine to Tailor a Structured Antimicrobial Soy-Based Emulsion for 3D Printing. ACS Appl. Mater. Interfaces.

[446] Timofticiuc I.-A., Caruntu A., Dragosloveanu C.D.M., Scheau A.-E., Badarau I.A., Periferakis A., Dragosloveanu S., Didilescu A.C., Caruntu C., Scheau C. (2025). Head and Neck 3D Bioprinting—A Review on Recent Advancements in Soft Tissue 3D Bioprinting and Medical Applications. J. Funct. Biomater..

[447] Dong V., Nanchal R., Karvellas C.J. (2020). Pathophysiology of acute liver failure. Nutr. Clin. Pract..

[448] Pinzani M. (2015). Pathophysiology of Liver Fibrosis. Dig. Dis..

[449] Periferakis A., Tsigas G., Periferakis A.-T., Badarau I.A., Scheau A.-E., Tampa M., Georgescu S.R., Didilescu A.C., Scheau C., Caruntu C. (2021). Antitumoral and Anti-inflammatory Roles of Somatostatin and Its Analogs in Hepatocellular Carcinoma. Anal. Cell. Pathol..

[450] Periferakis A., Tsigas G., Periferakis A.T., Tone C.M., Hemes D.A., Periferakis K., Troumpata L., Badarau I.A., Scheau C., Caruntu A. (2024). Agonists, Antagonists and Receptors of Somatostatin: Pathophysiological and Therapeutical Implications in Neoplasias. Curr. Issues Mol. Biol..

[451] Scheau A.-E., Jurca S.O., Scheau C., Lupescu I.G. (2024). Non-Surgical Treatment for Hepatocellular Carcinoma: What to Expect at Follow-Up Magnetic Resonance Imaging—A Pictorial Review. Appl. Sci..

[452] Pecyna P., Wargula J., Murias M., Kucinska M. (2020). More than resveratrol: New insights into stilbene-based compounds. Biomolecules.

[453] Ahmad I. (2018). Tamoxifen a pioneering drug: An update on the therapeutic potential of tamoxifen derivatives. Eur. J. Med. Chem..

[454] Karim A., Raees S., Shah S.A., Gouvinhas I., Novo Barros A. (2025). Stilbenes: Emerging Applications in Health, Agriculture, and Industry. Exploring Natural Phenolic Compounds—Recent Progress and Practical Applications.

[455] Aryati W.D., Nadhira A., Febianli D., Fransisca F., Mun’im A. (2020). Natural deep eutectic solvents ultrasound-assisted extraction (NADES-UAE) of trans-cinnamaldehyde and coumarin from cinnamon bark [*Cinnamomum burmannii* (Nees & T. Nees) Blume]. J. Res. Pharm..

[456] Mansinhos I., Goncalves S., Rodriguez-Solana R., Ordonez-Diaz J.L., Moreno-Rojas J.M., Romano A. (2021). Ultrasonic-assisted extraction and natural deep eutectic solvents combination: A green strategy to improve the recovery of phenolic compounds from *Lavandula pedunculata* subsp. lusitanica (chaytor) franco. Antioxidants.

[457] Taillis D., Pébarthé-Courrouilh A., Lepeltier É., Petit E., Palos-Pinto A., Gabaston J., Mérillon J.-M., Richard T., Cluzet S. (2022). A grapevine by-product extract enriched in oligomerised stilbenes to control downy mildews: Focus on its modes of action towards *Plasmopara viticola*. Oeno One.

[458] Schuh L., Reginato M., Florêncio I., Falcao L., Boron L., Gris E.F., Mello V., Báo S.N. (2023). From nature to innovation: The uncharted potential of natural deep eutectic solvents. Molecules.

[459] Gao Q., Zheng R., Lu J., Li X., Wang D., Cai X., Ren X., Kong Q. (2024). Trends in the Potential of Stilbenes to Improve Plant Stress Tolerance: Insights of Plant Defense Mechanisms in Response to Biotic and Abiotic Stressors. J. Agric. Food Chem..

[460] Aktar M.W., Sengupta D., Chowdhury A. (2009). Impact of pesticides use in agriculture: Their benefits and hazards. Interdiscip. Toxicol..

[461] Stefanidis K., Christopoulou A., Poulos S., Dassenakis E., Dimitriou E. (2020). Nitrogen and Phosphorus Loads in Greek Rivers: Implications for Management in Compliance with the Water Framework Directive. Water.

[462] Tudi M., Daniel Ruan H., Wang L., Lyu J., Sadler R., Connell D., Chu C., Phung D.T. (2021). Agriculture Development, Pesticide Application and Its Impact on the Environment. Int. J. Environ. Res. Public Health.

[463] Muñoz-Bautista J.M., Bernal-Mercado A.T., Martínez-Cruz O., Burgos-Hernández A., López-Zavala A.A., Ruiz-Cruz S., Ornelas-Paz J.D.J., Borboa-Flores J., Ramos-Enríquez J.R., Del-Toro-Sánchez C.L. (2025). Environmental and Health Impacts of Pesticides and Nanotechnology as an Alternative in Agriculture. Agronomy.

[464] Koutsaviti K., Giatropoulos A., Pitarokili D., Papachristos D., Michaelakis A., Tzakou O. (2015). Greek Pinus essential oils: Larvicidal activity and repellency against Aedes albopictus (Diptera: Culicidae). Parasitol. Res..

[465] Sakulpanich A., Attrapadung S., Gritsanapan W. (2017). Insecticidal activity of *Stemona collinsiae* root extract against *Parasarcophaga ruficornis* (Diptera: Sarcophagidae). Acta Trop..

[466] Sakulpanich A., Attrapadung S., Gritsanapan W. (2023). Larvicidal activity of *Stemona collinsiae* root extract against *Musca domestica* and *Chrysomya megacephala*. Sci. Rep..

[467] Zhou W., Li M., Achal V. (2025). A comprehensive review on environmental and human health impacts of chemical pesticide usage. Emerg. Contam..

[468] Ahmad R., Rao R.A.K., Masood M.M. (2005). Removal and Recovery of Cr(VI) from Synthetic and Industrial Wastewater using Bark of *Pinus roxburghii* as an Adsorbent. Water Qual. Res. J..

[469] Sousa S., Jiménez-Guerrero P., Ruiz A., Ratola N., Alves A. (2011). Organochlorine pesticides removal from wastewater by pine bark adsorption after activated sludge treatment. Env. Technol..

[470] Garbowski T., Charazińska S., Pulikowski K., Wiercik P. (2020). Application of microalgae cultivated on pine bark for the treatment of municipal wastewater in cylindrical photobioreactors. Water Environ. J..

[471] Strosnider W.H.J., Llanos López F.S., LaBar J.A., Palmer K.J., Nairn R.W. (2014). Unabated acid mine drainage from Cerro Rico de Potosí, Bolivia: Uncommon constituents of concern impact the Rio Pilcomayo headwaters. Environ. Earth Sci..

[472] Periferakis A., Paresoglou I., Paresoglou N. (2019). The significance of the Lavrion mines in Greek and European Geoheritage. Eur. Geol..

[473] Periferakis A. The Yukon Gold Rush: Early Examples of the Socioeconomic and Environmental Impact of Mining. Proceedings of the 15th International Congress of the Geological Society of Greece.

[474] Periferakis A. (2020). The Keramos Antimonite Mines in Chios Island, Greece: Mining History and Current Situation. News Miner..

[475] Clackett S.P., Porter T.J., Lehnherr I. (2021). The tree-ring mercury record of Klondike gold mining at Bear Creek, central Yukon. Environ. Pollut..

[476] Voudouris P., Melfos V., Mavrogonatos C., Photiades A., Moraiti E., Rieck B., Kolitsch U., Tarantola A., Scheffer C., Morin D. (2021). The Lavrion Mines: A Unique Site of Geological and Mineralogical Heritage. Minerals.

[477] Antoniadis V., Thalassinos G., Levizou E., Wang J., Wang S.-L., Shaheen S.M., Rinklebe J. (2022). Hazardous enrichment of toxic elements in soils and olives in the urban zone of Lavrio, Greece, a legacy, millennia-old silver/lead mining area and related health risk assessment. J. Hazard. Mater..

[478] Bozatzi A., Periferakis A. Geodidactics and CLIL: Synthesizing a syllabus for the ELT classroom through games, stories and technology. Proceedings of the 16th International Congress of the Geological Society of Greece.

